# Axially Chiral Sulfonic Acids for Brønsted Acid Catalysis: 8-Benzoimidazolylnaphthalene-1-sulfonic Acids and Their Derivatives

**DOI:** 10.1021/acs.joc.3c00818

**Published:** 2023-06-24

**Authors:** Monika Tomanová, Iva Vaňková, Daniel Toman, Adam Přibylka, Ivan Nemec, Petr Cankař

**Affiliations:** † Department of Organic Chemistry, Faculty of Science, Palacký University, 17. Listopadu 12, 771 46 Olomouc, Czech Republic; ‡ Department of Inorganic Chemistry, Faculty of Science, Palacký University, 17. Listopadu 12, 771 46 Olomouc, Czech Republic

## Abstract

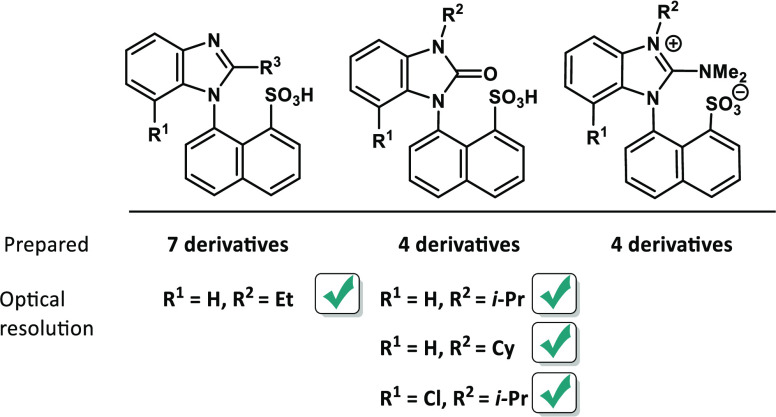

A new type of axially chiral sulfonic acid was developed. The synthesis is based on cheap commercially available materials and a practical method for optical resolution via diastereomeric salt formation, which can provide both enantiomers. Eleven benzoimidazolylnaphthalenesulfonic acids were prepared and four of them were isolated as pure and stable atropisomers. Moreover, several of these sulfonic acids were transformed into triflyl imides to further expand the range of dissociation constants.

## Introduction

Chiral Brønsted acid catalysts play very important roles in stereoselective organic synthesis. A wide range of diverse substrates, which were attractive for stereoselective transformation, have been used to develop a variety of chiral Brønsted acids to modulate a close chiral environment and dissociation constants. Researchers have prepared chiral weak acidic catalysts, activating substrates *via* hydrogen bonding,^1−5^ chiral carboxylic acids,^6^ and stronger, frequently used, BINOL-derived phosphoric acids,^7^ which were introduced by the Akiyama and Terada groups.^8,9^ These phosphoric acids were also functionalized by the triflyl moiety to substantially increase their acidity.^10,11^

**Figure 1:**
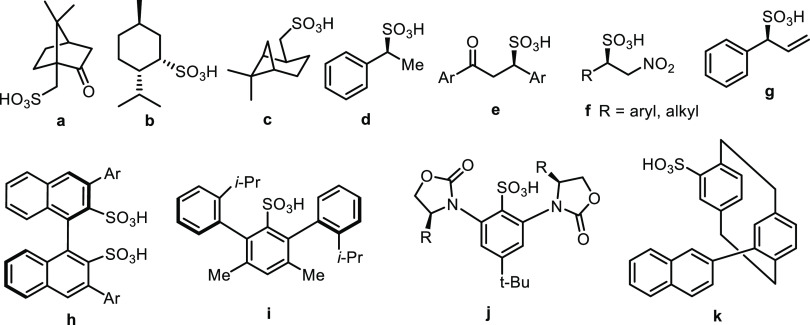
Chiral sulfonic acids.

The development of chiral sulfonic acids can further expand the spectrum of Brønsted catalysts. This class is underexplored, and their syntheses usually lead to cumbersome resolution procedures. The well-known camphorsulfonic acid **a** (Figure 1) is used predominantly for optical resolution *via* diastereomeric salt formation,^12,13^ but its application in the stereoselective synthesis has been described as well.^14^ Other natural-based sulfonic acids **b** and **c** were prepared from (−)-menthol and (−)-*trans*-myrtanol, respectively.^15^ Enantioselective synthesis of (*S*)-1-phenylethane sulfonic acid **d** with excellent enantiomeric purity was reported by Corey.^16^ Bifunctional catalysts were used by Adamo and Blay to prepare chiral sulfonic acids from chalcones or nitroalkenes to afford **e** and **f**, respectively.^17,18^ Similar sulfonic acids **g** with central chirality were obtained by Zhao using asymmetric iridium-catalyzed allylation with sodium sulfite.^19^ The development of chiral sulfonic acids is not limited only to central chirality. The synthesis of optically pure BINOL-derived disulfonic acids **h** (BINSA) was reported by Ishihara.^20^ Furthermore, Blanchet disclosed remarkable bis-*ortho*-arylated axially chiral sulfonic acid **i**,^21^ and a similar benzenesulfonic acid **j** was published by Dixon.^22^ Planar chirality was employed by Enders to synthesize sulfonic acid **k** based on the [2.2]paracyclophane scaffold.^23^

**Figure 2:**
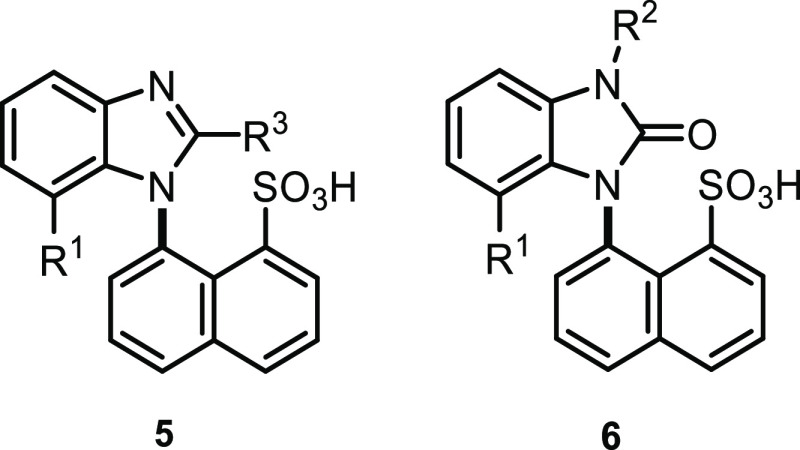
Novel axially chiral sulfonic acids with restricted rotation around the C–N bond (highlighted).

To further enrich the structural variety of chiral sulfonic acids, we designed a synthesis procedure starting from commercially available *peri*-substituted 8-aminonaphthalenesulfonic acid in which the amino group was transformed into a benzimidazole or benzimidazolone ring to generate axial chirality around the C–N bond (Figure 2). We presumed that the benzimidazole or benzimidazolone structure with peripheral substituents would provide the chiral environment for the sulfonic functionality and can modulate the value of the dissociation constant.

## Results and Discussion

**Scheme 1:**
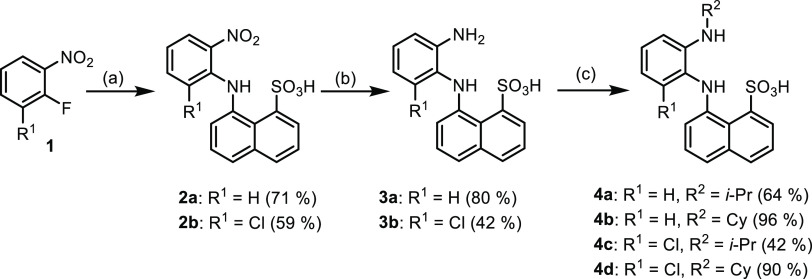
Synthesis of *o*-Phenylenediamine Intermediates **3** and **4** for Cyclization Reactions Reaction conditions: (a) 8-aminonaphthalene-1-sulfonic acid, K_2_CO_3_, dimethyl sulfoxide (DMSO), 130 °C, 4–12 h; (b) H_2_, 10 wt % Pd/C, MeOH; (c) acetone or cyclohexanone, NaBH(OAc)_3_, MeOH, rt, 1.5–22 h.

The synthesis started from commercially available materials (Scheme 1), which were joined by aromatic nucleophilic substitution to afford intermediate **2**. The reaction was accomplished after 4–12 h depending on R^1^ substitution. The catalytic reduction provided diamino derivatives **3**, which can be further transformed by reductive alkylation to *N*-alkylphenylenediamines **4**.

**Scheme 2:**
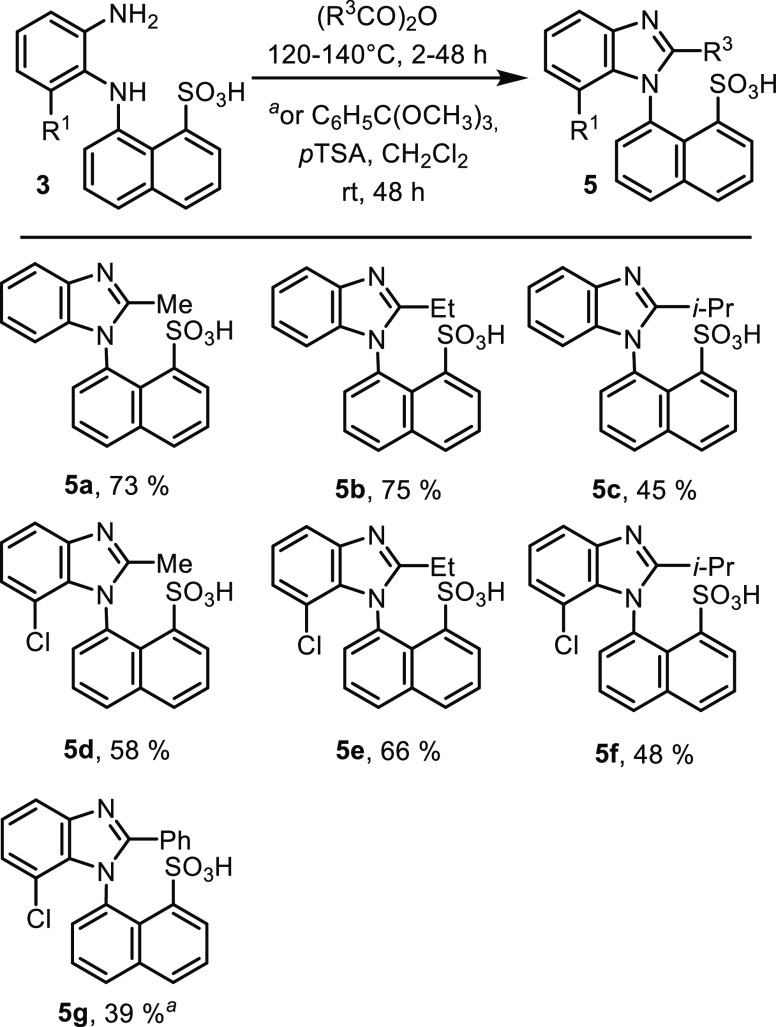
Cyclization of Phenylenediamine **3** to Benzimidazolesulfonic Acids **5**

The cyclization to benzimidazoles **5** was carried out with phenylenediamine **3** and carboxylic anhydride (Scheme 2). An alternative method, using an *ortho* ester under acidic catalysis, was employed to prepare phenyl derivative **5g**. The yields were lower after cyclization in the case where the substituents increased the steric hindrance, which led to an intended higher rotational energy barrier around the C–N bond between naphthalene and benzimidazole.

**Scheme 3:**
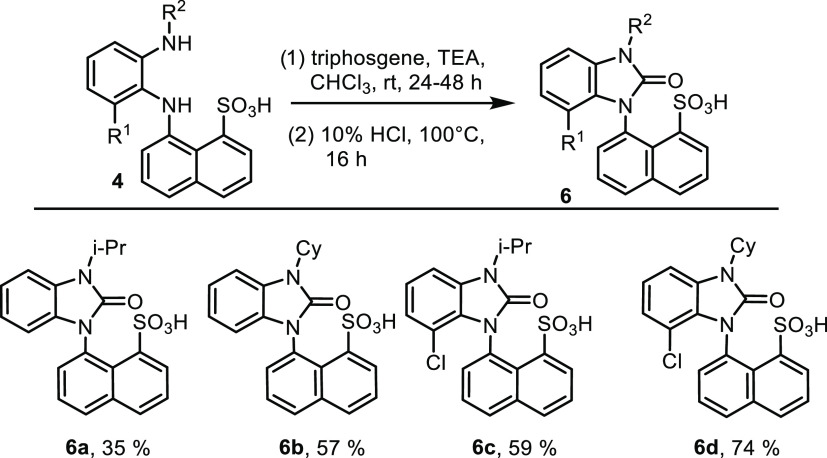
Cyclization of Phenylenediamine **4** with Triphosgene to Benzimidazolone Sulfonic Acids **6**

Triphosgene proved to be an efficient reagent for obtaining the desired benzimidazolesulfonic acids **6** (Scheme 3), despite the chlorination of the sulfonic group. However, this side reaction was later beneficial for synthesizing other functional derivatives of sulfonic acids under mild reaction conditions (Scheme 7).

**Scheme 4:**
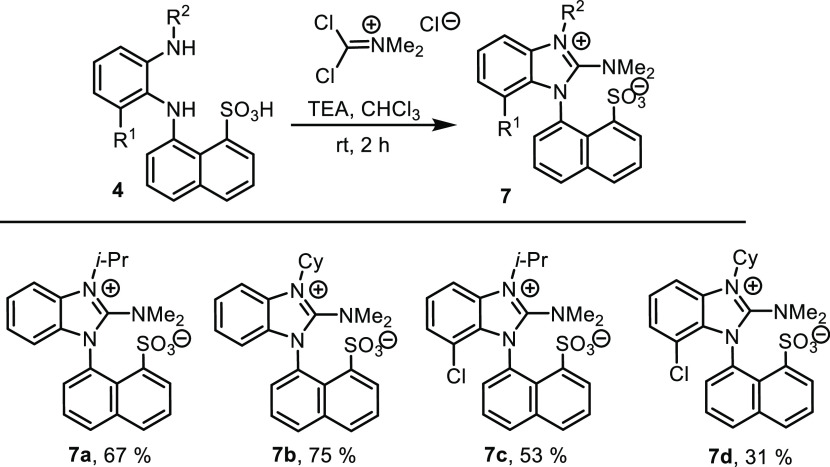
Cyclization of Phenylenediamines **4** with Phosgene Imminium Chloride to Benzimidazole Zwitterions **7**

Another type of derivative was obtained after phenylenediamine **4** was cyclized with phosgene iminium chloride, which led to novel zwitterionic structure **7** with anticipated axial chirality. The cyclization, in which R^1^ was hydrogen, proceeded very smoothly with the complete conversion. The isolation procedure afforded lower yields, but the higher lipophilicity enabled us to furnish **7b** in a good yield (Scheme 4).

**Scheme 5:**
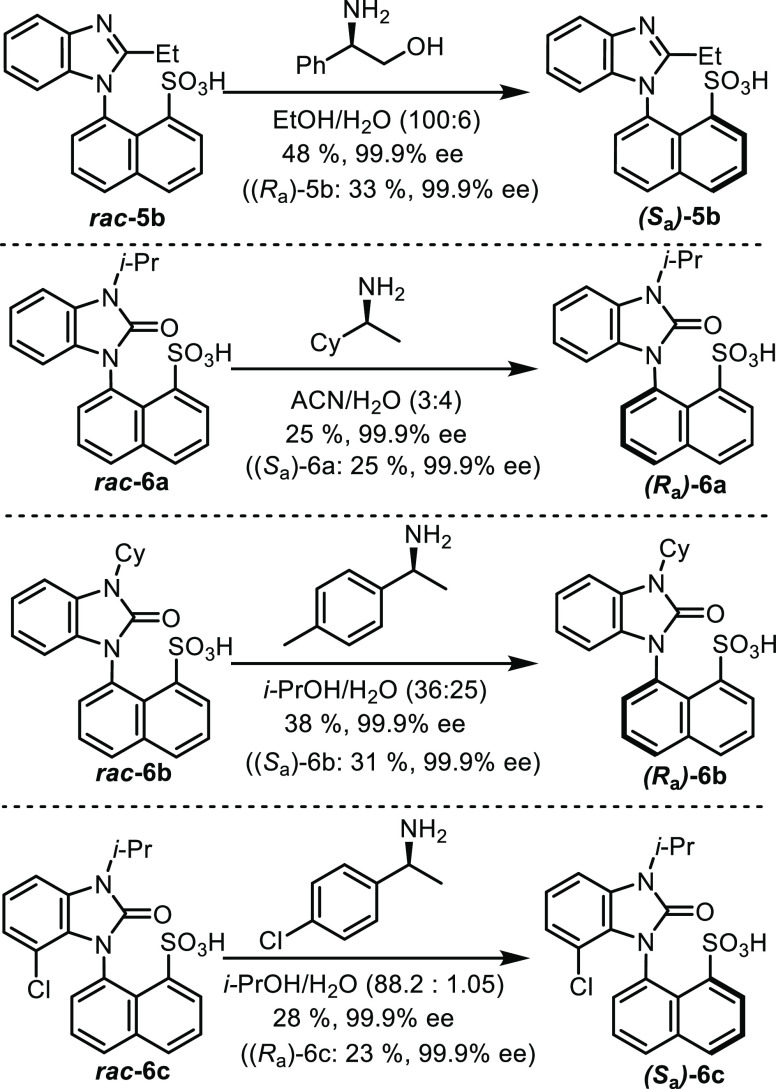
Optical Resolutions of **5b**, **6a**, **6b**, and **6c***via* Diastereomeric Salt Formation

Since we designed sulfonic acids **5** and **6** with a restricted rotation around the C–N bond to induce axial chirality, we decided to develop a simple optical resolution method *via* diastereomeric salt formation. We found very efficient crystallization conditions for four derivatives (Scheme 5). The absolute spatial arrangement of their salts was proven by single-crystal X-ray crystallography (see the Supporting Information (SI) file).

Then, we proved the stabilities of isolated atropisomers **5b**, **6a**, **6b**, and **6c** at 100 °C for 40 h (see the SI file). Sulfonic acids **5b**, **6b**, and **6c** were stable, and only **6a** was slightly prone to racemization affording 3% of the opposite atropisomer. The racemization kinetic of less stable **6a** and subsequent calculation according to Eyring equation revealed an energy barrier of 32.9 kcal (see the SI file).

**Figure 3:**
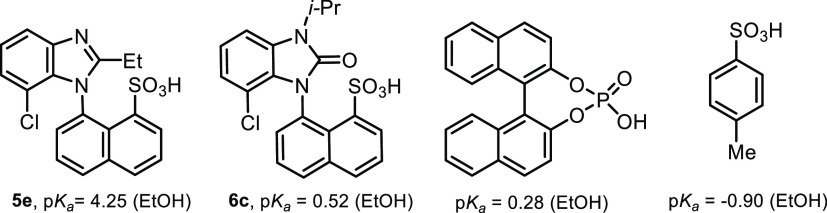
p*K*_a_ values of selected acids determined by potentiometric titration in ethanol.

Sulfonic acids **5e** and **6c** were measured in ethanol to compare their dissociation constants with *p*-toluenesulfonic acid and nonsubstituted BINOL phosphoric acid (Figure 3). Sulfonic acid **5e** with the benzimidazole ring showed a higher p*K*_a_ value, which was likely caused by the basic benzimidazole nitrogen. A possible zwitterionic form, a result of the protonation of benzimidazole nitrogen, also indicates the ^1^H NMR spectra of sulfonic acids **5** in CDCl_3_ solutions. We can see very broad signals higher than 14 ppm after magnifying the baseline of the spectra. This assumption was further strengthened by the p*K*_a_ value of benzimidazolone sulfonic acid **6c**, where the basic character of nitrogen was eliminated and we observed a lower p*K*_a_ value similar to nonsubstituted BINOL phosphoric acid. Moreover, the p*K*_a_ of compound **6c** in ethanol was calculated at the SMD/M06–2X/6–311++G(2df,2p)//M06–2X/6–31++G** level of theory using the proton-exchange method with *p*-toluenesulfonic acid as a reference. We observed very good agreement between the experimental (0.52) and calculated (0.9) p*K*_a_ values (for details see the SI file).

**Scheme 6:**
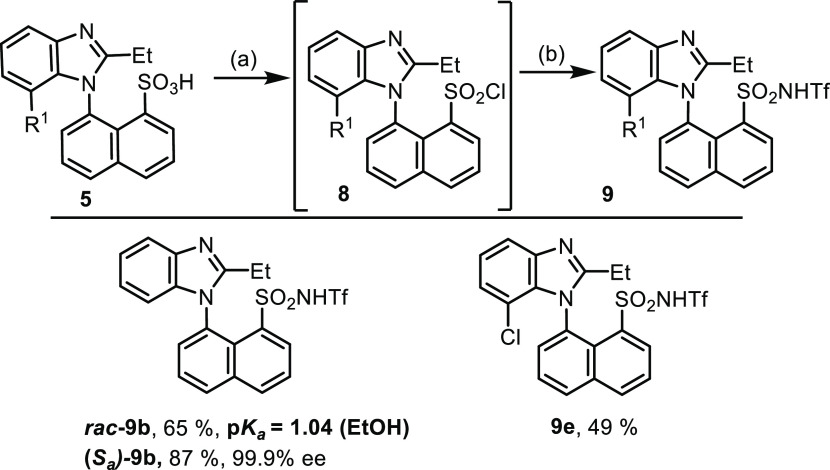
Synthesis of Triflic Imides **9** from Sulfonic Acids Reaction conditions: (a) POCl_3_, 100°C, 3 h; (b) NH_2_SO_2_CF_3_, K_2_CO_3_, ACN, rt, 16 h.

To further decrease the p*K*_a_ value, sulfonic acids were transformed into triflic imides (Scheme 6). The sulfonyl chloride functionality was accomplished by using POCl_3_ at 100 °C without isolation to generate subsequent imides **9b** and **9e** with triflic amides. Imide **9b** retained enantiopurity from sulfonic acid **5b** and its p*K*_a_ value confirmed that the acidity was substantially higher than that of sulfonic acid **5e**.

**Scheme 7:**
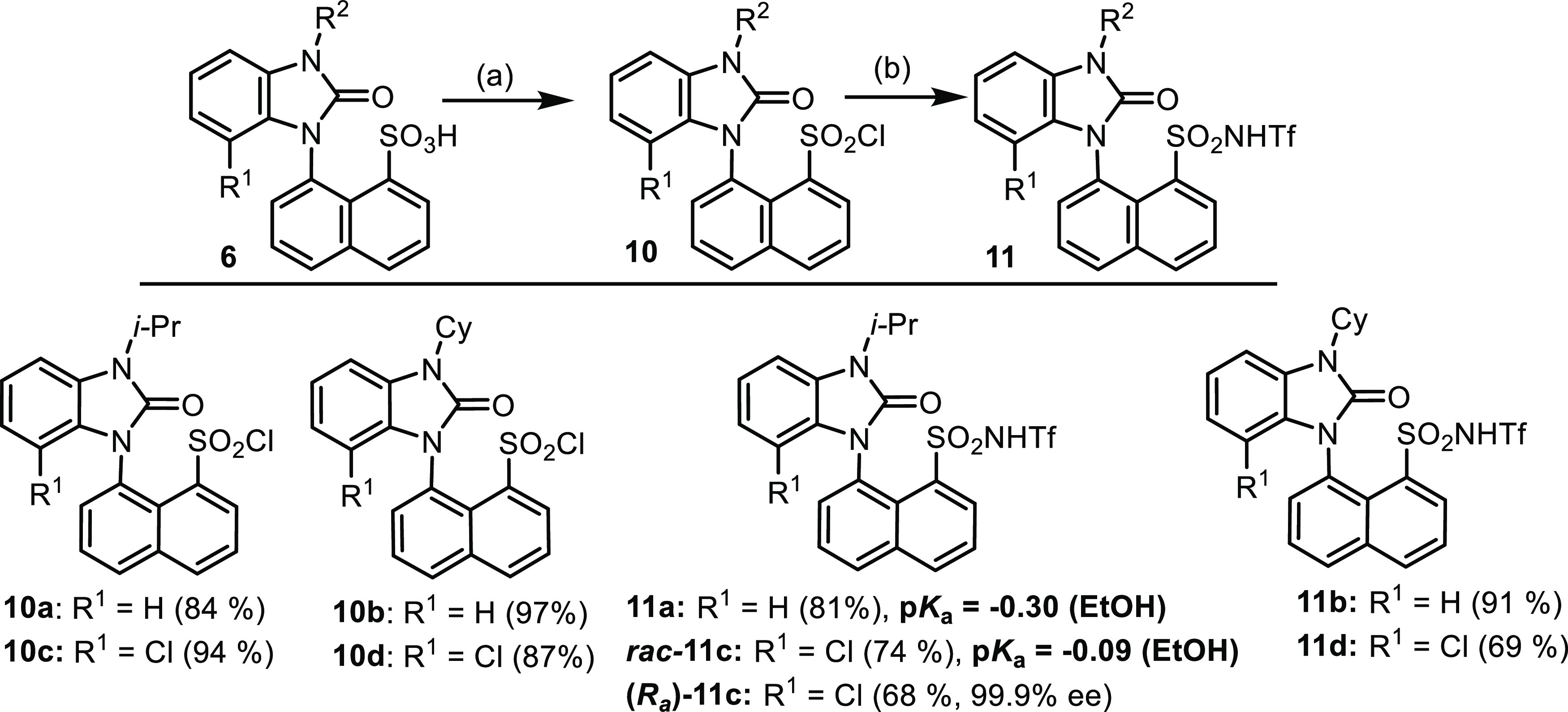
Synthesis of Triflic Imides **11** from Sulfonic Acids Reaction conditions: (a) triphosgene, triethylamine (TEA), CHCl_3_, rt, 16 h; (b) NH_2_SO_2_CF_3_, K_2_CO_3_, ACN, rt, 16 h.

However, this method was not suitable for benzimidazolones **10**, since the carbonyl group was chlorinated as well. In this case, we took a previously observed advantage of triphosgene to isolate sulfonyl chlorides 10a–d. The final step with triflic amide afforded triflic imides **11**. As expected, stronger acids were provided, which was evident by the p*K*_a_ values of **11a** and **11c**. Then, the synthesis of triflic imides was checked with enantiopure sulfonic acids **6a**–**c** to prove the stability of atropisomers. We found that racemization occurred for imides **11a** and **11b**, but **11c** was isolated as a pure atropisomer since the chlorine substituent increased the energy barrier (Scheme 7).

To demonstrate the synthesized axially chiral sulfonic acids as Brønsted acid catalysts, we decided to perform a preliminary attempt of the Pictet–Spengler reaction of tryptamine with α-angelica lactone under the catalysis of sulfonic acids **6a** and **6b** (Table 1).^24^ Tetracyclic heterocycle **14** was isolated after an enantioselective *N*-acyliminium cyclization cascade with the enantiomeric excess around 50% (entries 1–5). The amount of catalyst varied from 2.5 to 10 mol % and higher concentrations of reactants (entry 2) had the same impact on the enantioselectivity.

**Table 1:**
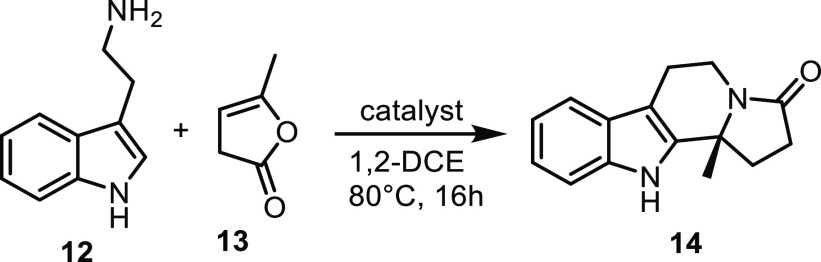
Preliminary Attempt of Pictet–Spengler Reaction of Tryptamine with α-Angelica Lactone^a^

entry	catalyst	**6** [mol %]	**14** [%]^c^	*ee* [%]^d^
1	**(*****R***_***a***_**)-6b**	5	92	–48
2^b^	**(*****S***_***a***_**)-6b**	5	94	48
3	**(*****S***_***a***_**)-6b**	2.5	90	50
4	**(*****S***_***a***_**)-6b**	10	70	54
5	**(*****S***_***a***_**)-6a**	5	67	46

a Reaction conditions: **12** (0.1 mmol), **13** (0.3 mmol), 1 mL 1,2-DCE.

b 0.5 mL 1,2-DCE.

c Isolated yields.

d Enantiomeric excess was determined by chiral SFC analysis.

## Conclusions

In summary, we developed a synthesis procedure of novel axially chiral sulfonic acids. Easily available compounds, 1-fluoro-2-nitrobenzenes and 8-aminonaphthalene-1-sulfonic acid, were used as starting materials. We prepared 11 axially chiral sulfonic acid derivatives and four of them were resolved to atropisomers by optical resolution *via* diastereomeric salt formation. Separated atropisomers were stable even at 100 °C for 40 h. Key *ortho*-phenylenediamine intermediates also enabled the synthesis of novel zwitterionic structures with anticipated axial chirality. To expand the ability to perform Brønsted acid catalysis, we also prepared triflic imide congeners. The determination of dissociation constants in ethanol was determined to range from −0.30 to 4.25 (p*K*_a_).

## Experimental Section

### General Information

Starting materials and reagents were purchased from various commercial sources (VWR, Merck, Fluorochem, Acros Organics) and used as received. All reactions were carried out under air. Reaction workup and column chromatography were performed with commercial-grade solvents without further purification. All reactions were monitored by liquid chromatography-mass spectrometry (LC/MS) analysis or by thin-layer chromatography (TLC) using aluminum plates precoated with silica gel (silica gel 60 F254, Merck) impregnated with a fluorescent indicator. TLC plates were visualized by exposure to ultraviolet light (λ = 254 nm). Column chromatography was performed using silica gel (35–70 μm particle size).

### Instrumentation

LC-MS analyses were carried out using ultrahigh-pressure liquid chromatography (UPLC) Waters Acquity equipped with PDA and QDa detectors. The system comprised XSelect HSS T3 (Waters) 3 mm × 50 mm C18 reverse phase column XP, 2.5 μm particles. Mobile phases: 10 mM ammonium acetate in HPLC-grade water (A) and gradient-grade acetonitrile for HPLC (B). A gradient was mainly formed from 20 to 80% of B in 4.5 min, kept for 1 min, with a flow rate of 0.6 mL/min. The MS ESI operated at a 25 V cone voltage, 600 °C probe temperature, and 120 °C source temperature. All ^1^H and ^13^C NMR experiments were performed at magnetic field strengths of 11.75 T (with operating frequencies of 500.16 MHz for ^1^H and 125.77 MHz for ^13^C) and 9.39 T (with operating frequencies of 399.78 MHz for ^1^H, 100.53 MHz for ^13^C, and 376.17 MHz for ^19^F) at an ambient temperature (27 °C). ^1^H and ^13^C spectra were referenced relative to the signal of DMSO-*d*_6_ (^1^H: δ = 2.50 ppm, ^13^C: δ = 39.51 ppm) or CDCl_3_ (^1^H: δ = 7.260 ppm, ^13^C: δ = 77.160 ppm). ^19^F spectra were referenced to CFCl_3_ as an internal standard (0.0 ppm). HRMS analyses were performed using UHPLC Dionex Ultimate 3000 equipped with an Orbitrap Elite high-resolution mass spectrometer, Thermo Exactive plus, operating at full scan mode (120,000 FWMH) in the range of 100–1000 *m*/*z*. The settings for electrospray ionization were as follows: oven temperature of 150 °C and a source voltage of 3.6 kV. The acquired data were internally calibrated with diisooctyl phthalate as a contaminant in MeOH (*m*/*z* 391.2843). SFC chiral analyses were performed using an Acquity UPC^2^ system (Waters, MA) equipped with PDA 2998, and QDa detectors. All chromatographic separations were carried out using chiral analytical column Chiralpak ID-3 (4.6 mm × 100 mm, 3 μm particle size), at a flow rate of 2.2 mL/min, column temperature of 38 °C, and ABPR 2000 psi. A composition of the mobile phase was adjusted according to the analyzed compound. Melting points were determined on a VEB Analytik Dresden PHMK 78/1586 apparatus. Potentiometric S4 titration for the determination of the dissociation constant was carried out using a benchtop meter pH 50+ DHS (Instruments XS, Italy) equipped with the glass electrode ScienceLine N 6480 eth (SI AnalyticsTM, Germany), electrolyte solution L 5034—LiCl in ethanol (SI Analytics, Germany). Before each measurement, the pH meter was calibrated with buffer solutions of pH 4.01 and 7.00. The titration was performed using Titronic basic piston burette (Schott Instruments, Germany). 0.05 and 0.005 M basic solutions in EtOH were prepared by dissolving an appropriate amount of KOH (Penta Chemicals) in absolute ethanol (>99,7%, VWR Chemicals). The titrant was added in increments of 0.02–0.1 mL. Dissociation constants were calculated from titration curves using the programe OriginePro 9.

#### 8-((2-Nitrophenyl)amino)naphthalene-1-sulfonic Acid **2a**

1-Fluoro-2-nitrobenzene **1a** (5.22 mL, 49.5 mmol) was dissolved in DMSO (50 mL). 8-Aminonaphthalene-1-sulfonic acid (11.04 g, 49.5 mmol) and K_2_CO_3_ (13.6 g, 99.0 mmol) were added and the resulting mixture was heated at 130 °C for 12 h. After cooling to rt, the mixture was slowly diluted with an aqueous solution of 10% HCl (700 mL). Activated charcoal was added and the suspension was stirred for 10 min at rt, then it was filtered through a pad of celite and washed with distilled water. The filtrate was extracted with EtOAc (3 × 300 mL) and combined extracts were dried over MgSO_4_ and concentrated on RVO. Compound **2a** was isolated as a red amorphous solid to yield 12.14 g (71%). ^1^H NMR (400 MHz, DMSO-*d*_6_): δ 11.20 (br. s., 1H), 8.22 (dd, *J* = 7.3, 1.5 Hz, 1H), 8.02 (dd, *J* = 8.5, 1.6 Hz, 1H), 8.01–7.97 (m, 1H), 7.85 (dd, *J* = 7.3, 2.2 Hz, 1H), 7.56–7.49 (m, 2H), 7.47 (dd, *J* = 7.3, 0.8 Hz, 1H), 7.28–7.22 (m, 1H) 6.73 (dd, *J* = 8.6, 1.1 Hz, 1H) 6.71–6.66 (m, 1H), 6.42 (br. s., 1H). ^13^C{^1^H} NMR (101 MHz, DMSO-*d*_6_): δ 144.9, 142.7, 136.5, 136.0, 134.7, 134.5, 131.2, 127.5, 126.8, 126.2, 125.8, 125.6, 124.6, 117.2, 116.3. HRMS (ESI) *m*/*z* calculated for C_16_H_12_N_2_O_5_S [M – H]^−^ 343.0383, found 343.0392.

#### 8-((2-Chloro-6-nitrophenyl)amino)naphthalene-1-sulfonic Acid **2b**

1-Chloro-2-fluoro-3-nitrobenzene **1b** (2.93 mL, 25.0 mmol) was dissolved in DMSO (50 mL). 8-Aminonaphthalene-1-sulfonic acid (5.58 g, 25.0 mmol) and K_2_CO_3_ (6.9 g, 50.0 mmol) were added and the resulting mixture was heated at 130 °C for 18 h. After cooling to rt, the mixture was slowly diluted with an aqueous solution of 10% HCl (350 mL). Activated charcoal was added and the suspension was stirred for 10 min at rt, then it was filtered through the pad of celite and washed with distilled water. The filtrate was extracted with EtOAc (3 × 200 mL) and combined extracts were dried over MgSO_4_ and concentrated on RVO. Compound **2b** was isolated as a red amorphous solid to yield 5.56 g (59%). ^1^H NMR (500 MHz, DMSO-*d*_6_): δ 11.11 (s, 1H), 8.23 (dd, *J* = 7.3, 1.4 Hz, 1H), 7.86 (dd, *J* = 8.2, 1.2 Hz, 1H), 7.79 (dd, *J* = 8.3, 1.5 Hz, 1H), 7.71 (dd, *J* = 7.9, 1.5 Hz, 1H), 7.46 (dd, *J* = 8.1, 1.1 Hz, 1H), 7.42–7.35 (m, 1H), 7.20 (t, *J* = 7.8 Hz, 1H), 7.05 (t, *J* = 8.1 Hz, 1H), 6.66 (dd, *J* = 7.6, 1.2 Hz, 1H). ^13^C{^1^H} NMR (101 MHz, DMSO-*d*_6_): δ 143.0, 142.5, 139.1, 136.3, 135.8, 134.9, 131.2, 128.6, 127.0, 125.5, 124.6, 124.3, 122.9, 122.3, 120.8, 114.3. HRMS (ESI) *m*/*z* calculated for C_16_H_11_ClN_2_O_5_S [M – H]^−^ 379.0150, found 379.0153.

#### 8-((2-Aminophenyl)amino)naphthalene-1-sulfonic Acid **3a**

8-((2-Nitrophenyl)amino)naphthalene-1-sulfonic acid **2a** (3.44 g, 10.0 mmol) was dissolved in MeOH (150 mL). The solution was degassed with the flow of nitrogen, then 10 mol % of 10% wt Pd on activated charcoal (1.06 g) was added. The mixture was degassed again and hydrogen was introduced. After 2 h, the resulting suspension was filtered through the pad of celite and washed with MeOH. The filtrate was evaporated on RVO. The residue was dissolved in DCM (5 mL) and precipitated by the addition of hexane. Product **3a** was isolated by filtration as a light brown solid to yield 2.5 g (80%). Melting point: 216–220 °C. ^1^H NMR (400 MHz, DMSO-*d*_6_): δ 10.04 (br. s., 2H), 8.24 (dd, *J* = 7.3, 1.4 Hz, 1H), 7.95 (dd, *J* = 8.3, 1.3 Hz, 1H), 7.51 (dd, *J* = 8.1, 1.1 Hz, 1H), 7.48–7.43 (m, 1H), 7.40 (t, *J* = 7.8 Hz, 1H), 7.37–7.31 (m, 2H), 7.29 (td, *J* = 8.2, 7.7, 1.4 Hz, 1H), 7.13–7.08 (m, 1H), 6.96 (dd, *J* = 7.6, 1.3 Hz, 1H). ^13^C{^1^H} NMR (101 MHz, DMSO-*d*_6_): δ 141.7, 139.6, 137.3, 136.6, 131.9, 128.6, 127.2, 126.1, 125.1, 124.4, 124.1, 122.8, 121.9, 121.6, 115.7. HRMS (ESI) *m*/*z* calculated for C_16_H_13_N_2_O_3_S [M – H]^−^ 313.0641, found 313.0650.

#### 8-((2-Amino-6-chlorophenyl)amino)naphthalene-1-sulfonic Acid **3b**

8-((2-Chloro-6-nitrophenyl)amino)naphthalene-1-sulfonic acid **2b** (5.23 g, 16.0 mmol) was dissolved in 150 mL of MeOH. The solution was degassed with the flow of nitrogen, then 10 mol % of 10% wt. Pd on activated charcoal (1.70 g) was added. The mixture was degassed again and hydrogen was introduced. After 2 h, the resulting suspension was filtered through the pad of celite and washed with MeOH. The filtrate was evaporated and the residue was suspended in a small amount of MeOH. Product **3b** was isolated by filtration as a brown solid to yield 2.33 g (42%). Melting point: 252–254 °C. ^1^H NMR (400 MHz, DMSO-*d*_6_): δ 10.00 (s, 1H), 8.18 (dd, *J* = 7.3, 1.4 Hz, 1H), 7.88 (dd, *J* = 8.3, 1.3 Hz, 1H), 7.44–7.39 (m, 1H), 7.37–7.30 (m, 2H), 7.28 (dd, *J* = 8.0, 1.3 Hz, 1H), 7.22 (t, *J* = 7.7 Hz, 1H), 7.19 (dd, *J* = 7.3, 2.1 Hz, 1H), 6.13 (dd, *J* = 7.6, 1.4 Hz, 1H). ^13^C{^1^H} NMR (101 MHz, DMSO-*d*_6_): δ 142.9, 141.3, 137.4, 136.5, 135.4, 131.6, 129.9, 128.57, 126.6, 126.1, 125.8, 124.2, 120.8, 119.4, 118.7, 108.6. HRMS (ESI) *m*/*z* calculated for C_16_H_12_ClN_2_O_3_S [M – H]^−^ 347.0252, found 347.0261.

#### 8-((2-(Isopropylamino)phenyl)amino)naphthalene-1-sulfonic Acid **4a**

8-((2-Aminophenyl)amino)naphthalene-1-sulfonic acid **3a** (2.34 g, 7.54 mmol) was suspended in a mixture of acetone/MeOH (1:1, 30 mL). NaBH(OAc)_3_ (4.72 g, 22.6 mmol) was slowly added and the reaction mixture was allowed to stir at rt for 22 h. After the reaction was completed, the mixture was concentrated under reduced pressure, suspended in MeOH (5 mL), and poured into cold water (55 mL). The precipitated solid was filtered off and purified by column chromatography (DCM/MeOH, 10:1) to yield 1.71 g (64%) of **4a** as a white solid. Melting point: 210–212 °C. ^1^H NMR (400 MHz, DMSO-*d*_6_): δ 10.08 (s, 1H), 8.23 (dd, *J* = 7.3, 1.4 Hz, 1H), 7.93 (dd, *J* = 8.2, 1.2 Hz, 1H), 7.50–7.36 (m, 5H), 7.34 (t, *J* = 7.8 Hz, 1H), 7.31–7.23 (m, 1H), 6.85 (d, *J* = 7.5 Hz, 1H), 3.84 (hept, *J* = 6.3 Hz, 1H), 1.20 (d, *J* = 6.5 Hz, 6H). ^13^C{^1^H} NMR (101 MHz, DMSO-*d*_6_): δ 142.1, 141.1, 137.4, 137.1, 132.4, 130.5, 129.8, 127.4, 126.7, 126.6, 125.3, 125.0, 124.9, 121.5, 121.2, 113.5, 54.0, 19.4. HRMS (ESI) *m*/*z* calculated for C_19_H_19_N_2_O_3_S [M + H]^+^ 357.1267, found 357.1267.

#### 8-((2-(Cyclohexylamino)phenyl)amino)naphthalene-1-sulfonic Acid **4b**

8-((2-Aminophenyl)amino)naphthalene-1-sulfonic acid **3a** (2.0 g, 6.37 mmol) was suspended in MeOH (25 mL). Cyclohexanone (6.6 mL, 63.7 mmol) was added, followed by slow addition of NaBH(OAc)_3_ (4.05 g, 19.11 mmol), and the reaction mixture was allowed to stir at rt for 90 min. After the reaction was completed, the resulting mixture was poured into cold water (50 mL). The precipitate was filtered off to yield 2.4 g (96%) of **4b** as a light pink solid. Melting point: 172–176 °C. ^1^H NMR (400 MHz, DMSO-*d*_6_): δ 9.99 (s, 1H), 8.23 (dd, *J* = 7.3, 1.4 Hz, 1H), 7.93 (dd, *J* = 8.3, 1.2 Hz, 1H), 7.52–7.31 (m, 6H), 7.22 (dd, *J* = 7.5, 1.2 Hz, 1H), 6.87 (d, *J* = 7.4 Hz, 1H), 3.61–3.47 (m, 3H), 1.92–1.85 (m, 2H), 1.72–1.62 (m, 2H), 1.54–1.46 (m, 1H), 1.36–1.21 (qd, *J* = 12.3, 2.9 Hz, 2H), 1.12 (qt, *J* = 12.8, 3.0 Hz, 2H), 1.00 (tt, *J* = 12.6, 3.1 Hz, 1H). ^13^C{^1^H} NMR (101 MHz, DMSO-*d*_6_): δ 142.2, 141.1, 137.4, 137.1, 132.4, 130.0, 129.3, 127.4, 126.6, 126.1, 124.9, 124.8, 124.6, 121.8, 121.3, 114.1, 59.8, 29.6, 25.2, 24.5. HRMS (ESI) *m*/*z* calculated for C_22_H_23_N_2_O_3_S [M – H]^−^ 395.1424, found 395.1435.

#### 8-((2-Chloro-6-(isopropylamino)phenyl)amino)naphthalene-1-sulfonic Acid **4c**

Sulfonic acid **3b** (8.15 g, 23.42 mmol) was dissolved in a mixture of acetone/MeOH (1:1, 100 mL). NaBH(OAc)_3_ (14.9 g, 70.26 mmol) was added and the reaction mixture was stirred at rt for 20 h. After that, the additional portion of NaBH(OAc)_3_ (14.9 g, 70.26 mmol) was added and the mixture was allowed to stir at rt for another 20 h. After the reaction was completed, the resulting mixture was concentrated under vacuum and water (180 mL) was added to the residue. The precipitate was filtered off and purified by column chromatography with DCM/MeOH (100:1–10:1) to yield 3.84 g (42%) of **4c** as a white solid. Melting point: 194–196 °C. ^1^H NMR (500 MHz, DMSO-*d*_*6*_): δ 10.05 (s, 1H), 8.14 (dd, *J* = 7.2, 1.2 Hz, 1H), 7.85 (dd, *J* = 8.4, 1.1 Hz, 1H), 7.40 (t, *J* = 7.7 Hz, 1H), 7.36 (t, *J* = 8.1 Hz, 1H), 7.28–7.09 (m, 5H), 6.11 (dd, *J* = 7.5, 1.2 Hz, 1H), 3.60 (hept, *J* = 6.4 Hz, 1H), 1.14 (d, *J* = 6.3 Hz, 3H), 0.97 (d, *J* = 6.4 Hz, 3H). ^13^C{^1^H} NMR (101 MHz, DMSO-*d*_6_): δ 142.1, 142.0, 141.3, 136.4, 135.5, 131.4, 129.0, 127.8, 126.3, 125.9, 124.0, 122.9, 119.1, 117.9, 116.8, 107.4, 48.9, 20.7, 20.0. HRMS (ESI) *m*/*z* calculated for C_19_H_18_ClN_2_O_3_S [M – H]^−^ 389.0721, found 389.0732.

#### 8-((2-Chloro-6-(cyclohexylamino)phenyl)amino)naphthalene-1-sulfonic Acid **4d**

Sulfonic acid **3b** (1.5 g, 4.31 mmol) was suspended in MeOH (17 mL). Cyclohexanone (4.47 mL, 43.1 mmol) was added, followed by slow addition of NaBH(OAc)_3_ (2.74 g, 12.72 mmol). The reaction mixture was allowed to stir at rt for 75 min. After the reaction was completed, the resulting mixture was poured into cold water (50 mL). The precipitated light pink solid was filtered off to yield 1.67 g (90%) of **4d**. Melting point: 192–194 °C. ^1^H NMR (400 MHz, DMSO-*d*_6_): δ 10.04 (s, 1H), 8.17 (dd, *J* = 7.3, 1.5 Hz, 1H), 8.14 (br. s., 1H), 7.86 (dd, *J* = 8.5, 1.5 Hz, 1H), 7.40 (dd, *J* = 8.1, 7.3 Hz, 1H), 7.33 (t, *J* = 8.1 Hz, 1H), 7.24 (dd, *J* = 8.1, 1.5 Hz, 1H), 7.22–7.08 (m, 3H), 6.12 (dd, *J* = 9.0, 1.6 Hz, 1H), 3.29–3.19 (m, 1H), 1.92–1.82 (m, 1H), 1.70–1.55 (m, 2H), 1.54–1.40 (m, 2H), 1.32–1.19 (m, 1H), 1.19–1.09 (m, 1H), 1.09–0.90 (m, 3H). ^13^C{^1^H} NMR (101 MHz, DMSO-*d*_6_): δ 142.2, 142.0, 141.4, 136.4, 135.4, 131.4, 128.7, 127.5, 126.3, 125.8, 124.0, 122.2, 119.2, 117.9, 116.0, 107.5, 55.3, 30.8, 30.1, 24.9, 24.2. HRMS (ESI) *m*/*z* calculated for C_22_H_22_ClN_2_O_3_S [M – H]^−^ 429.1034, found 429.1044.

#### 8-(2-Methyl-1*H*-benzo[*d*]imidazol-1-yl)naphthalene-1-sulfonic Acid **5a**

Acetic anhydride (3 mL) was added to diamine **3a** (0.94 g, 3.0 mmol). The reaction mixture was stirred at 120 °C for 18 h. After the reaction was completed, it was cooled to rt and the formed precipitate was filtered off and washed with MeOH. The crude product was precipitated again in DMSO (6 mL), filtered off, and washed with MeOH. Then, it was stirred in a mixture of MeOH/10% aqueous HCl (1:2, 9.5 mL) for 1 h. Compound **5a** was isolated as a white solid by filtration to yield 0.74 g (73%). Melting point: >330 °C. ^1^H NMR (500 MHz, DMSO-*d*_6_): δ 8.34 (dd, *J* = 7.3, 1.4 Hz, 1H), 8.14 (dd, *J* = 8.3, 1.3 Hz, 1H), 8.09 (dd, *J* = 8.3, 1.3 Hz, 1H), 7.62–7.58 (m, 1H), 7.58–7.54 (m, 1H), 7.47–7.40 (m, 1H), 7.18 (dd, *J* = 7.3, 1.4 Hz, 1H), 7.05–6.99 (m, 1H), 6.96–6.91 (m, 1H), 6.72 (dt, *J* = 7.9, 0.9 Hz, 1H), 2.10 (s, 3H). ^13^C{^1^H} NMR (126 MHz, DMSO-*d*_6_): δ 154.7, 143.8, 142.7, 140.0, 135.7, 133.4, 130.7, 130.4, 129.8, 129.3, 127.4, 125.6, 125.1, 120.5, 119.9, 117.2, 111.4, 14.8. HRMS (ESI) *m*/*z* calculated for C_18_H_13_N_2_O_3_S [M – H]^−^ 337.0641, found 337.0652.

#### 8-(2-Ethyl-1*H*-benzo[*d*]imidazol-1-yl)naphthalene-1-sulfonic Acid **5b**

Propionic anhydride (12 mL) was added to diamine **3a** (2.5 g, 8.0 mmol). The reaction mixture was stirred at 120 °C for 2 h. After the reaction was completed, it was cooled to rt and the formed precipitate was filtered off and washed with MeOH. Sulfonic acid **5b** was isolated as a white solid after crystallization from the mixture of MeOH/CHCl_3_ (5:1, 60 mL) to yield 2 g (75%). Melting point: >330 °C. ^1^H NMR (500 MHz, DMSO-*d*_6_): δ 14.46 (s, 1H), 8.40–8.33 (m, 2H), 8.21 (dd, *J* = 8.2, 1.4 Hz, 1H), 7.78–7.73 (m, 3H), 7.64 (t, *J* = 8.0 Hz, 1H), 7.50–7.43 (m, 1H), 7.39–7.32 (m, 1H), 7.03 (d, *J* = 8.3 Hz, 1H), 2.98–2.82 (m, 1H), 2.65–2.53 (m, 1H), 1.21 (t, *J* = 7.5 Hz, 3H). ^13^C{^1^H} NMR (126 MHz, DMSO-*d*_6_): δ 158.1, 142.4, 136.4, 135.9, 133.0, 131.4, 130.7, 130.0, 129.5, 128.0, 125.9, 125.7, 124.9, 124.6, 113.6, 113.1, 21.0, 9.7. HRMS (ESI) *m*/*z* calculated for C_19_H_15_N_2_O_3_S [M – H]^−^ 351.0798, found 351.0808.

#### Optical Resolution of (*R*_a_)-8-(2-Ethyl-1*H*-benzo[*d*]imidazol-1-yl)naphthalene-1-sulfonic Acid **5b**

Racemic sulfonic acid **5b** (6.6 g, 18.8 mmol) was transferred into a 500 mL round bottom flask. EtOH (200 mL) and (*S*)-(+)-phenylglycinol (2.58 g, 18.8 mmol) were added. The mixture was heated at 65 °C, and then water was added (12 mL). After stirring at 65 °C for 30 min, a solution was formed and after another 2 h at rt, the solution was concentrated under reduced pressure to 2/3 of the total volume. At this point, crystallization began and the mixture was stirred at rt for 20 h. A diastereomeric salt of **5b** was collected by filtration and washed with a small amount of water. Then, it was acidified with 10% aqueous HCl (35 mL), stirred for 2 h, and filtered off. Sulfonic acid **(*****R***_**a**_**)-5b** was isolated as a white solid to yield 2.2 g (33%, 99.9% ee), **[α]**_**D**_^**22**^ −144.79° (*c* 0.90, CHCl_3_/MeOH 1:1). The procedure was also used with (*R*)-(−)-phenylglycinol (1.33 g, 9.7 mmol) and ***rac*****-5b** (3.41 g, 9.7 mmol) to yield **(*****S***_**a**_**)-5b** (1.65 g, 48%, 99.9% ee), **[α]**_**D**_^**22**^ +144.61° (*c* 0.90, CHCl_3_/MeOH 1:1).

#### 8-(2-Isopropyl-1*H*-benzo[*d*]imidazol-1-yl)naphthalene-1-sulfonic Acid **5c**

Isobutyric andhydride (3 mL) was added to diamine **3a** (0.942 g, 3 mmol). The reaction mixture was stirred at 160 °C for 2 h. After the reaction was completed, it was cooled to rt and the formed precipitate was filtered off and washed with MeOH. Sulfonic acid **5c** was isolated as a light pink solid after crystallization from a mixture of MeOH/CHCl_3_ (25:8, 32 mL) to yield 0.495 g (45%). Melting point: >330°C. ^1^H NMR (500 MHz, DMSO-*d*_6_): δ 14.36 (s, 1H), 8.37 (dt, *J* = 7.4, 1.7 Hz, 2H), 8.21 (dd, *J* = 8.3, 1.2 Hz, 1H), 7.83–7.72 (m, 3H), 7.67–7.63 (m, 1H), 7.53–7.45 (m, 1H), 7.42–7.37 (m, 1H), 7.13 (dt, *J* = 8.3, 0.8 Hz, 1H), 2.83 (p, *J* = 6.9 Hz, 1H), 1.46 (d, *J* = 6.8 Hz, 3H), 0.92 (d, *J* = 7.1 Hz, 3H). ^13^C{^1^H} NMR (126 MHz, DMSO-*d*_6_): δ 160.7, 142.3, 136.2, 135.9, 133.1, 131.4, 131.1, 130.0, 129.4, 127.9, 125.9, 125.7, 125.6, 125.0, 124.7, 114.1, 113.1, 27.5, 20.6, 18.4. HRMS (ESI) *m*/*z* calculated for C_20_H_17_N_2_O_3_S [M – H]^−^ 365.0954, found 365.0963.

#### 8-(7-Chloro-2-methyl-1*H*-benzo[*d*]imidazol-1-yl)naphthalene-1-sulfonic Acid **5d**

Acetic anhydride (0.75 mL) was added to diamine **3b** (0.26 g, 0.75 mmol). The reaction mixture was stirred at 120 °C for 18 h. After the reaction was completed, it was cooled to rt and the formed precipitate was filtered off and washed with MeOH. The crude product was dissolved in a mixture of MeOH/5% aqueous solution of NaOH at 70 °C and then acidified with 35% aqueous HCl to pH = 1. Sulfonic acid **5d** was isolated by filtration as a light brown solid after cooling to rt to yield 0.162 g (58%). Melting point: 240 °C. ^1^H NMR (400 MHz, DMSO-*d*_6_): δ 8.40 (dd, *J* = 7.4, 1.5 Hz, 1H), 8.33 (dd, *J* = 7.9, 1.8 Hz, 1H), 8.18 (dd, *J* = 8.4, 1.4 Hz, 1H), 7.77–7.68 (m, 3H), 7.64–7.59 (m, 1H), 7.40 (t, *J* = 8.0 Hz, 1H), 7.33 (dd, *J* = 7.9, 0.9 Hz, 1H), 2.43 (s, 3H). ^13^C{^1^H} NMR (101 MHz, DMSO-*d*_6_): δ 156.3, 144.6, 143.7, 135.8, 135.6, 133.0, 131.1, 130.9, 129.3, 128.2, 125.0, 124.9, 121.9, 120.6, 116.5, 116.5, 116.4, 15.2. HRMS (ESI) *m*/*z* calculated for C_18_H_12_ClN_2_O_3_S [M – H]^−^ 371.0252, found 371.0261.

#### 8-(7-Chloro-2-ethyl-1*H*-benzo[*d*]imidazol-1-yl)naphthalene-1-sulfonic Acid **5e**

Propionic anhydride (1.0 mL) was added to diamine **3b** (0.60 g, 1.73 mmol). The reaction mixture was stirred at 120 °C for 16 h. After the reaction was completed, it was cooled to rt and the formed precipitate was filtered off and washed with MeOH. Sulfonic acid **5e** was isolated as a light solid after purification by column chromatography (DCM/MeOH, 10:1) to yield 0.44 g (66%). Melting point: 254–258 °C. ^1^H NMR (400 MHz, DMSO-*d*_6_): δ 8.39 (dd, *J* = 7.4, 1.4 Hz, 1H), 8.33 (dd, *J* = 8.2, 1.5 Hz, 1H), 8.18 (dd, *J* = 8.4, 1.4 Hz, 1H), 7.78 (d, *J* = 1.5 Hz, 1H), 7.73 (dd, *J* = 8.1, 1.1 Hz, 1H), 7.70 (t, *J* = 7.4 Hz, 1H), 7.62 (dd, *J* = 8.0, 7.5 Hz, 1H), 7.42 (t, *J* = 8.0 Hz, 1H), 7.35 (dd, *J* = 7.9, 1.0 Hz, 1H), 2.90–2.80 (m, 1H), 2.62–2.52 (m, 1H), 1.25 (t, *J* = 7.5 Hz, 3H). ^13^C{^1^H} NMR (101 MHz, DMSO-*d*_6_): δ 159.6, 142.3, 135.6, 133.3, 132.2, 131.6, 131.5, 131.5, 129.9, 128.6, 126.5, 126.1, 125.7, 125.3, 125.1, 118.8, 112.5, 21.3, 9.7. HRMS (ESI) *m*/*z* calculated for C_19_H_14_ClN_2_O_3_S [M – H]^−^ 385.0408, found 385.0416.

#### 8-(7-Chloro-2-isopropyl-1*H*-benzo[*d*]imidazol-1-yl)naphthalene-1-sulfonic Acid **5f**

Isobutyric andhydride (1 mL) was added to diamine **3b** (0.174 g, 0.5 mmol). The reaction mixture was stirred at 140 °C for 48 h. After the reaction was completed, it was cooled to rt and the formed precipitate was filtered off and washed with MeOH. Sulfonic acid **5f** was isolated as a beige solid after purification by column chromatography (DCM/MeOH, 7:1) to yield 0.096 g (48%). Melting point: 305–310 °C. ^1^H NMR (400 MHz, DMSO-*d*_6_): δ 8.41 (dd, *J* = 7.4, 1.4 Hz, 1H), 8.35 (dd, *J* = 8.3, 1.3 Hz, 1H), 8.19 (dd, *J* = 8.3, 1.3 Hz, 1H), 7.88 (dd, *J* = 7.4, 1.4 Hz, 1H), 7.75 (dd, *J* = 8.0, 1.1 Hz, 1H), 7.75–7.70 (m, 1H), 7.66–7.59 (m, 1H), 7.46 (t, *J* = 8.0 Hz, 1H), 7.40 (dd, *J* = 7.9, 1.1 Hz, 1H), 2.79 (hept, *J* = 6.9 Hz, 1H), 1.47 (d, *J* = 6.8 Hz, 3H), 1.03 (d, *J* = 7.1 Hz, 3H). ^13^C{^1^H} NMR (101 MHz, DMSO-*d*_6_): δ 162.2, 142.2, 135.6, 133.4, 132.2, 132.0, 131.6, 131.5, 129.9, 128.2, 126.3, 126.3, 125.7, 125.4, 124.9, 119.3, 112.5, 27.6, 21.0, 18.6. HRMS (ESI) *m*/*z* calculated for C_20_H_16_ClN_2_O_3_S [M – H]^−^ 399.0565, found 399.0574.

#### 8-(7-Chloro-2-phenyl-1*H*-benzo[*d*]imidazol-1-yl)naphthalene-1-sulfonic Acid **5g**

Diamine **3b** (0.174 g, 0.5 mmol) was dissolved in DCM (5 mL). Trimethyl orthobenzoate (0.44 mL, 2.5 mmol) and *p*-toluenesulfonic acid (0.048 g, 0.25 mmol) were subsequently added. The reaction mixture was allowed to stir at rt for 48 h. After the reaction was completed, the resulting mixture was concentrated under vacuum. The residue was dissolved in MeOH (5 mL), and then water (20 mL) was added. The precipitate was filtered off and purified by column chromatography (DCM/MeOH, 10:1) to yield 85 mg (39%) of sulfonic acid **5g** as a white solid. Melting point: >330°C. ^1^H NMR (400 MHz, DMSO-*d*_6_): δ 8.44 (d, *J* = 7.0 Hz, 1H), 8.26 (d, *J* = 7.9 Hz, 1H), 8.14 (d, *J* = 7.9 Hz, 1H), 7.82 (d, *J* = 8.0 Hz, 1H), 7.77 (d, *J* = 6.9 Hz, 1H), 7.63–7.52 (m, 4H), 7.50 (t, *J* = 8.0 Hz, 1H), 7.46–7.39 (m, 2H), 7.28 (t, *J* = 7.7 Hz, 2H). ^13^C{^1^H} NMR (101 MHz, DMSO-*d*_6_): δ 154.7, 143.2, 138.9, 135.4, 135.3, 132.1, 132.0, 131.7, 131.2, 130.5, 130.0, 129.9, 128.7, 127.8, 127.5, 125.3, 124.8, 124.4, 123.2, 118.6, 115.4. HRMS (ESI) *m*/*z* calculated for C_23_H_14_ClN_2_O_3_S [M – H]^−^ 433.0408, found 433.0417.

#### 8-(3-Isopropyl-2-oxo-2,3-dihydro-1*H*-benzo[*d*]imidazol-1-yl)naphthalene-1-sulfonic Acid **6a**

Diamine **4a** (8.0 g, 22.47 mmol) was dissolved in CHCl_3_ (360 mL). TEA (9.4 mL, 67.4 mmol) and triphosgene (6.65 g, 22.5 mmol) were subsequently added and stirred at rt for 48 h, then washed with distilled water (3 × 180 mL), dried over MgSO_4_, and evaporated under vacuum. To the residue, 10% HCl (40 mL) was added and the mixture was heated at 100 °C for 16 h. The reaction mixture was allowed to cool to rt and diluted with 10% aqueous HCl (160 mL). The crude product was extracted with EtOAc (3 × 200 mL). Combined extracts were dried over MgSO_4_ and concentrated under vacuum. The residue was purified by column chromatography (DCM/MeOH, 10:1). The product after purification was dissolved in methanolic HCl (120 g/L, 20 mL) and dried using a flow of nitrogen and vacuum to yield 3.1 g of **6a** as a white solid (35%). Melting point: 151 °C. ^1^H NMR (400 MHz, DMSO-*d*_6_): δ 8.22 (dd, *J* = 7.2, 1.2 Hz, 1H), 8.04 (dd, *J* = 8.2, 1.2 Hz, 1H), 8.01 (dd, *J* = 8.2, 1.0 Hz, 1H), 7.60–7.55 (m, 1H), 7.52–7.47 (m, 1H), 7.36 (dd, *J* = 7.3, 1.3 Hz, 1H), 7.19 (d, *J* = 7.6 Hz, 1H), 6.94 (td, *J* = 7.6, 1.2 Hz, 1H), 6.88 (td, *J* = 7.6, 1.0 Hz, 1H), 6.75 (dd, *J* = 7.7, 1.0 Hz, 1H), 4.50 (hept, *J* = 6.9 Hz, 1H), 1.46 (d, *J* = 4.7 Hz, 3H), 1.45 (d, *J* = 4.7 Hz, 3H). ^13^C{^1^H} NMR (101 MHz, DMSO-*d*_6_): δ 153.7, 143.5, 135.7, 132.46, 134.44, 130.5, 129.7, 129.1, 128.9, 128.7, 125.5, 124.8, 119.6, 119.4, 109.9, 108.0, 43.9, 20.0, 19.7. HRMS (ESI) *m*/*z* calculated for C_20_H_17_N_2_O_4_S [M – H]^−^ 381.0904, found 381.0912.

#### Optical Resolution of (*R*_a_)-8-(3-Isopropyl-2-oxo-2,3-dihydro-1*H*-benzo[*d*]imidazol-1-yl)naphthalene-1-sulfonic Acid **6a**

Racemic sulfonic acid **6a** (2.0 g, 5.23 mmol) was placed into a 250 mL round-bottom flask. Water (40 mL) and (*S*)-(+)-1-cyclohexylethan-1-amine (0.67 mL, 5.23 mmol) were added slowly. After precipitation appeared, the mixture was heated at 70 °C. MeCN (15 × 2 mL) was added within 30 min. Then, the mixture was cooled at rt and after stirring for 2 h, a crystalline solid was collected by filtration. A diastereomeric salt of **6a** (0.96 g, 1.88 mmol) was suspended in MeOH (20 mL) and KOH (0.126 g, 2.26 mmol) was added. The mixture was stirred for 10 min and subsequently concentrated under vacuum. The residue was diluted with DCM (30 mL) and potassium salt of **6a** was extracted with water (30 mL). A water layer was washed again with DCM (30 mL), acidified with 35% aqueous HCl (3 mL), and extracted with EtOAc (4 × 40 mL). The combined organic layers were dried over MgSO_4_ and concentrated under vacuum to yield **(*****R***_**a**_**)-6a** (0.5 g, 25%, 99.9% ee) as a light pink solid, **[α]**_**D**_^**22**^ −159.83° (*c* 0.35, CHCl_3_/MeOH 1:1). The procedure was also used with (*R*)-(−)-1-cyclohexylethan-1-amine (1.33 g, 9.7 mmol) and ***rac*****-6a** (0.207 g, 0.54 mmol) to yield **(*****S***_**a**_**)-6b** (0.05 g, 25%, 99.9% ee), **[α]**_**D**_^**22**^ +166.66° (*c* 0.51, CHCl_3_/MeOH 1:1).

#### 8-(3-Cyclohexyl-2-oxo-2,3-dihydro-1*H*-benzo[*d*]imidazol-1-yl)naphthalene-1-sulfonic Acid **6b**

Diamine **4b** (1.98 g, 5.0 mmol) was dissolved in CHCl_3_ (80 mL). TEA (2.08 mL, 15.25 mmol) and triphosgene (1.49 g, 5.0 mmol) were subsequently added. The reaction was stirred at rt for 24 h, then washed with distilled water (3 × 50 mL), dried over MgSO_4_, and evaporated using RVO. To the residue, 20 mL of 10% HCl was added and the mixture was heated at 100 °C for 16 h. After cooling to rt, a precipitated solid was filtered off and purified by column chromatography (DCM/MeOH, 10:1). The product after purification was dissolved in methanolic HCl (120 g/L, 5 mL) and dried using a flow of nitrogen and vacuum to yield 1.2 g of **6b** as a white solid (57%). Melting point: 211–213 °C. ^1^H NMR (500 MHz, DMSO-*d*_6_): δ 8.23 (dd, *J* = 7.3, 1.3 Hz, 1H), 8.05 (dd, *J* = 8.2, 1.2 Hz, 1H), 8.04–8.01 (dd, *J* = 8.2, 1.2 Hz, 1H), 7.58 (t, *J* = 7.7 Hz, 1H), 7.51 (t, *J* = 7.7 Hz 1H), 7.37 (dd, *J* = 7.3, 1.2 Hz, 1H), 7.24 (d, *J* = 7.5 Hz, 1H), 6.93 (td, *J* = 7.8, 1.3 Hz, 1H), 6.87 (td, *J* = 7.6, 1.0 Hz, 1H), 6.75 (dd, *J* = 7.7, 1.0 Hz, 1H), 4.07 (tt, *J* = 12.3, 3.8 Hz, 1H), 2.15 (qd, *J* = 12.7, 3.6 Hz, 1H), 2.03 (qd, *J* = 12.5, 3.7 Hz, 1H), 1.90 (d, *J* = 13.2 Hz, 1H), 1.88–1.79 (m, 2H), 1.79–1.72 (m, 1H), 1.70–1.63 (m, 1H), 1.44–1.20 (m, 3H). ^13^C{^1^H} NMR (101 MHz, DMSO-*d*_6_): δ 153.7, 143.6, 135.7, 132.5, 132.4, 130.5, 129.7, 129.0, 128.9, 128.9, 125.4, 124.8, 119.5, 119.4, 109.9, 108.2, 52.0, 29.7, 29.2, 25.8, 25.1. HRMS (ESI) *m*/*z* calculated for C_23_H_21_N_2_O_4_S [M – H]^−^ 421.1217, found 421.1230.

#### Optical Resolution of (*S*_a_)-8-(3-Cyclohexyl-2-oxo-2,3-dihydro-1*H*-benzo[*d*]imidazol-1-yl)naphthalene-1-sulfonic Acid **6b**

Racemic sulfonic acid **6b** (1.25 g, 2.96 mmol) and, subsequently, (*R*)-(+)-1-(*p*-tolyl)ethan-1-amine (0.43 mL, 2.96 mmol) were added to water (12.5 mL). The mixture was heated at 70 °C. Then, *i*-PrOH (16 × 1.25 mL) was added during 30 min and the resulting mixture was stirred for 20 min at the same temperature. After that, the solution was cooled at rt and after stirring for 2 h, a diastereomeric salt of **6b** (0.57 g, 1.02 mmol) was collected by filtration. The salt was suspended in MeOH (15 mL). Then, KOH (0.069 g, 1.22 mmol) was added and the mixture was stirred for 10 min. The solvent was concentrated under vacuum and the residue was diluted with DCM (25 mL). A potassium salt of **6b** was extracted with water (25 mL). The water layer was washed again with DCM (25 mL), acidified with 35% aqueous HCl (2.5 mL), and extracted with EtOAc (4 × 35 mL). The combined organic layers were dried over MgSO_4_ and concentrated under vacuum to yield 0.386 g of **(*****S***_**a**_**)-6b** as a light pink solid (31%, 99.9% ee), **[α]**_**D**_^22^ = +153.35° (*c* 1.0, CHCl_3_/MeOH 1:1). The procedure was also used with (*S*)-(−)-1-(*p*-tolyl)ethan-1-amine (0.43 mL, 2.96 mmol) and ***rac*****-6b** (1.25 g, 2.96 mmol) to yield **(*****R***_**a**_**)-6b** (0.48 g, 38%, 99.9% ee), **[α]**_**D**_^22^ −148.43° (*c* 0.99, CHCl_3_/MeOH 1:1).

#### 8-(7-Chloro-3-isopropyl-2-oxo-2,3-dihydro-1*H*-benzo[*d*]imidazol-1-yl)naphthalene-1-sulfonic Acid **6c**

Diamine **4c** (1.88 g, 4.82 mmol) was dissolved in CHCl_3_ (80 mL). TEA (2.02 mL, 14.46 mmol) and triphosgene (1.43 g, 4.82 mmol) were subsequently added. The reaction was stirred at rt for 24 h. Then, the reaction was allowed to cool to rt and the mixture was diluted with 10% aqueous HCl (10 mL) and extracted with DCM (3 × 50 mL). The combined extracts were dried over MgSO_4_ and concentrated under vacuum. The residue was purified by column chromatography (DCM/MeOH, 10:1–5:1). The product after purification was dissolved in methanolic HCl (120 g/L, 5 mL) and dried using a flow of nitrogen and vacuum to yield 1.18 g of **6c** as a white solid (59%). Melting point: 180 °C. ^1^H NMR (400 MHz, CDCl_3_): δ 8.69 (dd, *J* = 7.7, 1.3 Hz, 1H), 8.29 (dd, *J* = 8.2, 1.0 Hz, 1H), 8.11 (dd, *J* = 8.2, 1.3 Hz, 1H), 7.76–7.70 (m, 1H), 7.67–7.61 (m, 2H), 7.15 (dd, *J* = 7.9, 1.1 Hz, 1H), 7.06 (t, *J* = 8.0 Hz, 1H), 6.98 (dd, *J* = 8.1, 1.1 Hz, 1H), 4.72 (hept, *J* = 7.0 Hz, 1H), 1.61 (d, *J* = 5.7 Hz, 3H), 1.59 (d, *J* = 5.7 Hz, 3H). ^13^C{^1^H} NMR (101 MHz, CDCl_3_): δ 154.9, 141.1, 137.9, 136.4, 134.3, 134.0, 131.5, 131.3, 131.0, 128.1, 127.6, 127.4, 124.6, 122.9, 122.4, 116.8, 107.7, 45.9, 20.2, 20.1. HRMS (ESI) *m*/*z* calculated for C_20_H_16_ClN_2_O_4_S [M – H]^−^ 415.0514, found 415.0524.

#### Optical Resolution of (*R*_a_)-8-(7-Chloro-3-isopropyl-2-oxo-2,3-dihydro-1*H*-benzo[*d*]imidazol-1-yl)naphthalene-1-sulfonic Acid **6c**

Racemic sulfonic acid **6c** (0.416 g, 1.00 mmol) was suspended in *i*-PrOH (4.2 mL). Then, (*R*)-(+)-1-(4-chlorophenyl)ethan-1-amine (0.140 mL, 1.00 mmol) was added followed by the addition of water (1.05 mL). The mixture was heated at 70 °C. *i*-PrOH (20 × 4.2 mL) was added portionwise during 30 min until all solid was dissolved. Then, the reaction mixture was pulled out from an oil bath to cool at rt. After the mixture was stirred for 20 h, a diastereomeric salt of **6c** (0.185 g, 0.32 mmol) was collected by filtration. The solid was suspended in MeOH (5 mL), and KOH (0.022 g, 0.38 mmol) was subsequently added. The resulting mixture was stirred for 10 min and then concentrated under vacuum. The residue was diluted with DCM (10 mL), and potassium salt of **6c** was extracted with water (15 mL). The water layer was washed again with DCM (10 mL), acidified with 35% HCl (1.5 mL), and extracted with EtOAc (4 × 20 mL). The combined organic layers were dried over MgSO_4_ and concentrated under vacuum to yield 0.098 g (23%, 99.9% ee) of **(*****R***_**a**_**)-6c** as a light pink solid, **[α]**_**D**_^**22**^ +100.87° (*c* = 0.3, CHCl_3_/MeOH 1:1). The procedure was also used with (*S*)-(−)-1-(4-chlorophenyl)ethan-1-amine (0.140 mL, 1.00 mmol) and ***rac*****-6c** (0.416 g, 1.00 mmol) to yield **(*****S***_**a**_**)-6c** (0.12 g, 28%, 99.9% ee), **[α]**_**D**_^**22**^ −103.62° (*c* 0.30, CHCl_3_/MeOH 1:1).

#### 8-(7-Chloro-3-cyclohexyl-2-oxo-2,3-dihydro-1*H*-benzo[*d*]imidazol-1-yl)naphthalene-1-sulfonic Acid **6d**

Diamine **4d** (1.56 g, 3.62 mmol) was dissolved in CHCl_3_ (58 mL). TEA (1.51 mL, 10.86 mmol) and triphosgene (1.07 g, 3.62 mmol) were subsequently added. The reaction mixture was stirred at rt for 24 h. The mixture was washed with water (3 × 50 mL), dried over MgSO_4_, and concentrated under vacuum. To the residue, 10% HCl (25 mL) was added, and the mixture was heated at 100 °C for 16 h. Then, the reaction was allowed to cool to rt and the mixture was diluted with 10% aqueous HCl (10 mL) and extracted with DCM (3 × 50 mL). The residue was purified by column chromatography (DCM/MeOH, 10:1–5:1). The product after purification was dissolved in methanolic HCl (120 g/L, 4 mL) and dried using a flow of nitrogen and vacuum to yield 1.22 g of **6d** as a white solid (74%). Melting point: 223–225 °C. ^1^H NMR (400 MHz, DMSO-*d*_6_): δ 8.31 (dd, *J* = 7.3, 1.4 Hz, 1H), 8.04 (dd, *J* = 8.2, 1.4 Hz, 1H), 8.00 (dd, *J* = 8.3, 1.4 Hz, 1H), 7.56–7.51 (m, 1H), 7.51–7.43 (m, 1H), 7.32 (dd, *J* = 7.3, 1.4 Hz, 1H), 7.21 (dd, *J* = 7.9, 0.9 Hz, 1H), 6.89 (t, *J* = 8.0 Hz, 1H), 6.79 (dd, *J* = 8.1, 0.8 Hz, 1H), 4.10 (tt, *J* = 13.0, 4.2 Hz, 1H), 2.16 (qd, *J* = 12.4, 3.4 Hz, 1H), 2.02 (qd, *J* = 12.7, 3.0 Hz, 1H), 1.96 (m, 1H), 1.91 (d, *J* = 13.1 Hz, 1H), 1.80 (ddd, *J* = 25.4, 12.6, 2.2 Hz, 3H), 1.71–1.59 (m, 1H), 1.42–1.24 (m, 3H). ^13^C{^1^H} NMR (101 MHz, DMSO-*d*_6_): δ 154.0, 143.9, 135.5, 132.2, 131.1, 130.8, 130.6, 130.2, 129.3, 128.9, 128.3, 124.7, 124.5, 121.1, 120.0, 115.1, 107.0, 52.4, 29.5, 29.1, 25.7, 25.0. HRMS (ESI) *m*/*z* calculated for C_23_H_20_ClN_2_O_4_S [M – H]^−^ 455.0827, found 455.0838.

#### 8–2-[(Dimethylamino)-3-isopropyl-*1H*-benzo[*d*]imidazol-3-ium-1-yl]naphthalene-1-sulfonate **7a**

Phosgene dimethyliminium chloride (0.136 g, 0.84 mmol) was added in one portion to a solution of diamine **4a** (0.20 g, 0.56 mmol) and TEA (0.47 mL, 3.36 mmol) in CHCl_3_ (4 mL). The reaction mixture was allowed to stir at rt for 2 h. After the starting material disappeared, the reaction mixture was quenched with a few drops of MeOH and concentrated under vacuum. The residue was dissolved in DCM (2 mL). Then, the addition of *n*-hexane (5 mL) and ultrasonic irradiation for 30 s gave a precipitate, which was collected by filtration to yield 0.16 g (67%) of **7a** as a yellow solid. Melting point: 315 °C (decomposition). ^1^H NMR (500 MHz, CDCl_3_): δ 8.81 (dd, *J* =7.1, 0.7 Hz, 1H), 8.15 (dd, *J* = 8.2, 1.3 Hz, 1H), 7.99 (dd, *J* = 8.2, 1.4 Hz, 1H), 7.63–7.51 (m, 2H), 7.56 (d, *J* = 8.2 Hz, 1H), 7.42 (d, *J* = 7.7, 1.5 Hz, 1H), 7.31 (td, *J* = 7.9, 1.3 Hz, 1H), 7.24 (t, *J* = 8.9 Hz, 1H), 6.99–6.56 (m, 1H), 4.71 (hept, *J* = 6.9 Hz, 1H), 2.82 (s, 6H), 1.84 (d, *J* = 7.0 Hz, 3H), 1.82 (d, *J* = 6.8 Hz, 3H). ^13^C{^1^H} NMR (126 MHz, CDCl_3_): δ 154.1, 142.4, 136.5, 136.1, 133.4, 132.6, 131.4, 129.8, 129.2, 128.0, 127.4, 127.1, 124.9, 124.7, 124.2, 113.9, 113.0, 52.1, 42.5, 21.3, 20.9. HRMS (ESI) *m*/*z* calculated for C_22_H_24_N_3_O_3_S [M + H]^+^ 410.1533, found 410.1531.

#### 8-[3-Cyclohexyl-2-(dimethylamino)-*1H*-benzo[*d*]imidazol-3-ium-1-yl]naphthalene-1-sulfonate **7b**

Phosgene dimethyliminium chloride (0.043 g, 0.266 mmol) was added in one portion to a solution of diamine **4b** (0.070 g, 0.177 mmol) and triethylamine (0.15 mL, 1.06 mmol) in CHCl_3_ (2 mL). The reaction mixture was allowed to stir at rt for 2 h. After the starting material disappeared, the reaction mixture was quenched with a few drops of MeOH and concentrated under vacuum. The residue was dissolved in DCM (1 mL). Then, the addition of *n*-hexane (3 mL) and ultrasonic irradiation for 30 s gave a precipitate, which was collected by filtration to yield 0.060 g (75%) of **7b** as a yellow solid. Meting point: 299 °C (decomposition). ^1^H NMR (500 MHz, CDCl_3_): δ 8.81 (dd, *J* = 7.4, 1.4 Hz, 1H), 8.14 (dd, *J* = 8.2, 1.3 Hz, 1H), 7.98 (dd, *J* = 8.2, 1.4 Hz, 1H), 7.68–7.53 (m, 3H), 7.41 (dd, *J* = 7.2, 1.4 Hz, 1H), 7.30 (td, *J* = 8.4, 7.9, 1.1 Hz, 1H), 7.22 (td, *J* = 7.9, 0.9 Hz, 1H), 6.99–6.89 (m, 1H), 4.23 (tt, *J* = 12.4, 3.8 Hz, 1H), 2.82 (s, 6H), 2.45–2.52 (m, 1H), 2.40 (qd, *J* = 12.7, 3.8 Hz, 1H), 2.21 (qd, *J* = 12.8, 3.8 Hz, 2H), 2.12–2.04 (m, 1H), 2.03–1.90 (m, 1H), 1.86–1.80 (m, 1H), 1.53–1.41 (m, 2H), 1.36 (tt, *J* = 13.0, 3.6 Hz, 1H). ^13^C{^1^H} NMR (126 MHz, CDCl_3_): δ 154.3, 142.5, 136.5, 136.1, 133.3, 132.5, 131.4, 129.8, 129.3, 128.0, 127.8, 127.0, 124.9, 124.6, 124.0, 113.8, 113.5, 60.2, 42.5, 31.5, 30.4, 26.6, 26.1, 25.4. HRMS (ESI) *m*/*z* calculated for C_25_H_28_N_3_O_3_S [M + H]^+^ 450.1846, found 450.1843.

#### 8-[7-Chloro-2-(dimethylamino)-3-isopropyl-1*H*-benzo[*d*]imidazol-3-ium-1-yl]naphthalene-1-sulfonate **7c**

Phosgene dimethyliminium chloride (0.022 g, 0.13 mmol) was added in one portion to a solution of diamine **4c** (0.035 mg, 0.09 mmol) and TEA (0.07 ml, 0.53 mmol) in CHCl_3_ (1.4 mL). The reaction mixture was allowed to stir at rt for 2 h. After the starting material disappeared, the reaction mixture was quenched with a few drops of MeOH and concentrated under vacuum. The residue was precipitated by addition of MeCN (5 mL) and collected by filtration as a white solid to yield 0.021 g (53%) of **7c**. Melting point: 307 °C (decomposition). ^1^H NMR (500 MHz, DMSO-*d*_6_): δ 8.49 (dd, *J* = 7.4, 1.3 Hz, 1H), 8.30 (dd, *J* = 8.2, 1.2 Hz, 1H), 8.15 (dd, *J* = 8.2, 1.2 Hz, 1H), 7.88 (dd, *J* = 7.3, 1.3 Hz, 1H), 7.82 (d, *J* = 8.2 Hz, 1H), 7.72–7.65 (m, 1H), 7.61 (t, *J* = 7.8 Hz, 1H), 7.26 (t, *J* = 8.1 Hz, 1H), 7.18–7.14 (m, 1H), 4.72 (hept, *J* = 6.7 Hz, 1H), 2.75 (s, 6H), 1.82 (d, *J* = 7.0 Hz, 3H), 1.60 (d, *J* = 6.8 Hz, 3H). ^13^C{^1^H} NMR (126 MHz, DMSO-*d*_6_): δ 155.3, 142.5, 135.4, 133.1, 131.5, 131.4, 131.3, 130.4, 129.5, 129.4, 128.8, 125.7, 125.1, 124.9, 123.6, 118.3, 112.2, 51.6, 41.8, 20.1, 19.8. HRMS (ESI) *m*/*z* calculated for C_22_H_23_ClN_3_O_3_S [M + H]^+^ 444.1143, found 444.1146.

#### 8-[7-Chloro-3-cyclohexyl-2-(dimethylamino)-1*H*-benzo[*d*]imidazol-3-ium-1-yl]naphthalene-1-sulfonate **7d**

Phosgene dimethyliminium chloride (0.029 g, 0.18 mmol) was added to a solution of diamine **4d** (0.050 g, 0.12 mmol) and TEA (0.01 mL, 0.7 mmol) in CHCl_3_ (1.5 mL). The reaction mixture was allowed to stir at rt for 2 h. After the starting material disappeared, the reaction mixture was quenched with a few drops of MeOH and concentrated under vacuum. Compound **7d** was isolated as a white solid after purification by semipreparative HPLC to yield 0.018 g (31%). Melting point: 308 °C (decomposition). ^1^H NMR (500 MHz, DMSO-*d*_*6*_): δ 8.47 (dd, *J =* 7.4, 1.4 Hz, 1H), 8.26 (dd, *J =* 8.3, 1.3 Hz, 1H), 8.10 (dd, *J =* 8.2, 1.2 Hz, 1H), 7.81 (dd, *J =* 7.5, 1.6 Hz, 2H), 7.68–7.60 (m, 1H), 7.60–7.48 (m, 1H), 7.23 (t, *J =* 8.1 Hz, 1H), 7.12 (dd, *J =* 8.0, 0.7 Hz, 1H), 4.22 (tt, *J =* 12.2, 4.3 Hz, 1H), 2.74 (s, 6H), 2.38 (qd, *J =* 12.3, 3.6 Hz, 2H), 2.29–2.21 (m, 1H), 2.12–2.05 (m, 2H), 1.99–1.91 (m, 1H), 1.91–1.81 (m, 1H), 1.74–1.65 (m, 1H), 1.57–1.35 (m, 3H). ^13^C{^1^H} NMR (126 MHz, DMSO-*d*_*6*_): δ 155.2, 142.7, 135.2, 132.8, 131.2, 131.0, 131.0, 130.1, 129.5, 129.3, 128.6, 125.4, 125.0, 124.5, 123.3, 118.3, 112.4, 59.4, 41.6, 29.6, 29.3, 25.5, 25.1, 24.3. HRMS (ESI) *m*/*z* calculated for C_25_H_27_ClN_3_O_3_S [M + H]^+^ 484.1456, found 484.1457.

#### 8-(2-Ethyl-1*H*-benzo[*d*]imidazol-1-yl)-*N*-((trifluoromethyl)sulfonyl)naphthalene-1-sulfonamide **9b**

POCl_3_ (1.2 mL) was added to sulfonic acid **5b** (0.353 g, 1.0 mmol). The resulting mixture was stirred at 100 °C for 3 h. After the reaction was completed, the mixture was cooled to rt and ice-cold 10% aqueous solution of K_2_CO_3_ (20 mL) was added carefully. The product was extracted with DCM (3 × 20 mL). The combined extracts were dried over MgSO_4_ and concentrated under vacuum to yield crude sulfonyl chloride, which was redissolved in MeCN (4 mL). K_2_CO_3_ (0.28 g, 2 mmol) and CF_3_SO_2_NH_2_ (0.15 g, 1 mmol) were subsequently added and the resulting mixture was stirred at rt for another 48 h. The reaction mixture was neutralized to pH 1 with 35% aqueous HCl and concentrated under vacuum. **9b** was purified using column chromatography (DCM/MeOH, 100:1–20:1). Subsequent crystallization from a mixture of MeCN /water (1:5, 60 mL) gave a white solid (0.314 g, 65%). Melting point: 272–274 °C. ^1^H NMR (400 MHz, DMSO-*d*_*6*_): δ 14.79 (br. s., 1H), 8.49–8.45 (m, 2H), 8.41 (dd, *J* = 8.4, 1.2 Hz, 1H), 7.90–7.85 (m, 2H), 7.83–7.77 (m, 2H), 7.55–7.50 (m, 1H), 7.44–7.39 (m, 1H), 7.13 (dt, *J* = 8.3, 0.8 Hz, 1H), 3.04–2.91 (m, 1H), 2.63–2.52 (m, 1H), 1.20 (t, *J* = 7.5 Hz, 3H). ^13^C{^1^H} NMR (101 MHz, DMSO-*d*_*6*_): δ 157.8, 139.4, 135.9, 135.8, 134.1, 133.4, 131.9, 131.6, 129.8, 127.4, 126.7, 125.8, 125.4, 125.2, 125.0, 119.7 (q, *J* = 324.6 Hz), 113.9, 113.4, 20.7, 9.7. ^19^F NMR (376 MHz, DMSO-*d*_*6*_): δ −76.87. HRMS (ESI) *m*/*z* calculated for C_20_H_15_F_3_N_3_O_4_S_2_ [M – H]^−^ 482.0451, found 482.0463. The procedure was also used with **(*****S***_**a**_**)-5b** (0.209 g, 0.59 mmol) to yield **(*****S***_**a**_**)-9b** (0.252 g, 87%, 99.9% ee), **[α]**_**D**_^**22**^ −17.13° (*c* 0.96, CHCl_3_/MeOH 1:1).

#### 8-(7-Chloro-2-ethyl-1*H*-benzo[*d*]imidazol-1-yl)-*N*-((trifluoromethyl)sulfonyl)naphthalene-1-sulfonamide **9e**

POCl_3_ (1.2 mL) was added to sulfonic acid **5e** (0.353 g, 1.0 mmol). The resulting mixture was stirred at 100 °C for 3 h. After the reaction was completed, the mixture was cooled to rt and ice-cold 10% aqueous solution of K_2_CO_3_ (20 mL) was added carefully. The product was extracted with DCM (3 × 20 mL). The combined extracts were dried over MgSO_4_ and concentrated under vacuum to yield crude sulfonyl chloride, which was redissolved in MeCN (4 mL). K_2_CO_3_ (0.414 g, 3 mmol) and CF_3_SO_2_NH_2_ (0.3 g, 2 mmol) were subsequently added and the resulting mixture was stirred at rt for another 48 h. The reaction mixture was concentrated under vacuum and the residue was purified by column chromatography (DCM/MeOH, 100:1–10:1). The purified product was dissolved in a mixture of MeOH/H_2_O (1:1, 10 mL), acidified with 48% aqueous solution of HBr to pH = 1, and stirred at rt for 48 h. The solid was collected by filtration and recrystallized from the mixture of MeCN/H_2_O (1:5, 60 mL). **9e** was isolated as a white solid (0.256 g, 49%). Melting point: 281–286 °C. ^1^H NMR (400 MHz, DMSO-*d*_*6*_): δ 8.52 (dd, *J* = 7.5, 1.3 Hz, 1H), 8.46 (dd, *J* = 8.3, 1.3 Hz, 1H), 8.41 (dd, *J* = 8.3, 1.2 Hz, 1H), 7.97 (dd, *J* = 7.4, 1.4 Hz, 1H), 7.86–7.76 (m, 3H), 7.50 (t, *J* = 8.0 Hz, 1H), 7.43 (dd, *J* = 7.9, 1.0 Hz, 1H), 3.04–2.90 (m, 1H), 2.61–2.52 (m, 1H), 1.26 (t, *J* = 7.5 Hz, 3H). ^13^C{^1^H} NMR (101 MHz, DMSO-*d*_*6*_): δ 159.5, 139.3, 135.8, 134.3, 133.8, 132.9, 131.9, 131.7, 131.4, 127.8, 126.7, 126.1, 126.0, 125.7, 125.6, 119.9 (q, *J* = 324.8 Hz), 118.9, 112.9, 21.1, 9.8. ^19^F NMR (376 MHz, DMSO-*d*_*6*_): δ −76.68. HRMS (ESI) *m*/*z* calculated for C_20_H_14_ClF_3_N_3_O_4_S_2_ [M – H]^−^ 516.0061, found 516.0070.

#### 8-(3-Isopropyl-2-oxo-2,3-dihydro-1*H*-benzo[*d*]imidazol-1-yl)naphthalene-1-sulfonyl Chloride **10a**

Sulfonic acid **6a** (0.382 g, 1.0 mmol) was suspended in CHCl_3_ (17 mL). TEA (0.426 mL, 3.0 mmol) and triphosgene (0.297 g, 1.0 mmol) were subsequently added and the resulting mixture was allowed to stir at rt for 16 h. After the reaction was completed, the mixture was diluted with DCM (15 mL) and washed with 10% aqueous solution of HCl (30 mL), 10% aqueous solution of K_2_CO_3_ (30 mL), water (30 mL), and brine (30 mL). The organic layer was dried over MgSO_4_ and concentrated under vacuum to yield 0.34 g (84%) of **10a** as a white amorphous solid. Melting point: 140–144 °C. ^1^H NMR (500 MHz, CDCl_3_): δ 8.62 (dd, *J* = 7.6, 1.2 Hz, 1H), 8.27 (dd, *J* = 8.3, 1.0 Hz, 1H), 8.08 (dd, *J* = 8.0, 1.4 Hz, 1H), 7.77–7.72 (m, 1H), 7.70 (dd, *J* = 7.4, 1.5 Hz, 1H), 7.65 (t, *J* = 8 Hz, 1H), 7.24 (ddd, *J* = 7.9, 1.0, 0.5 Hz, 1H), 7.15 (td, *J* = 7.8, 1.2 Hz, 1H), 7.05 (td, *J* = 7.7, 1.1 Hz, 1H), 6.96–6.91 (m, 1H), 4.72 (hept, *J* = 7.0 Hz, 1H), 1.59 (dd, *J* = 7.0, 4.5 Hz, 6H). ^13^C{^1^H} NMR (126 MHz, CDCl_3_): δ 154.6, 141.1, 137.5, 136.5, 133.8, 132.4, 131.8, 131.5, 131.0, 129.4, 128.0, 127.6, 124.8, 120.0, 121.0, 110.4, 109.2, 45.5, 20.3, 20.3. HRMS (ESI) *m*/*z* calculated for C_20_H_18_ClN_2_O_3_S [M + H]^+^ 401.0721, found 401.0724.

#### 8-(3-Cyclohexyl-2-oxo-2,3-dihydro-1*H*-benzo[*d*]imidazol-1-yl)naphthalene-1-sulfonyl Chloride **10b**

Sulfonic acid **6b** (0.211 mg, 0.5 mmol) was suspended in CHCl_3_ (9 mL). TEA (0.214 mL, 1.51 mmol) and triphosgene (0.148 g, 0.5 mmol) were subsequently added and the resulting mixture was allowed to stir at rt for 16 h. After the reaction was completed, the mixture was diluted with DCM (15 mL) and washed with 10% aqueous solution of HCl (30 mL), 10% aqueous solution of K_2_CO_3_ (30 mL), water (30 mL), and brine (30 mL). The organic layer was dried over MgSO_4_ and concentrated under vacuum to yield 0.214 g (97%) of **10b** as a yellow amorphous solid. Melting point: 162–166 °C. ^1^H NMR (500 MHz, CDCl_3_): δ 8.61 (dd, *J* = 7.6, 1.2 Hz, 1H), 8.27 (dd, *J* = 8.2, 0.9 Hz, 1H), 8.08 (dd, *J* = 8.0, 1.4 Hz, 1H), 7.76–7.71 (m, 1H), 7.69 (dd, *J* = 7.4, 1.5 Hz, 1H), 7.65 (t, *J* = 7.9 Hz, 1H), 7.28–7.26 (m, 1H), 7.14 (td, *J* = 7.8, 1.2 Hz, 1H), 7.04 (td, *J* = 7.7, 1.0 Hz, 1H), 6.95–6.92 (m, 1H), 4.27 (tt, *J* = 12.4, 3.9 Hz, 1H), 2.27–2.14 (m, 2H), 2.03–1.84 (m, 4H), 1.78–1.71 (m, 1H), 1.51–1.38 (m, 2H), 1.35–1.23 (m, 1H). ^13^C{^1^H} NMR (126 MHz, CDCl_3_): δ 154.7, 141.0, 137.4, 136.5, 133.8, 132.3, 131.7, 131.6, 130.9, 129.8, 127.9, 127.6, 124.7, 121.9, 120.9, 110.4, 109.4, 53.5, 30.2, 26.2, 25.6. HRMS (ESI) *m*/*z* calculated for C_23_H_22_ClN_2_O_3_S [M + H]^+^ 441.1034, found 441.1037.

#### 8-(7-Chloro-3-isopropyl-2-oxo-2,3-dihydro-1*H*-benzo[*d*]imidazol-1-yl)naphthalene-1-sulfonyl Chloride **10c**

Sulfonic acid **6c** (0.16 g, 0.385 mmol) was suspended in CHCl_3_ (6.4 mL). TEA (0.16 mL, 1.16 mmol) and triphosgene (0.114 g, 0.385 mmol) were subsequently added and the resulting mixture was allowed to stir at rt for 16 h. After the reaction was completed, the mixture was diluted with DCM (5 mL) and washed with 10% aqueous solution of HCl (10 mL), 10% aqueous solution of K_2_CO_3_ (10 mL), water (10 mL), and brine (10 mL). The organic layer was dried over MgSO_4_ and concentrated under vacuum to yield 0.16 g (94%) of **10c** as a light yellow amorphous solid. Melting point: 108–112 °C. ^1^H NMR (500 MHz, CDCl_3_): δ 8.69 (dd, *J* = 7.7, 1.3 Hz, 1H), 8.29 (dd, *J* = 8.2, 1.2 Hz, 1H), 8.11 (dd, *J* = 8.1, 1.3 Hz, 1H), 7.76–7.69 (m, 1H), 7.68–7.60 (m, 2H), 7.15 (dd, *J* = 8.0, 1.0 Hz, 1H), 7.06 (t, *J* = 8.1 Hz, 1H), 6.98 (dd, *J* = 8.2, 1.0 Hz, 1H), 4.72 (hept, *J* = 7.0 Hz, 1H), 1.59 (t, *J* = 7.0 Hz, 6H). ^13^C{^1^H} NMR (126 MHz, CDCl_3_): δ 154.9, 141.1, 137.9, 136.4, 134.3, 134.0, 131.5, 131.3, 131.1, 128.1, 127.6, 127.4, 124.5, 122.9, 122.4, 116.8, 107.7, 45.9, 20.2, 20.1. HRMS (ESI) *m*/*z* calculated for C_20_H_17_Cl_2_N_2_O_3_S [M + H]^+^ 435.0331, found 435.0337. The procedure was also used with **(*****R***_**a**_**)-6c** (0.08 g, 0.19 mmol) to yield **(*****R***_**a**_**)-10c** (0.071 g, 85%), **[α]**_**D**_^**22**^ +101.34° (*c* 0.27, CHCl_3_).

#### 8-(7-Chloro-3-cyclohexyl-2-oxo-2,3-dihydro-1*H*-benzo[*d*]imidazol-1-yl)naphthalene-1-sulfonyl Chloride **10d**

Sulfonic acid **6d** (0.098 mg, 0.215 mmol) was suspended in CHCl_3_ (3.6 mL). TEA (0.09 mL, 0.645 mmol) and triphosgene (0.063 g, 0.215 mmol) were subsequently added and the resulting mixture was allowed to stir at rt for 16 h. After the reaction was completed, the mixture was diluted with DCM (5 mL) and washed with 10% aqueous solution of HCl (10 mL), 10% aqueous solution of K_2_CO_3_ (10 mL), water (10 mL), and brine (10 mL). The organic layer was dried over MgSO_4_ and concentrated under vacuum to yield 0.09 g (87%) of **10d** as a light yellow solid. Melting point: 184–190 °C. ^1^H NMR (500 MHz, CDCl_3_): δ 8.69 (dd, *J* = 7.7, 1.3 Hz, 1H), 8.28 (dd, *J* = 8.2, 1.2 Hz, 1H), 8.10 (dd, *J* = 8.1, 1.3 Hz, 1H), 7.77–7.67 (m, 1H), 7.67–7.57 (m, 2H), 7.18 (dd, *J* = 8.0, 0.9 Hz, 1H), 7.06 (t, *J* = 8.1 Hz, 1H), 6.98 (dd, *J* = 8.2, 0.9 Hz, 1H), 4.26 (tt, *J* = 12.5, 3.8 Hz, 1H), 2.29–2.13 (m, 2H), 2.04–1.86 (m, 4H), 1.79–1.71 (m, 1H), 1.50–1.37 (m, 2H), 1.35–1.20 (m, 2H). ^13^C{^1^H} NMR (126 MHz, CDCl_3_): δ 155.0, 141.1, 137.9, 136.4, 134.2, 134.0, 131.6, 131.5, 131.1, 128.1, 127.6, 127.3, 124.5, 122.8, 122.3, 116.7, 107.9, 54.0, 30.0, 26.2, 25.5. HRMS (ESI) *m*/*z* calculated for C_23_H_21_Cl_2_N_2_O_3_S [M + H]^+^ 475.0644, found 475.0648.

#### 8-(3-Isopropyl-2-oxo-2,3-dihydro-1*H*-benzo[*d*]imidazol-1-yl)-*N*-((trifluoromethyl)sulfonyl)naphthalene-1-sulfonamide **11a**

Sulfonyl chloride **10a** (0.098 g, 0.244 mmol) was dissolved in MeCN (1.4 mL). K_2_CO_3_ (0.10 g, 0.73 mmol) and CF_3_SO_2_NH_2_ (0.073 g, 0.49 mmol) were subsequently added and the resulting mixture was stirred at rt overnight. After the reaction was completed, the reaction mixture was concentrated under vacuum and the residue was purified by column chromatography (DCM/MeOH, 50:1–10:1). The purified product was dissolved in a methanolic solution of HCl (120 g/L, 0.5 mL) and then dried using a flow of nitrogen and vacuum to yield 0.1 g (81%) of **11a** as a white solid. Melting point: 218–220 °C. ^1^H NMR (500 MHz, DMSO-*d*_6_): δ 8.36 (dd, *J* = 7.4, 1.2 Hz, 1H), 8.18 (dd, *J* = 8.1, 0.9 Hz, 1H), 8.13 (dd, *J* = 8.1, 1.2 Hz, 1H), 7.70–7.64 (m, 1H), 7.64–7.59 (m, 1H), 7.47 (dd, *J* = 7.3, 1.3 Hz, 1H), 7.26–7. 24 (m, 1H), 7.02–6.98 (m, 1H), 6.95–6.92 (m, 2H), 4.53 (hept, *J* = 6.9 Hz, 1H), 1.46 (d, *J* = 7.0 Hz, 6H). ^13^C{^1^H} NMR (126 MHz, DMSO-*d*_6_): δ 153.6, 141.5, 135.8, 132.6, 132.2, 131.8, 129.9, 129.4, 128.9, 128.2, 126.3, 124.7, 120.4, 120.1 (q, *J* = 324.5 Hz), 119.9, 110.5, 108.4, 44.2, 20.1, 19.8. ^19^F NMR (376 MHz, DMSO-*d*_*6*_): δ −77.12. HRMS (ESI) *m*/*z* calculated for C_21_H_17_F_3_N_3_O_5_S_2_ [M – H]^−^ 512.0556, found 512.0566.

#### 8-(3-Cyclohexyl-2-oxo-2,3-dihydro-1*H*-benzo[*d*]imidazol-1-yl)-*N*-((trifluoromethyl)sulfonyl)naphthalene-1-sulfonamide **11b**

Sulfonyl chloride **10b** (0.060 g, 0.135 mmol) was dissolved in MeCN (0.6 mL). K_2_CO_3_ (0.056 g, 0.405 mmol) and CF_3_SO_2_NH_2_ (0.040 g, 0.27 mmol) were subsequently added and the resulting mixture was stirred at rt overnight. After the reaction was completed, the reaction mixture was concentrated under vacuum and the residue was purified by column chromatography (DCM/MeOH, 50:1–10:1). The purified product was dissolved in a methanolic solution of HCl (120 g/L, 0.5 mL) and then dried using a flow of nitrogen and vacuum to yield 0.067 g (91%) of **11b** as a white solid. Melting point: 161–163 °C. ^1^H NMR (400 MHz, DMSO-*d*_6_): δ 8.35 (dd, *J* = 7.4, 1.2 Hz, 1H), 8.17 (dd, *J* = 8.3, 1.0 Hz, 1H), 8.12 (dd, *J* = 8.2, 1.1 Hz, 1H), 7.66 (t, *J* = 7.9 Hz, 1H), 7.61 (t, *J* = 8.2 Hz, 1H), 7.47 (dd, *J* = 7.4, 1.4 Hz, 1H), 7.28 (d, *J* = 7.7 Hz, 1H), 7.03–6.95 (m, 1H), 6.94–6.91 (m, 2H), 4.10 (tt, *J* = 12.6, 3.7 Hz, 1H), 2.14 (qd, *J* = 12.7, 3.9 Hz, 1H), 2.03 (qd, *J* = 11.9, 2.8 Hz, 1H), 1.94–1.78 (m, 3H), 1.78–1.58 (m, 2H), 1.47–1.21 (m, 3H). ^13^C{^1^H} NMR (126 MHz, DMSO-*d*_6_): δ 153.5, 141.3, 135.6, 132.5, 132.0, 131.6, 129.7, 129.7, 129.2, 129.0, 128.0, 126.2, 124.5, 120.2, 119.9 (q, *J* = 324.3 Hz), 119.7, 110.3, 108.5, 52.1, 29.6, 29.2, 25.7, 25.0. ^19^F NMR (376 MHz, DMSO-*d*_*6*_): δ −77.18. HRMS (ESI) *m*/*z* calculated for C_24_H_23_F_3_N_3_O_5_S_2_ [M + H]^+^ 554.1026, found 554.1024.

#### 8-(7-Chloro-3-isopropyl-2-oxo-2,3-dihydro-1*H*-benzo[*d*]imidazol-1-yl)-*N*-((trifluoromethyl)sulfonyl)naphthalene-1-sulfonamide **11c**

Sulfonyl chloride **10c** (0.157 g, 0.36 mmol) was dissolved in MeCN (1.6 mL). K_2_CO_3_ (0.145 g, 1.08 mmol) and CF_3_SO_2_NH_2_ (0.107 g, 0.72 mmol) were subsequently added and the resulting mixture was stirred at rt overnight. After the reaction was completed, the reaction mixture was concentrated under vacuum and the residue was purified by column chromatography (DCM/MeOH, 50:1–10:1). The purified product was dissolved in a methanolic solution of HCl (120 g/L, 0.5 mL) and then dried using a flow of nitrogen and vacuum to yield 0.145 g (74%) of **11c** as a white solid. Melting point: 184–186 °C. ^1^H NMR (400 MHz, DMSO-*d*_6_): δ 8.47 (dd, *J* = 7.5, 1.3 Hz, 1H), 8.21 (dd, *J* = 8.4, 1.2 Hz, 1H), 8.16 (dd, *J* = 8.3, 1.3 Hz, 1H), 7.70–7.55 (m, 2H), 7.44 (dd, *J* = 7.3, 1.4 Hz, 1H), 7.25 (dd, *J* = 7.9, 1.0 Hz, 1H), 6.98 (t, *J* = 8.0 Hz, 1H), 6.89 (dd, *J* = 8.2, 1.0 Hz, 1H), 4.58 (hept, *J* = 7.0 Hz, 1H), 1.46 (d, *J* = 7.0 Hz, 6H). ^13^C{^1^H} NMR (126 MHz, DMSO-*d*_6_): δ 153.7, 141.0, 135.5, 132.9, 132.00, 131.4, 130.7, 130.4, 129.8, 128.4, 127.6, 125.6, 124.4, 121.48, 120.85, 120.0 (q, *J =* 324.2 Hz), 115.26, 107.16, 44.49, 19.71, 19.46. ^19^F NMR (376 MHz, DMSO-*d*_*6*_): δ −76.92. HRMS (ESI) *m*/*z* calculated for C_21_H_16_ClF_3_N_3_O_5_S_2_ [M – H]^−^ 546.0167, found 546.0179. The procedure was also used with **(*****R***_**a**_**)-10c** (0.065 g, 0.15 mmol) to yield **(*****R***_**a**_**)-11c** (0.056 g, 68%, 99.9% ee), **[α]**_**D**_^**22**^ +10.61 (*c* 0.38, CHCl_3_).

#### 8-(7-Chloro-3-cyclohexyl-2-oxo-2,3-dihydro-1*H*-benzo[*d*]imidazol-1-yl)-*N*-((trifluoromethyl)sulfonyl)naphthalene-1-sulfonamide **11d**

Sulfonyl chloride **10d** (0.073 g, 0.168 mmol) was dissolved in MeCN (0.8 mL). K_2_CO_3_ (0.07 g, 1.504 mmol) and CF_3_SO_2_NH_2_ (0.050 mg, 0.336 mmol) were subsequently added and the resulting mixture was stirred at rt overnight. After the reaction was completed, the reaction mixture was concentrated under vacuum and the residue was purified by column chromatography (DCM/MeOH, 50:1–10:1). The purified product was dissolved in a methanolic solution of HCl (120 g/L, 0.5 mL) and then dried using a flow of nitrogen and vacuum to yield 0.068 g (69%) of **11d** as a white solid. Melting point: 209–211 °C. ^1^H NMR (500 MHz, DMSO-*d*_6_): δ 8.48 (dd, *J* = 7.5, 1.3 Hz, 1H), 8.21 (dd, *J* = 8.4, 1.2 Hz, 1H), 8.16 (dd, *J* = 8.3, 1.3 Hz, 1H), 7.67–7.63 (m, 1H), 7.63–7.58 (m, 1H), 7.43 (dd, *J* = 7.2, 1.3 Hz, 1H), 7.29 (dd, *J* = 8.0, 0.9 Hz, 1H), 6.98 (t, *J* = 8.1 Hz, 1H), 6.88 (dd, *J* = 8.2, 0.9 Hz, 1H), 4.15 (tt, *J* = 12.4, 3.8 Hz, 1H), 2.15 (qd, *J* = 13.4, 13.0, 3.8 Hz, 1H), 2.05 (qd, *J* = 12.5, 3.7 Hz, 1H), 1.95–1.72 (m, 4H), 1.70–1.61 (m, 1H), 1.37 (qd, *J* = 12.9, 3.0 Hz, 2H), 1.28 (tt, *J* = 12.1, 3.2 Hz, 1H). ^13^C{^1^H} NMR (126 MHz, DMSO-*d*_6_): δ 153.8, 141.0, 135.5, 132.9, 131.9, 131.4, 131.0, 130.4, 129.8, 128.4, 127.6, 125.6, 124.4, 121.5, 120.8, 120.0, 115.2, 107.4, 52.5, 29.4, 29.0, 25.6, 25.6, 24.9. ^19^F NMR (376 MHz, DMSO-*d*_*6*_): δ −76.99. HRMS (ESI) *m*/*z* calculated for C_24_H_20_ClF_3_N_3_O_5_S_2_ [M – H]^−^ 586.0480, found 586.0494.

#### (*R*)-11b-Methyl-1,2,5,6,11,11b-hexahydro-3*H*-indolizino[8,7-*b*]indol-3-one **14**

Tryptamine **12** (0.016 g, 0.1 mmol) was dissolved in a methanolic solution of **(*****S***_***a***_**)-6b** (0.0125 M, 0.2 mL). Methanol was evaporated by a stream of nitrogen. Dry 1,2-DCE (1 mL) was added. The mixture was stirred for 5 min and then lactone **13** was added. The reaction mixture was heated at 80 °C for 16 h. Then, the solvent was evaporated and the crude product was purified by column chromatography (DCM/MeOH, 500:1) to yield tetracyclic indolizinoindole **14** (0.022 g, 90%, 50% ee) as a white solid.^24^^1^H NMR (400 MHz, CDCl_3_) δ = 7.92 (s, 1H), 7.49 (d, *J* = 7.7 Hz, 1H), 7.34 (dt, *J* = 8.1, 0.9 Hz, 1H), 7.19 (ddd, *J* = 8.1, 7.1, 1.3 Hz, 1H), 7.13 (ddd, *J* = 7.8, 7.2, 1.1 Hz, 1H), 4.48 (ddd, *J* = 13.2, 5.7, 1.4 Hz, 1H), 3.17–3.00 (m, 1H), 2.93–2.75 (m, 2H), 2.74–2.63 (m, 1H), 2.53–2.40 (m, 1H), 2.29 (ddd, *J* = 11.3, 8.9, 2.3 Hz, 1H), 2.20 (t, *J* = 9.5 1H), 1.60 (s, 3H). HRMS (ESI) *m*/*z* calculated for C_15_H_17_N_2_O_1_ [M + H]^+^ 241.1335, found 241.1335, **[α]**_**D**_^**22**^ +89.4 (*c* 0.17, CHCl_3_).

## Data Availability

The data underlying this study are available in the published article and its Supporting Information.

## Supporting Information Available

^1^H, ^13^C, and NMR spectra of all prepared compounds and relevant chromatograms of compounds after optical resolution and determination of their stability; titration curves of selected derivatives; and crystallography (PDF)
