# Supplementary material for: Axially Chiral Sulfonic Acids for Brønsted Acid Catalysis: 8-Benzoimidazolylnaphthalene-1-sulfonic Acids and Their Derivatives
Source: J Org Chem. 2023 Jun 24;88(13):9265–76. doi: 10.1021/acs.joc.3c00818 (PMC10337040; doi:10.1021/acs.joc.3c00818)
Supplement: Supplementary file 1 — jo3c00818_si_001.pdf [file jo3c00818_si_001.pdf]

Axially Chiral Sulfonic Acids for Brønsted Acid Catalysis: 8-Benzoimidazolylnaphthalene-1-sulfonic Acids and Their Derivatives

Monika Tomanová,[a] Iva Vaňková,[a] Daniel Toman,[a] Adam Přibylka,[a] Ivan Nemec,[b] and Petr Cankař[a]*

[a] Department of Organic Chemistry, Faculty of Science, Palacký University
17. Listopadu 12, 771 46 Olomouc, Czech Republic
Tel. +420 585 634 437
Email: petr.cankar@upol.cz

[b] Department of Inorganic Chemistry, Faculty of Science, Palacký University
17. Listopadu 12, 771 46 Olomouc, Czech Republic

Supporting information

Table of contents

SFC Chromatograms S2

Conformational Stability of Axially Chiral Sulfonic Acid 6a S10

Pictet-Spengler Reaction of Tryptamine with α-Angelica Lactone S11

Acid-Base Titration Curves to Assign the Dissociation Constants S13

Copies of NMR Spectra S20

In-Silico Calculated pKa of Sulfonic Acid 6c S53

Crystallography S56

SFC Chromatograms

Sulfonic Acid 5b

SFC separation conditions for **5b**:
30 % MeOH + 1 % H_2O + 0.1 % DEA (diethylamin), column Chiralpak ID-3 (4.6 mm x 100 mm, 3 µm particle size), 5 min analysis. Flow rate 2.2 mL/min, column temperature 38 °C, and ABPR 2000 psi.

***rac*-5b:**

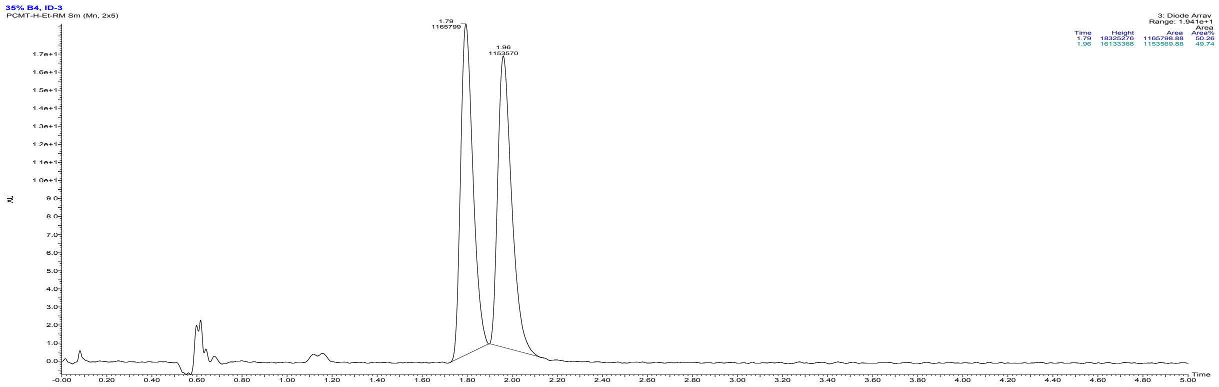

Enantiomers of **5b**:

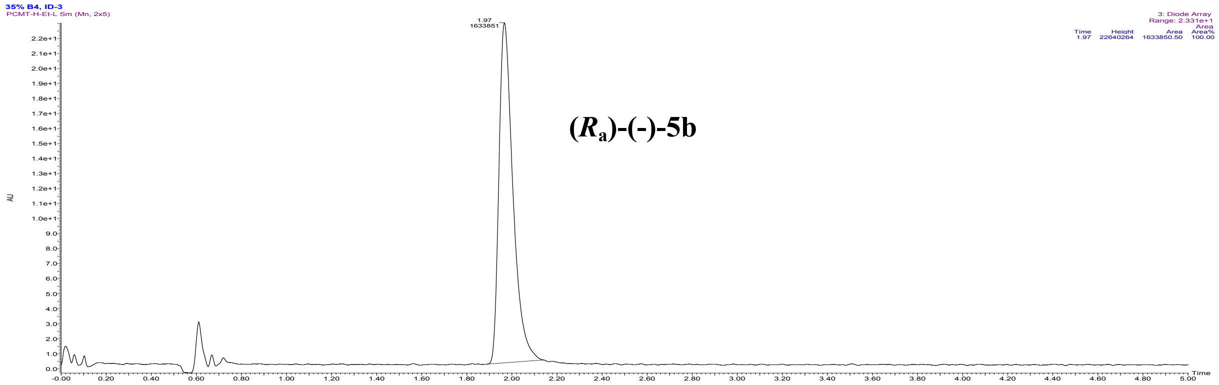

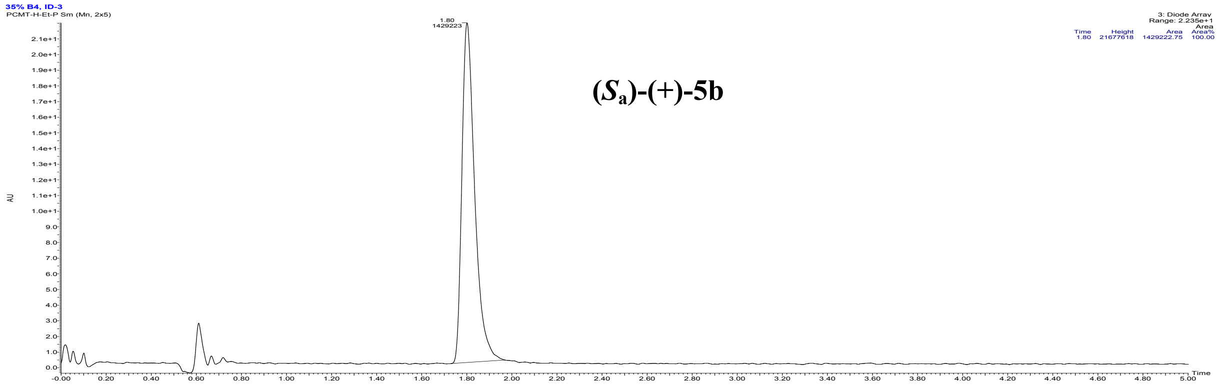

Sulfonic Acid 6a

SFC separation conditions for **6a**:
35 % MeOH + 1 % H_2O + 0.1 % DEA, column Chiralpak ID-3 (4.6 mm x 100 mm, 3 µm particle size), 5 min analysis. Flow rate 2.2 mL/min, column temperature 38 °C, and ABPR 2000 psi.

***rac*-6a**:

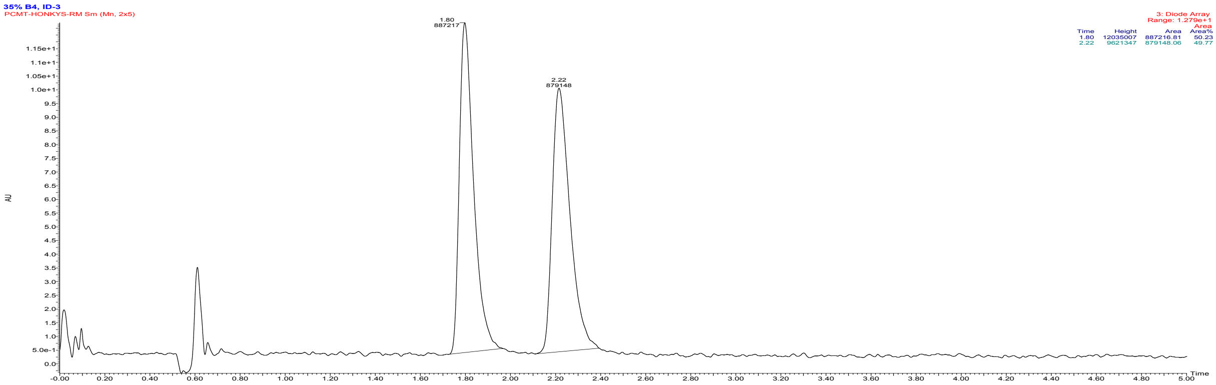

Enantiomers of **6a**:

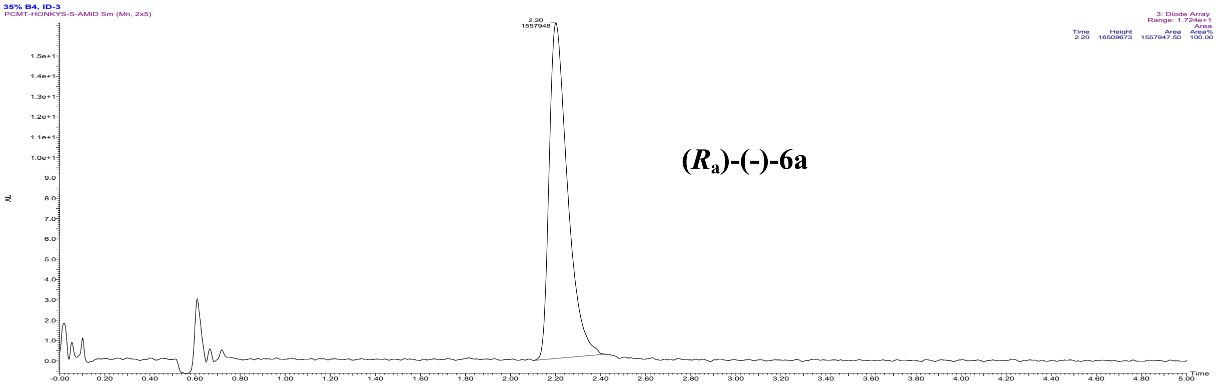

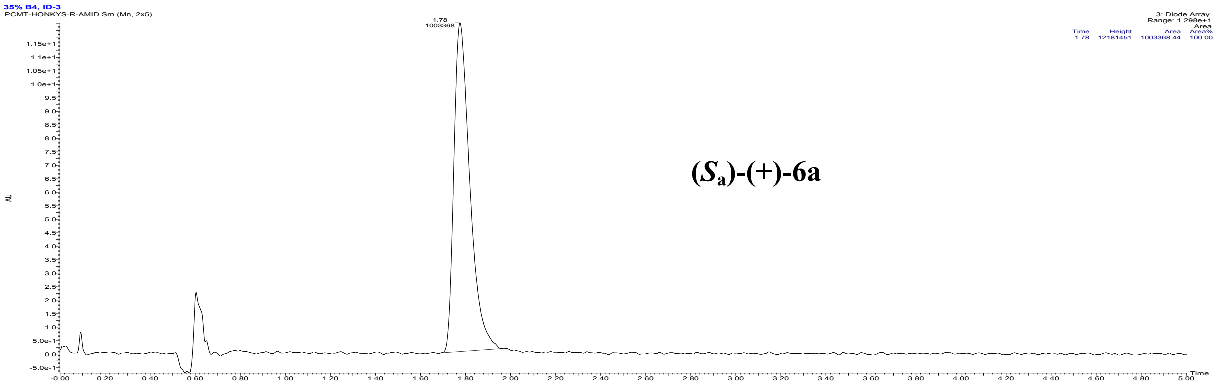

Sulfonic Acid 6b

SFC separation conditions for **6b**:
35 % MeOH + 1 % H_2O + 0.1 % DEA, column Chiralpak ID-3 (4.6 mm x 100 mm, 3 µm particle size), 5 min analysis. Flow rate 2.2 mL/min, column temperature 38 °C, and ABPR 2000 psi.

***rac*-6b**:

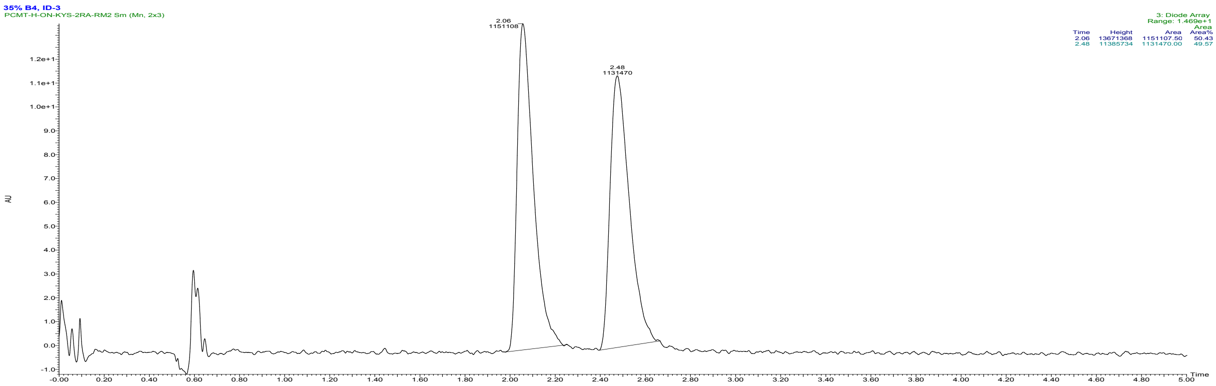

Enantiomers of **6b**:

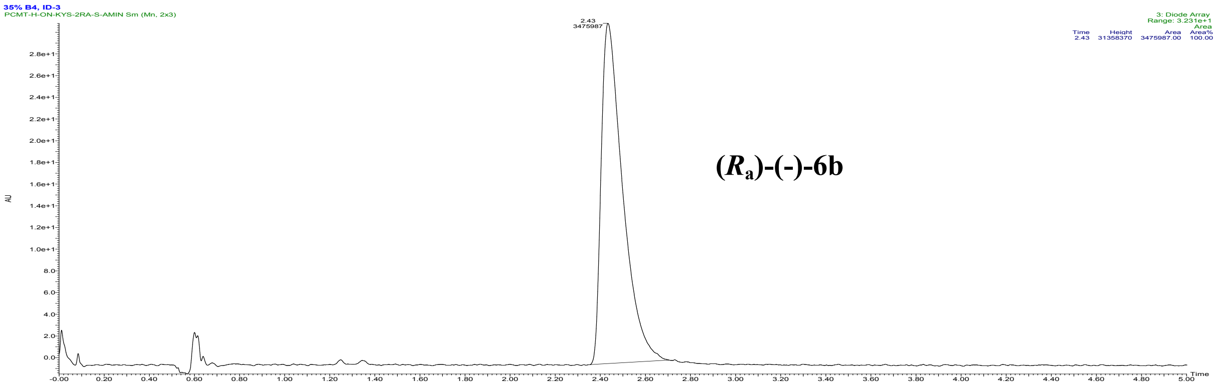

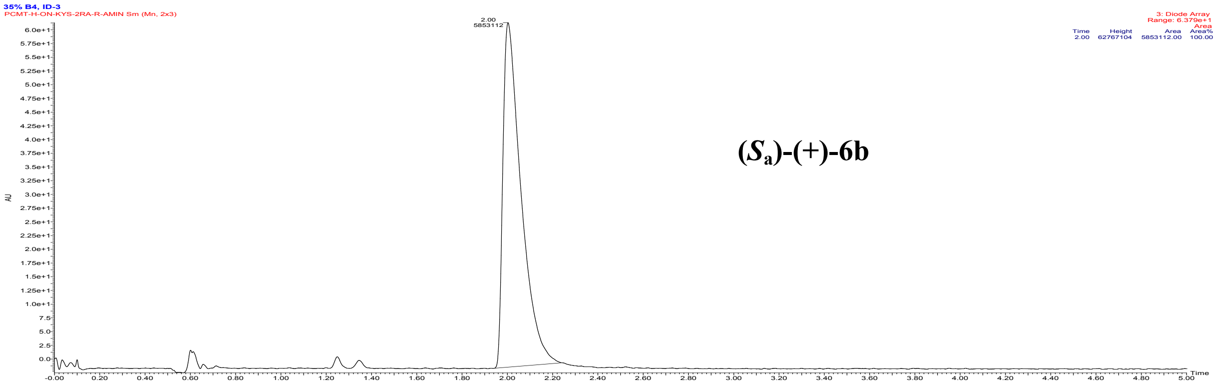

Sulfonic Acid 6c

SFC separation conditions for **6c**:
35 % MeOH + 1 % H_2O + 0.1 % DEA, column Chiralpak ID-3 (4.6 mm x 100 mm, 3 µm particle size), 5 min analysis. Flow rate 2.2 mL/min, column temperature 38 °C, and ABPR 2000 psi.

***rac*-6c**:

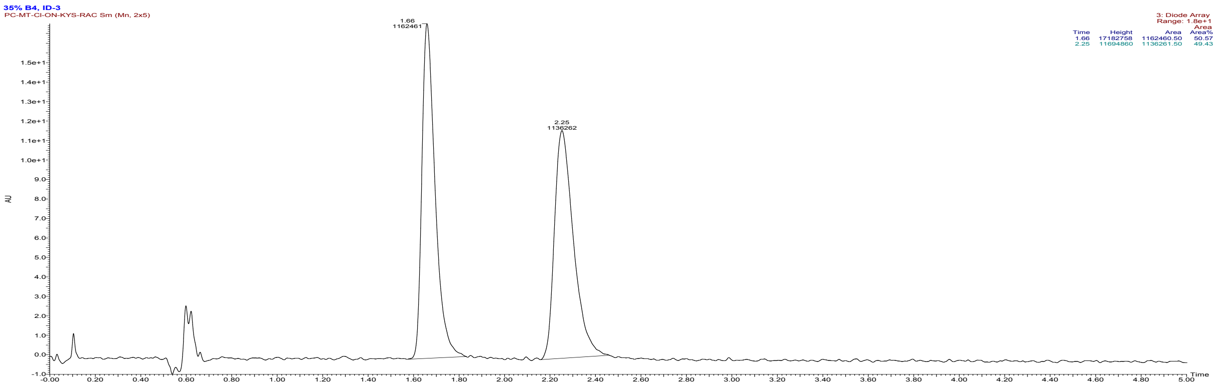

Enantiomers of **6c**:

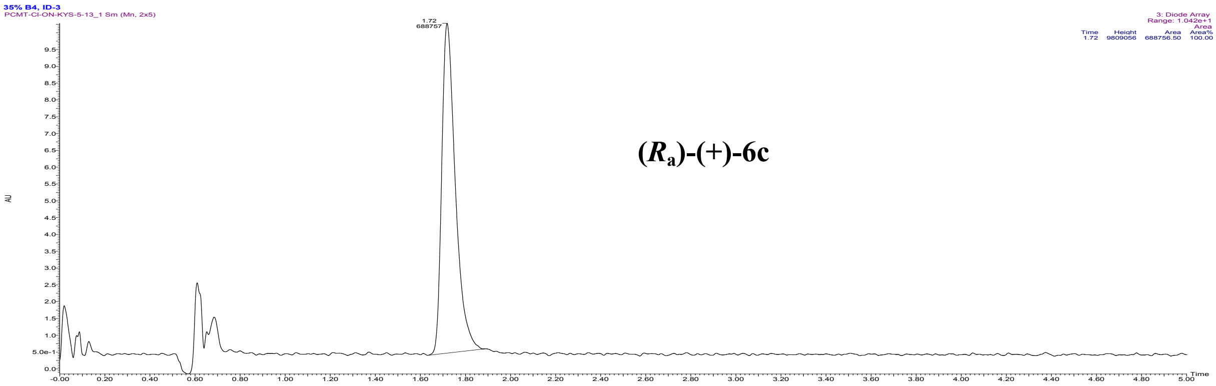

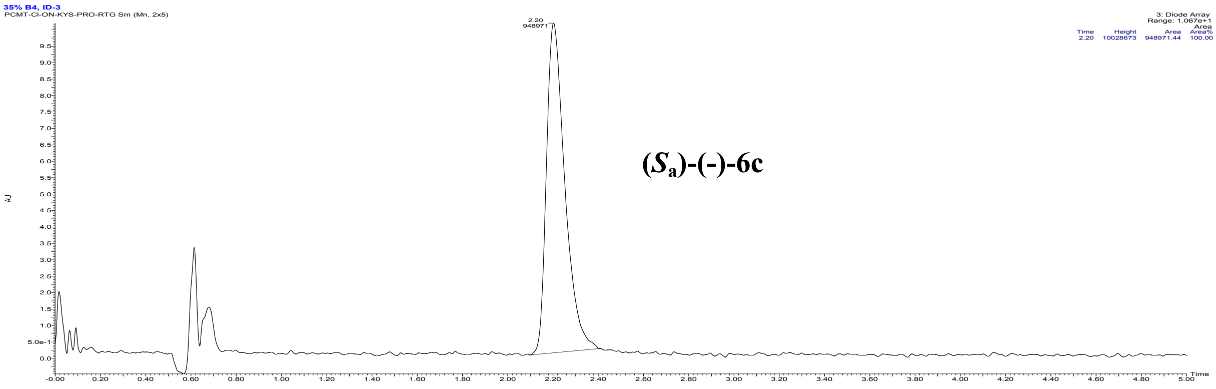

Triflic Imide 9b

SFC separation conditions for **9b**:
15 % MeOH + 1 % H_2O + 0.1 % DEA, column Chiralpak ID-3 (4.6 mm x 100 mm, 3 µm particle size), 10 min analysis. Flow rate 2.2 mL/min, column temperature 38 °C, and ABPR 2000 psi.

***rac*-9b**:

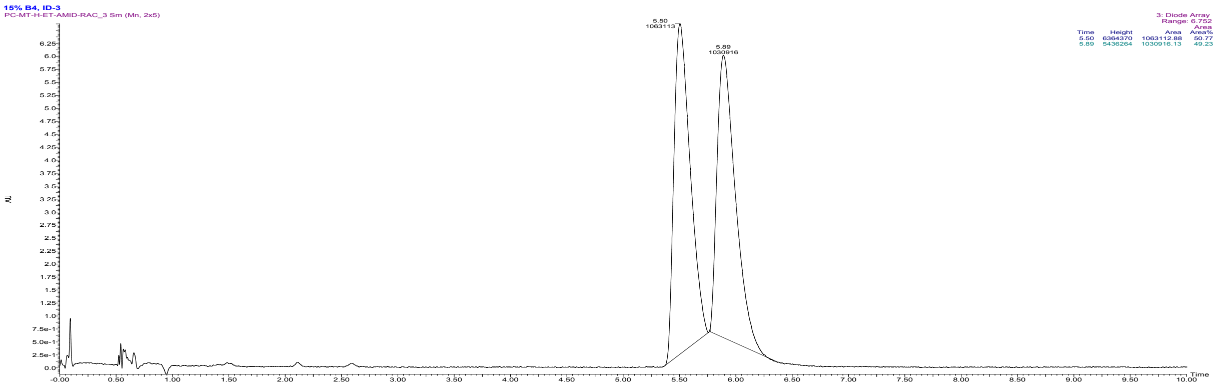

Levorotatory Enantiomer of **9b**:

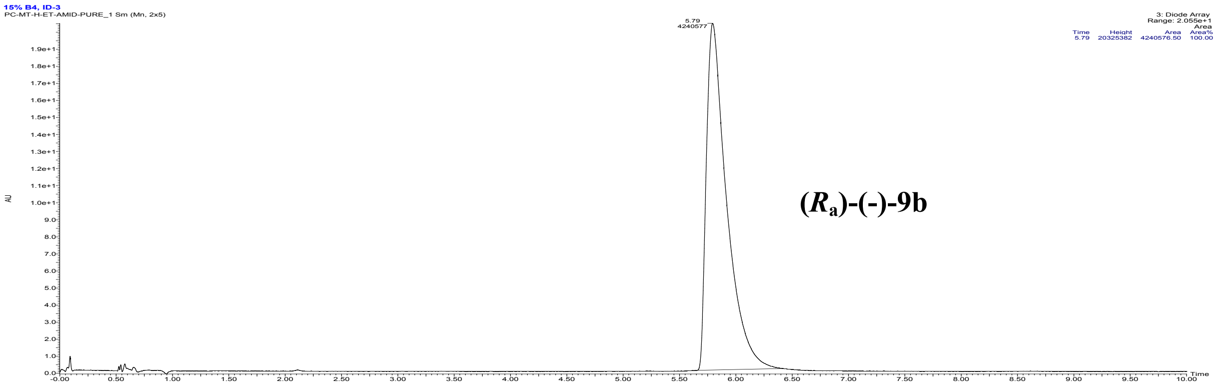

Triflic Imide 11c

SFC separation conditions for **6c**:
25% MeOH + 1 % H_2O + 0.1 % DEA, column Chiralpak ID-3 (4.6 mm x 100 mm, 3 µm particle size), 5 min analysis. Flow rate 2.2 mL/min, column temperature 38 °C, and ABPR 2000 psi.

***rac*-11c**:

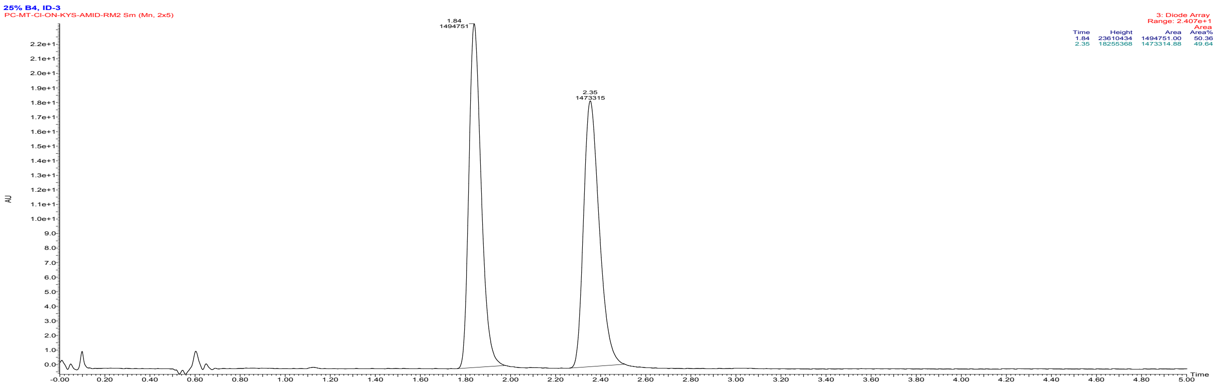

Dextrorotatory Enantiomer of **11c**:

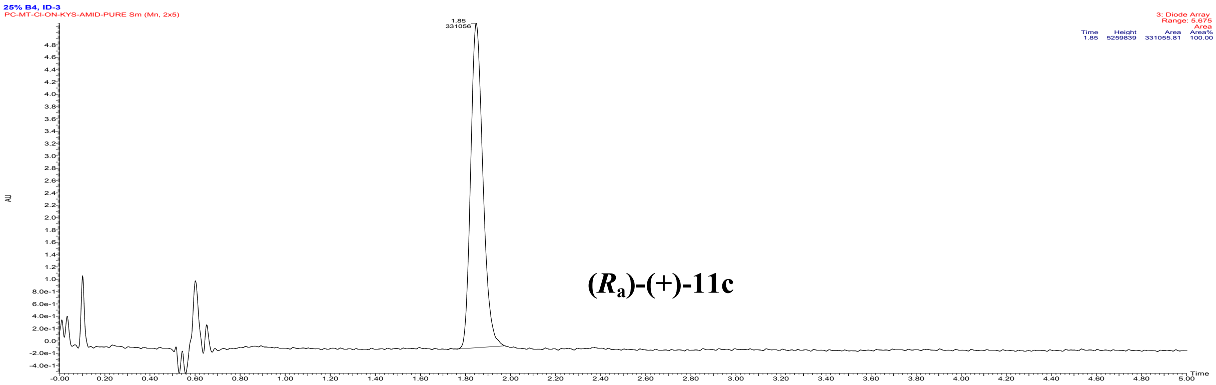

Stability of enantiomers 5b, 6a, 6b, and 6c (conditions are depicted in chromatograms):

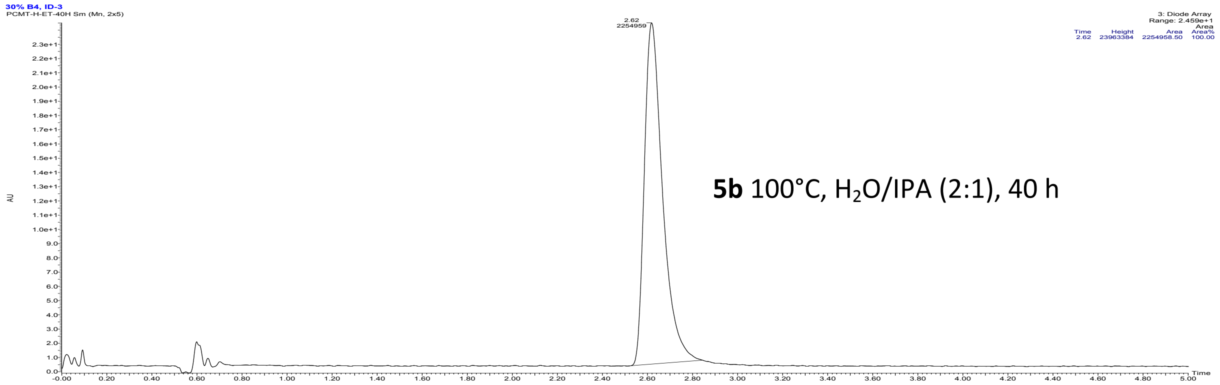

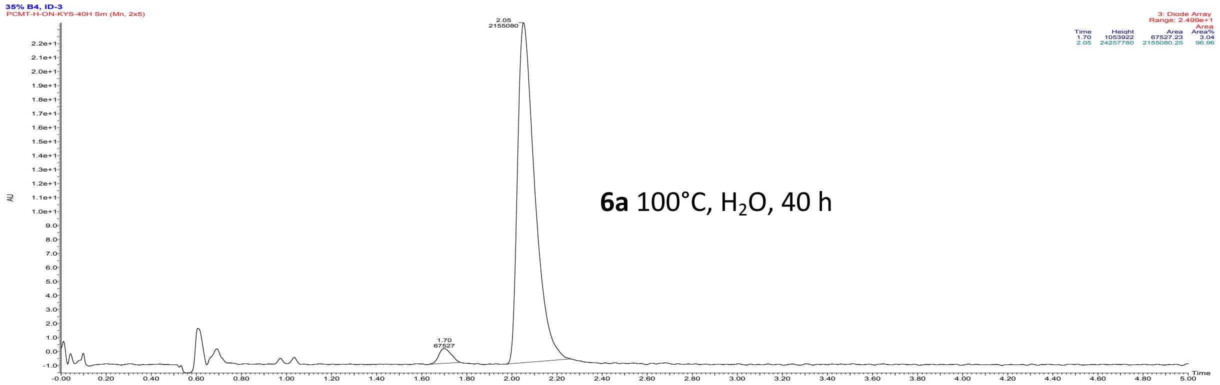

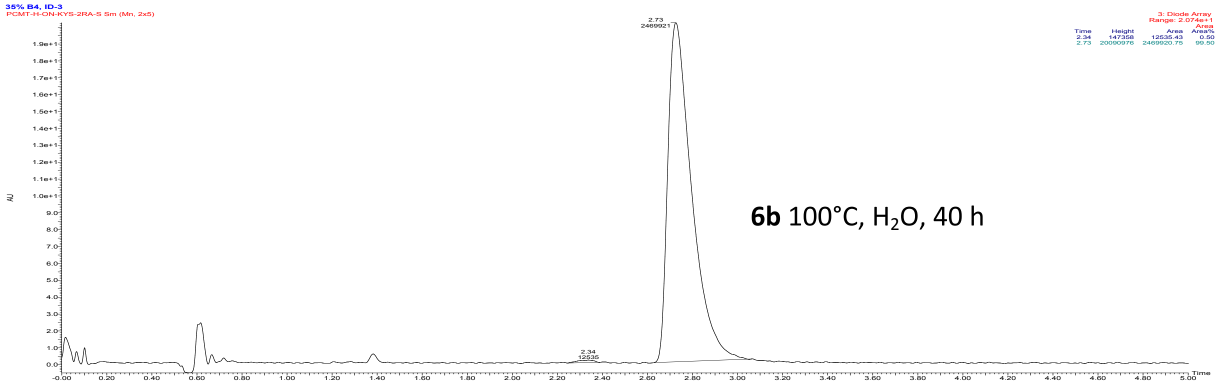

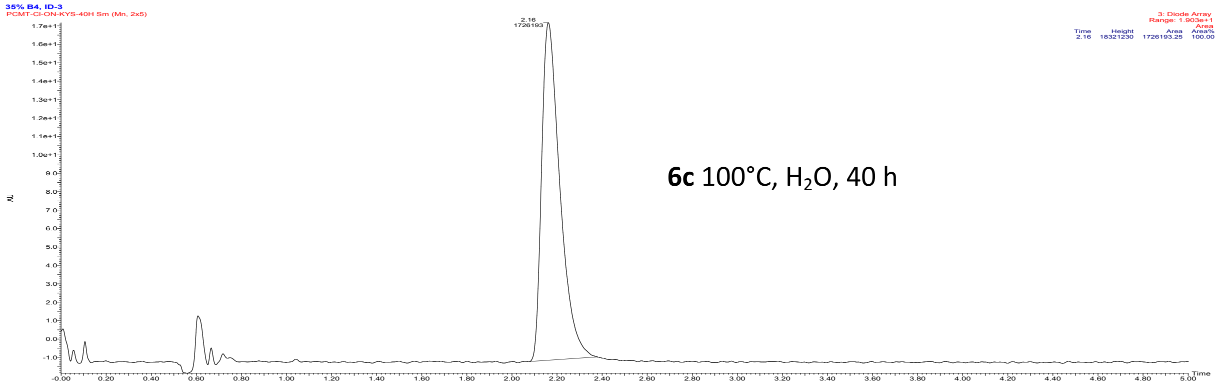

Stability of enantiomers 9b and 11c (conditions are depicted in chromatograms):

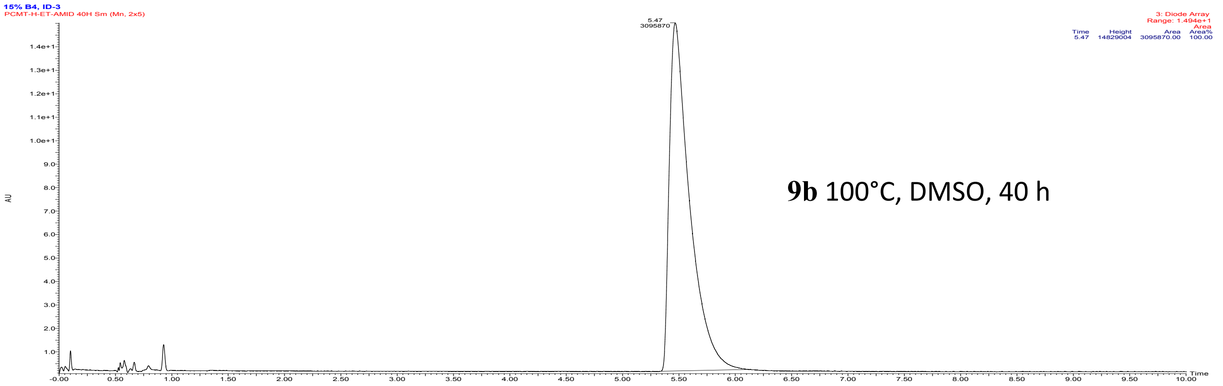

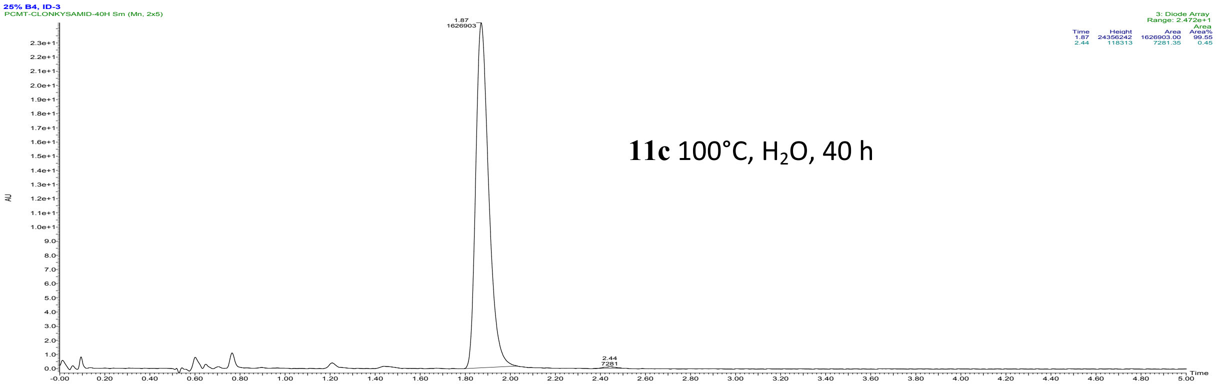

Conformational Stability of Axially Chiral Sulfonic Acid 6a

A sample of enantiomerically pure sulfonic acid **6a** (4.2 mg/0.105 mL) in water was heated in an oil bath. In time intervals, aliquots 7.5 µL were taken, frozen, lyophilized, and analyzed by chiral SFC. The rate constant of racemization (k_{rac}) was calculated from Equation 1 and subsequently, this value was used in Equation 2 to calculate the energy barrier for racemization $\Delta G^{\neq}$. The half-life of racemization was calculated according to Equation 3.

Table S1: Racemization of sulfonic acid 6a (%ee was calculated from SFC chromatograms; please see the separation conditions for 6a on page S3).

Temperature	Time (h)	%ee
100°C	0	99.99
	24	97.26
	48	92.49
	72	90.62

The rate constant of racemization (k_{rac}) was calculated by the least squares method using Equation 1:

$$\mathbf{ln}\left(\frac{\boldsymbol{R_0}}{\boldsymbol{R_0-x}}\right)=\boldsymbol{k_{rac}t} \qquad \text{(Equation 1)}$$

Where $R_0 - x = \%ee$

$k_{rac} = 4.26 \times 10^{-7}$ s^{-1}

The calculation of Gibbs activation energy $\Delta G^{\neq}$ from k_{rac} using Equation 2:

$T = 373.4$ K
$h = 6{,}626 \times 10^{-34}$ J × s *(Planck constant)*
$k_B = 1.38 \times 10^{-23}$ J × K^{-1}*(Boltzman constant)*
$R = 8.3145$ *(gas constant)*
κ = transmission coefficient (equals to 1)

$$\Delta\boldsymbol{G}^{\neq} = -\boldsymbol{RTln}\left(\frac{\boldsymbol{hk_{rac}}}{\boldsymbol{\kappa T k_B}}\right) \qquad \text{(Equation 2)}$$

$\Delta G^{\neq} = 137.7$ kJ × mol^{-1} (32.9 kcal)

The calculation of the half-life of racemization using Equation 3:

$$\boldsymbol{\tau_{1/2}} = \frac{\mathbf{ln}\ (2)}{\boldsymbol{k_{rac}}} \qquad \text{(Equation 3)}$$

$\tau_{1/2}^{T=373.4} = 19\ days$

$\tau_{1/2}^{T=310.5} = 530\ years$

Pictet-Spengler Reaction of Tryptamine with α-Angelica Lactone

The values of enantiomeric excess were determined by the SFC method and put in Table 1 in the article.

The SFC separation conditions for tetracyclic heterocycle **14**: 15% MeOH + 1% H_2O + 0,1 % DEA, column Chiralpak ID-3 (4.6 mm x 100 mm, 3 µm particle size), 5 min analysis. Flow rate 2.2 mL/min, column temperature 38 °C, and ABPR 2000 psi.

Entry 1

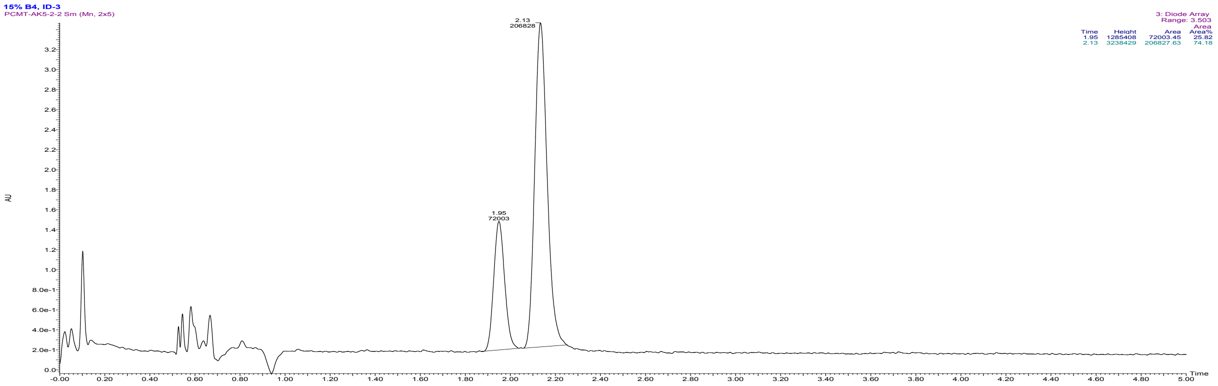

Entry 2

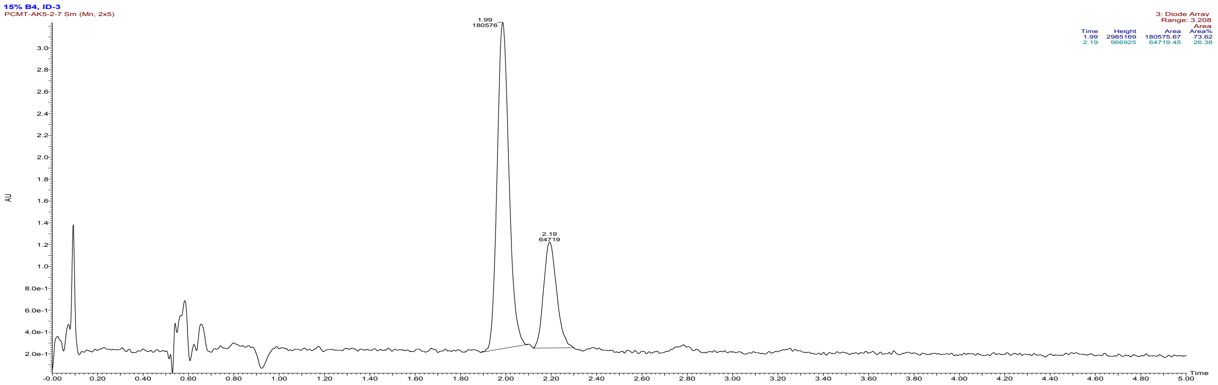

Entry 3

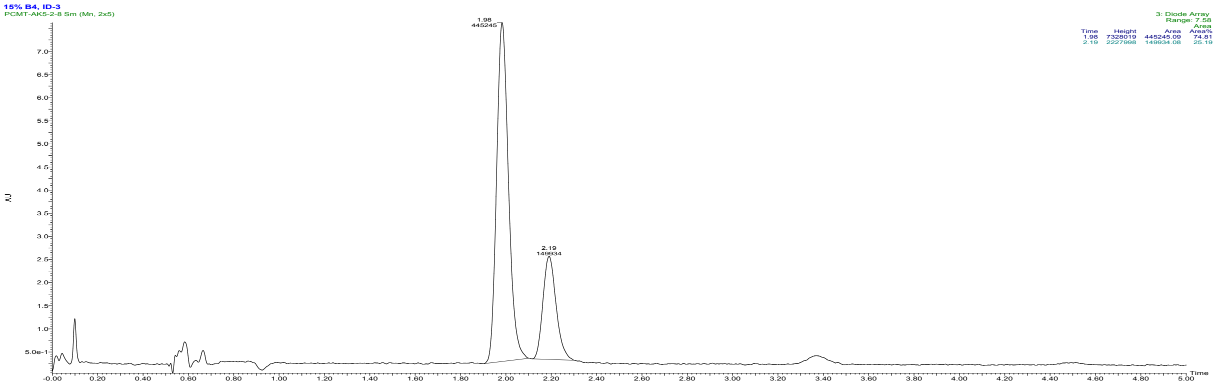

Entry 4

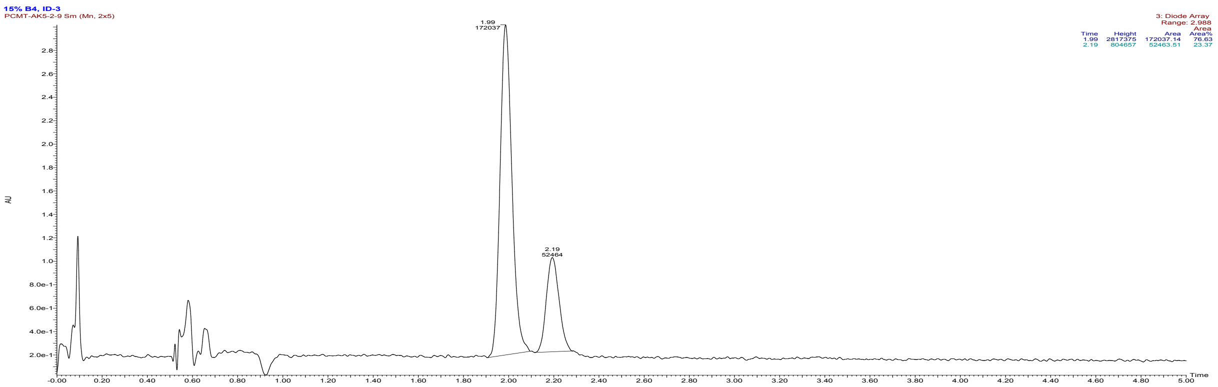

Entry 5

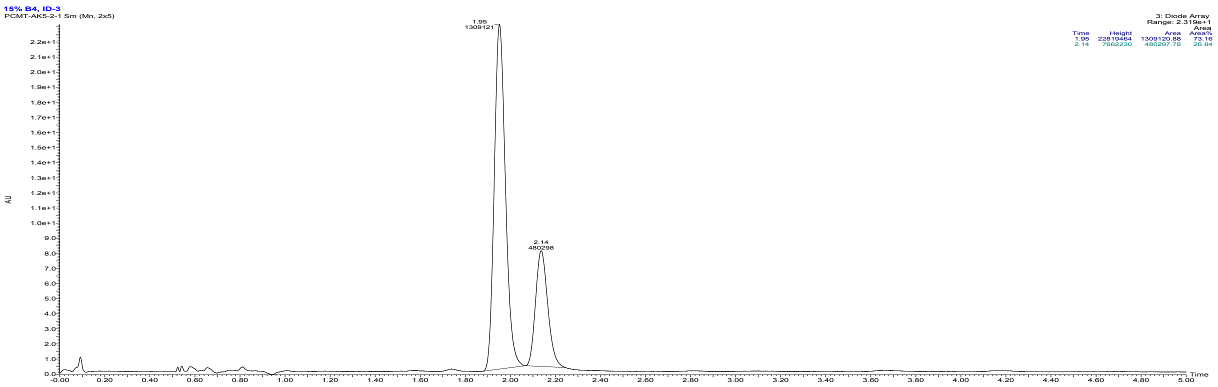

Acid-Base Titration Curves to Assign the Dissociation Constants

Sulfonic Acid 5e

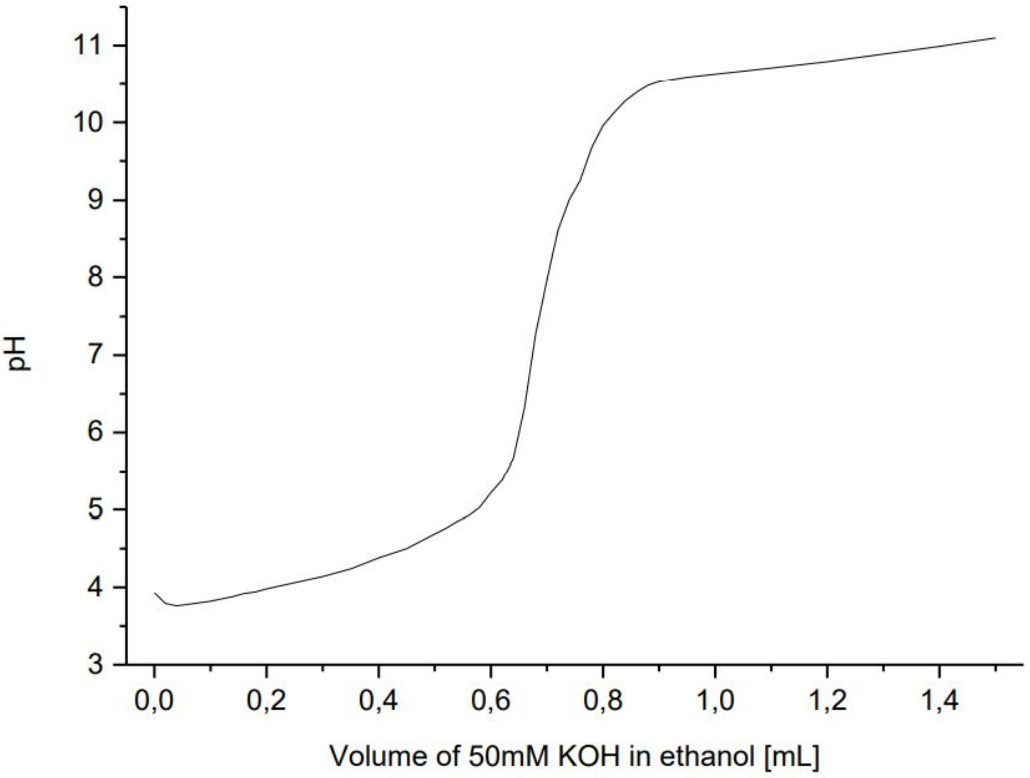

Table S2: Titration data of sulfonic acid 5e used for *pKₐ* determination.

Sulfonic Acid 5e					
Volume of 50 mM KOH [mL]	pH	Volume of 50 mM KOH [mL]	pH	Volume of 50 mM KOH [mL]	pH
0	3,93	0,5	4,69	0,82	10,13
0,02	3,79	0,52	4,76	0,84	10,28
0,04	3,76	0,54	4,85	0,86	10,39
0,06	3,78	0,56	4,92	0,88	10,48
0,08	3,8	0,58	5,03	0,9	10,53
0,1	3,82	0,6	5,22	0,95	10,59
0,12	3,85	0,62	5,38	1	10,63
0,14	3,88	0,64	5,66	1,05	10,67
0,16	3,92	0,66	6,32	1,1	10,71
0,18	3,94	0,68	7,28	1,2	10,79
0,2	3,98	0,7	7,96	1,3	10,89
0,25	4,06	0,72	8,62	1,4	10,99
0,3	4,14	0,74	9,01	1,5	11,1
0,35	4,24	0,76	9,27		
0,4	4,38	0,78	9,68		
0,45	4,5	0,8	9,96		

Sulfonic Acid 6c

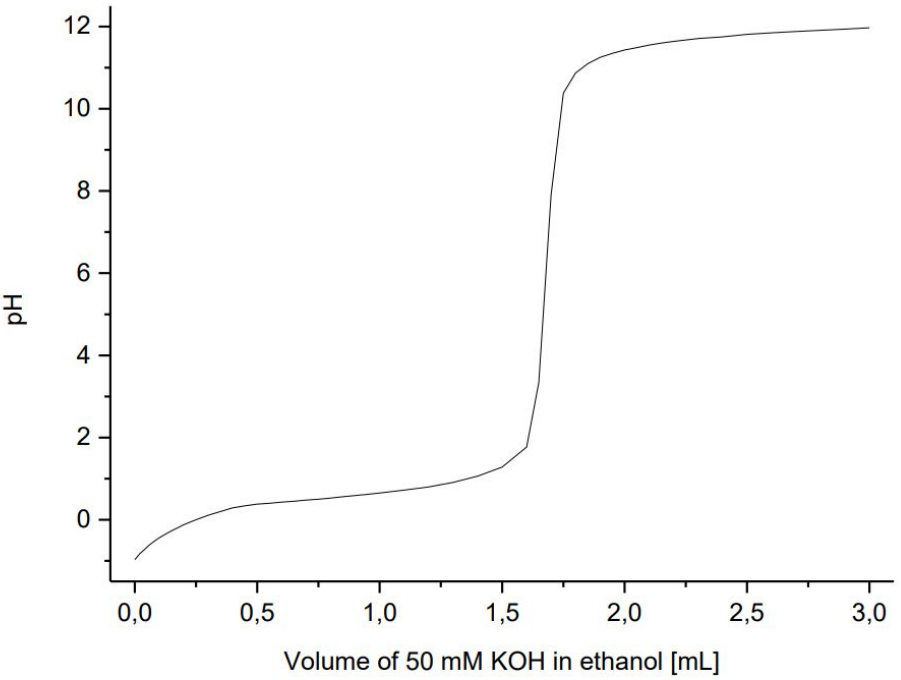

Table S3: Titration data of sulfonic acid 6c used for *pK*$_a$ determination.

Sulfonic Acid 6c							
Volume of 50 mM KOH [mL]	pH	Volume of 50 mM KOH [mL]	pH	Volume of 50 mM KOH [mL]	pH	Volume of 50 mM KOH [mL]	pH
0	-0,97	0,4	0,29	1,2	0,8	2,15	11,6
0,02	-0,83	0,45	0,34	1,4	1,06	1,3	0,91
0,04	-0,72	0,5	0,38	1,5	1,28	2,2	11,64
0,06	-0,61	0,55	0,4	1,6	1,77	2,3	11,71
0,08	-0,52	0,6	0,43	1,65	3,34	2,4	11,75
0,1	-0,44	0,65	0,45	1,7	7,91	2,5	11,81
0,12	-0,37	0,7	0,48	1,75	10,38	2,6	11,85
0,14	-0,3	0,75	0,5	1,8	10,87	2,7	11,88
0,16	-0,24	0,8	0,53	1,85	11,1	2,8	11,91
0,18	-0,18	0,85	0,56	1,9	11,25	2,9	11,94
0,2	-0,12	0,9	0,59	1,95	11,35	3	11,97
0,25	0	0,95	0,62	2	11,43		
0,3	0,11	1	0,65	2,05	11,49		
0,35	0,2	1,11	0,73	2,1	11,55		

Triflic Imide 9b

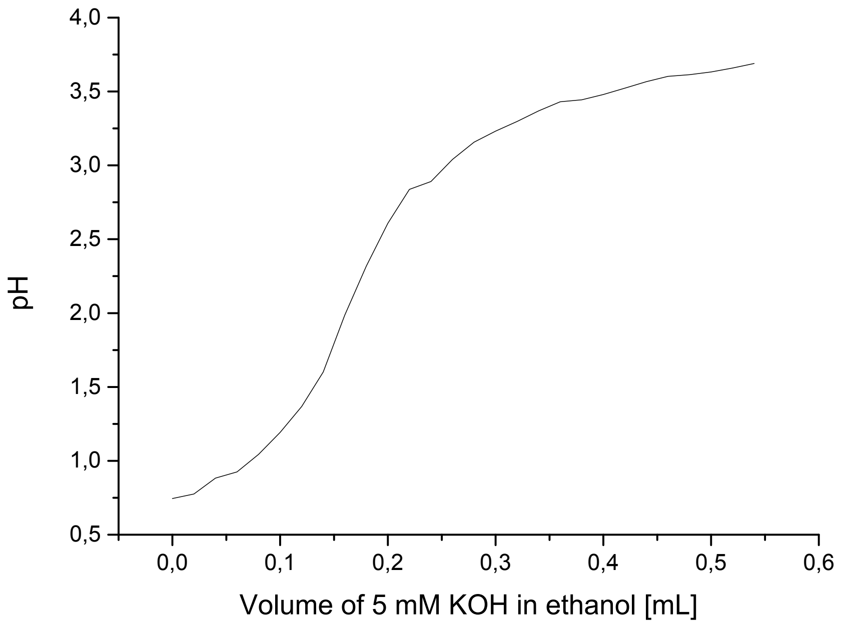

Table S4: Titration data of sulfonic acid 9b used for *pK_a* determination.

Triflic Imide 9b					
Volume of 5 mM KOH [mL]	pH	Volume of 5 mM KOH [mL]	pH	Volume of 5 mM KOH [mL]	pH
0	0,745	0,28	3,157	0,56	3,711
0,02	0,776	0,3	3,232	0,58	3,739
0,04	0,883	0,32	3,297	0,6	3,762
0,06	0,925	0,34	3,369	0,62	3,776
0,08	1,044	0,36	3,43	0,64	3,797
0,1	1,192	0,38	3,444	0,66	3,814
0,12	1,368	0,4	3,48	0,68	3,834
0,14	1,601	0,42	3,523	0,7	3,85
0,16	1,986	0,44	3,567	0,72	3,876
0,18	2,318	0,46	3,603	0,74	3,881
0,2	2,608	0,48	3,614	0,76	3,896
0,22	2,837	0,5	3,632	0,78	3,912
0,24	2,891	0,52	3,659	0,8	3,916
0,26	3,04	0,54	3,69		

Triflic Imide 11a

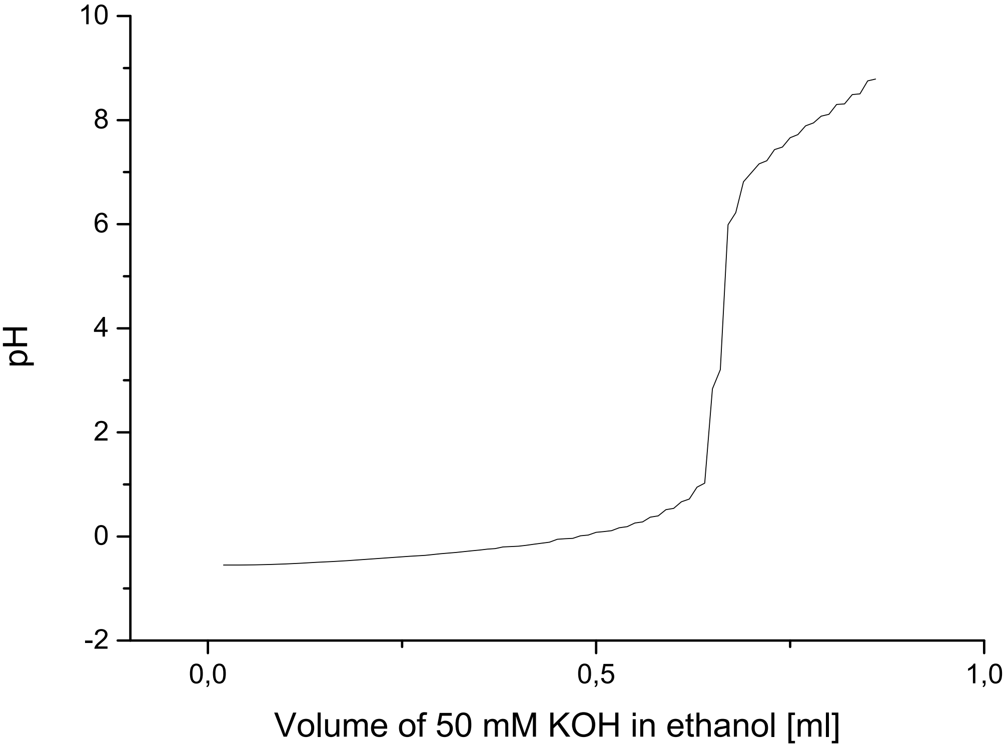

Table S5: Titration data of sulfonic acid 11a used for pK_a determination.

Triflic Imide 11a							
Volume of 50 mM KOH [mL]	pH	Volume of 50 mM KOH [mL]	pH	Volume of 50 mM KOH [mL]	pH	Volume of 50 mM KOH [mL]	pH
0,02	-0,551	0,36	-0,243	0,55	0,258	0,74	7,483
0,04	-0,549	0,37	-0,235	0,56	0,28	0,75	7,66
0,06	-0,546	0,38	-0,201	0,57	0,369	0,76	7,72
0,08	-0,539	0,39	-0,195	0,58	0,395	0,77	7,89
0,1	-0,527	0,4	-0,188	0,59	0,516	0,78	7,946
0,12	-0,514	0,41	-0,169	0,6	0,54	0,79	8,076
0,14	-0,498	0,42	-0,149	0,61	0,665	0,8	8,111
0,16	-0,483	0,44	-0,111	0,62	0,718	0,81	8,301
0,18	-0,465	0,45	-0,056	0,63	0,946	0,82	8,312
0,2	-0,443	0,46	-0,044	0,64	1,024	0,83	8,49
0,22	-0,424	0,47	-0,038	0,65	2,836	0,84	8,505
0,24	-0,403	0,48	0,011	0,66	3,204	0,85	8,753
0,26	-0,38	0,49	0,026	0,67	5,989	0,86	8,787
0,28	-0,362	0,5	0,08	0,68	6,22		
0,3	-0,331	0,51	0,094	0,69	6,814		
0,32	-0,307	0,52	0,11	0,71	7,158		
0,34	-0,274	0,53	0,166	0,72	7,217		
0,35	-0,263	0,54	0,189	0,73	7,433		

Triflic Imide 11c

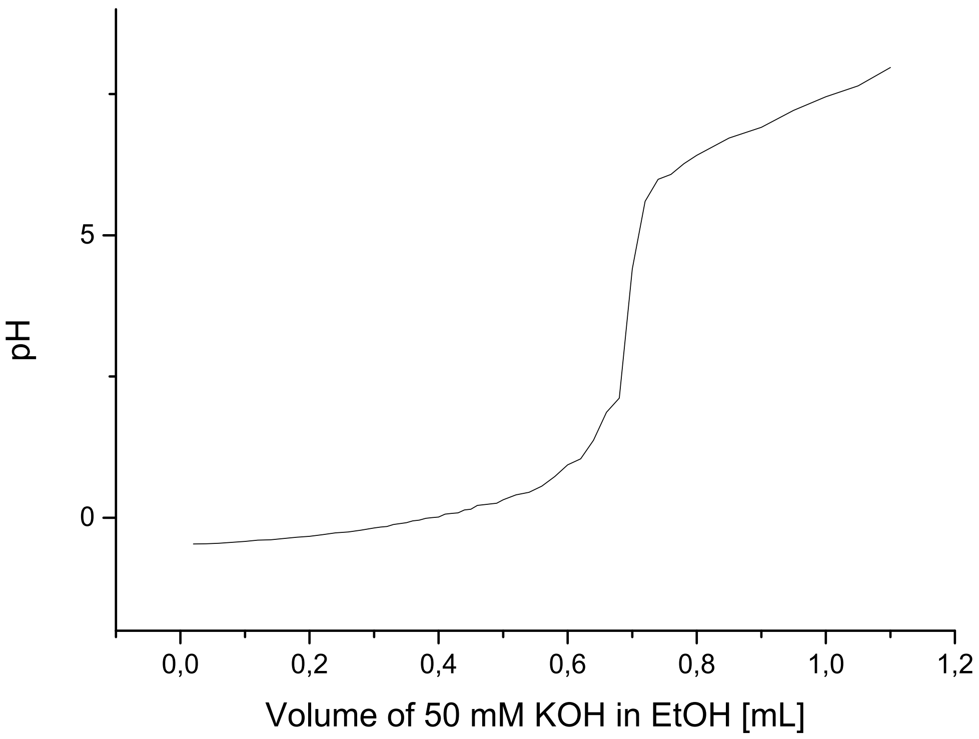

Table S6: Titration data of sulfonic acid 11c used for *pK_a* determination.

Triflic Imide 11c					
Volume of 50 mM KOH [mL]	pH	Volume of 50 mM KOH [mL]	pH	Volume of 50 mM KOH [mL]	pH
0,02	-0,466	0,35	-0,089	0,58	0,729
0,04	-0,46	0,36	-0,054	0,6	0,936
0,06	-0,45	0,37	-0,044	0,62	1,041
0,08	-0,436	0,38	-0,009	0,64	1,37
0,1	-0,419	0,39	0,001	0,66	1,865
0,12	-0,397	0,4	0,011	0,68	2,116
0,14	-0,389	0,41	0,063	0,7	4,402
0,16	-0,367	0,42	0,075	0,72	5,6
0,18	-0,345	0,43	0,085	0,74	5,99
0,2	-0,33	0,44	0,139	0,76	6,078
0,22	-0,299	0,45	0,152	0,78	6,266
0,24	-0,268	0,46	0,218	0,8	6,416
0,26	-0,253	0,47	0,231	0,85	6,722
0,28	-0,219	0,48	0,245	0,9	6,91
0,3	-0,181	0,49	0,257	0,95	7,211
0,31	-0,165	0,5	0,317	1	7,453
0,32	-0,157	0,52	0,404	1,05	7,645
0,33	-0,121	0,54	0,449	1,1	7,971
0,34	-0,103	0,56	0,558		

BINOL-Phosphoric Acid

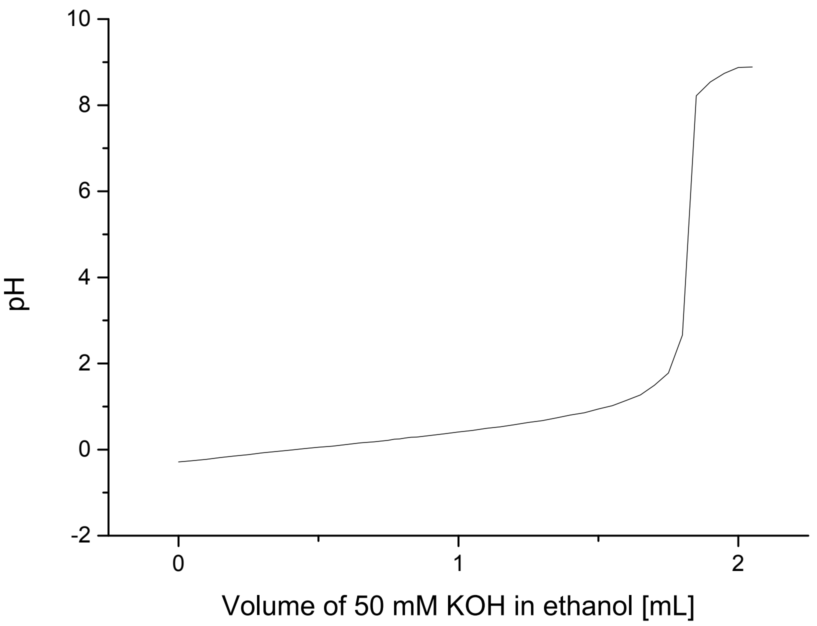

Table S7: Titration data of BINOL-phosphoric acid used for *pK_a* determination.

BINOL-Phosphoric Acid					
Volume of 50 mM KOH [mL]	pH	Volume of 50 mM KOH [mL]	pH	Volume of 50 mM KOH [mL]	pH
0	-0,285	0,75	0,217	1,4	0,801
0,02	-0,275	0,77	0,24	1,45	0,856
0,05	-0,259	0,79	0,246	1,5	0,944
0,1	-0,225	0,81	0,268	1,55	1,02
0,15	-0,184	0,83	0,286	1,6	1,142
0,2	-0,149	0,85	0,291	1,65	1,268
0,25	-0,119	0,9	0,329	1,7	1,493
0,3	-0,075	0,95	0,366	1,75	1,779
0,35	-0,045	1	0,408	1,8	2,661
0,4	-0,013	1,05	0,445	1,85	8,219
0,45	0,022	1,1	0,495	1,9	8,539
0,5	0,054	1,15	0,529	1,95	8,74
0,55	0,08	1,2	0,576	2	8,876
0,6	0,119	1,25	0,63	2,05	8,887
0,65	0,156	1,3	0,672		
0,7	0,182	1,35	0,737		

***p*-Toluene Sulfonic Acid**

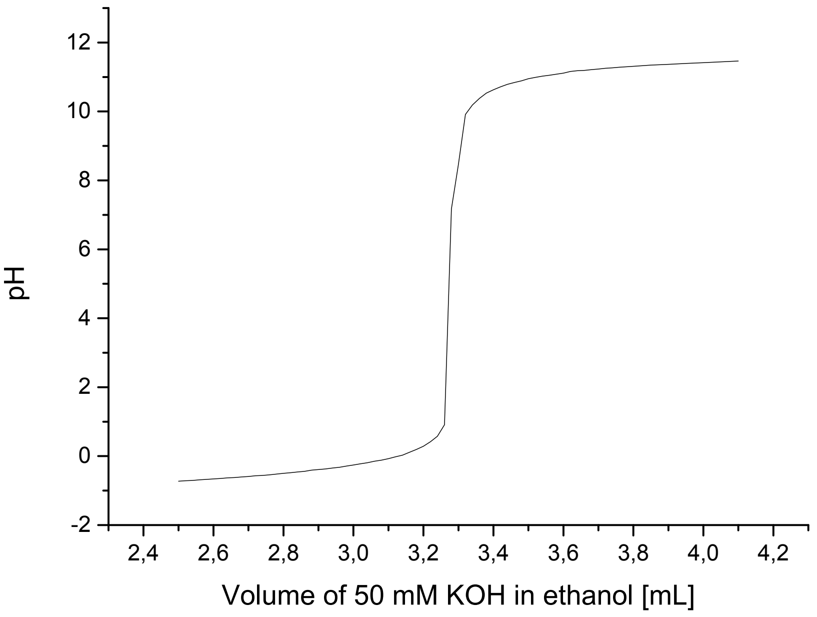

Table S8: Titration data of *p*-toluene sulfonic acid used for *pK_a* determination.

***p*-Toluene Sulfonic Acid**					
Volume of 50 mM KOH [mL]	pH	Volume of 50 mM KOH [mL]	pH	Volume of 50 mM KOH [mL]	pH
2,5	-0,729	2,98	-0,286	3,46	10,842
2,52	-0,713	3	-0,256	3,48	10,889
2,54	-0,703	3,02	-0,22	3,5	10,95
2,56	-0,687	3,04	-0,193	3,52	10,99
2,58	-0,675	3,06	-0,146	3,54	11,025
2,6	-0,659	3,08	-0,117	3,56	11,053
2,62	-0,647	3,1	-0,073	3,58	11,08
2,64	-0,632	3,12	-0,02	3,6	11,111
2,66	-0,62	3,14	0,027	3,62	11,16
2,68	-0,606	3,16	0,11	3,64	11,181
2,7	-0,589	3,18	0,196	3,66	11,192
2,72	-0,57	3,2	0,286	3,68	11,215
2,74	-0,558	3,22	0,419	3,7	11,232
2,76	-0,541	3,24	0,578	3,72	11,252
2,78	-0,522	3,26	0,91	3,74	11,265
2,8	-0,5	3,28	7,175	3,76	11,283
2,82	-0,481	3,3	8,454	3,78	11,295
2,84	-0,459	3,32	9,916	3,8	11,308
2,86	-0,442	3,34	10,184	3,85	11,343
2,88	-0,408	3,36	10,38	3,9	11,365
2,9	-0,39	3,38	10,531	3,95	11,393
2,92	-0,371	3,4	10,628	4	11,415
2,94	-0,346	3,42	10,712	4,1	11,465
2,96	-0,325	3,44	10,788		

Copies of NMR Spectra

8-((2-Nitrophenyl)amino)naphthalene-1-sulfonic acid 2a

^{1}H NMR (400 MHz, DMSO-d_6)

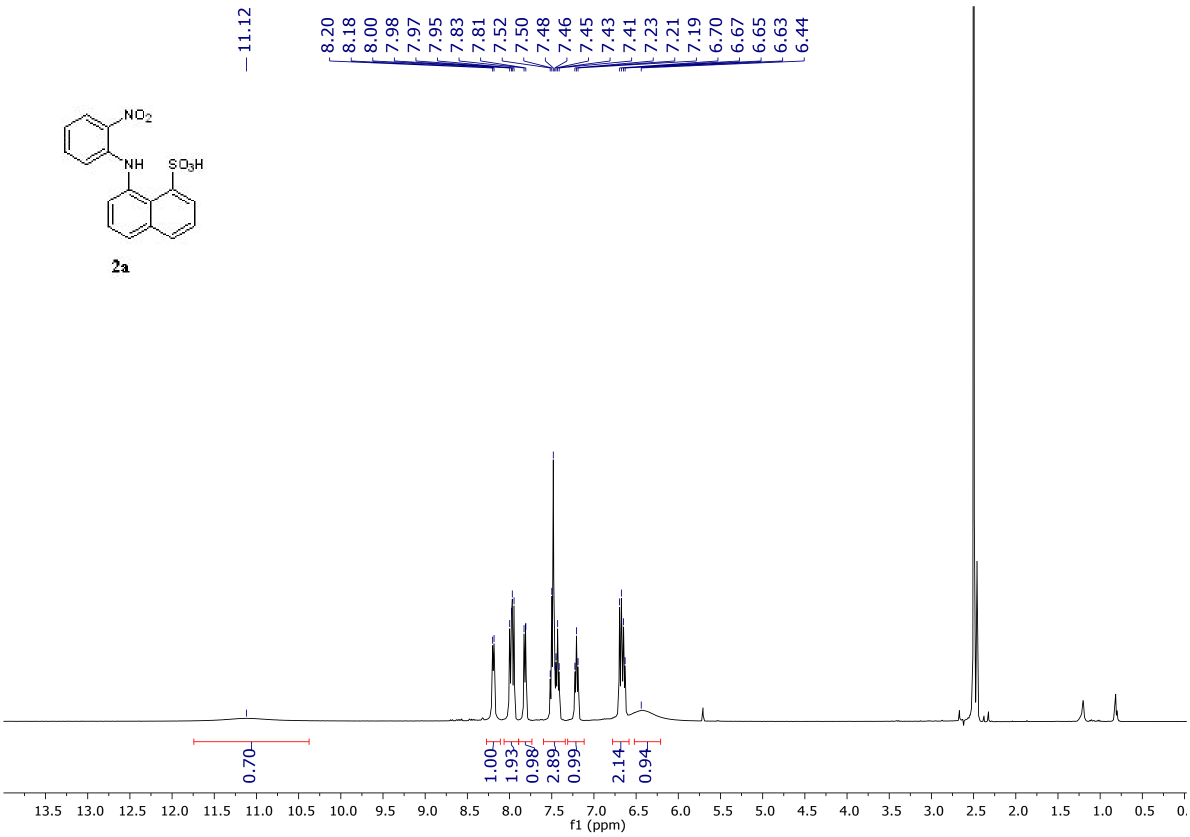

$^{13}C\{^{1}H\}$ NMR (101 MHz, DMSO-d_6)

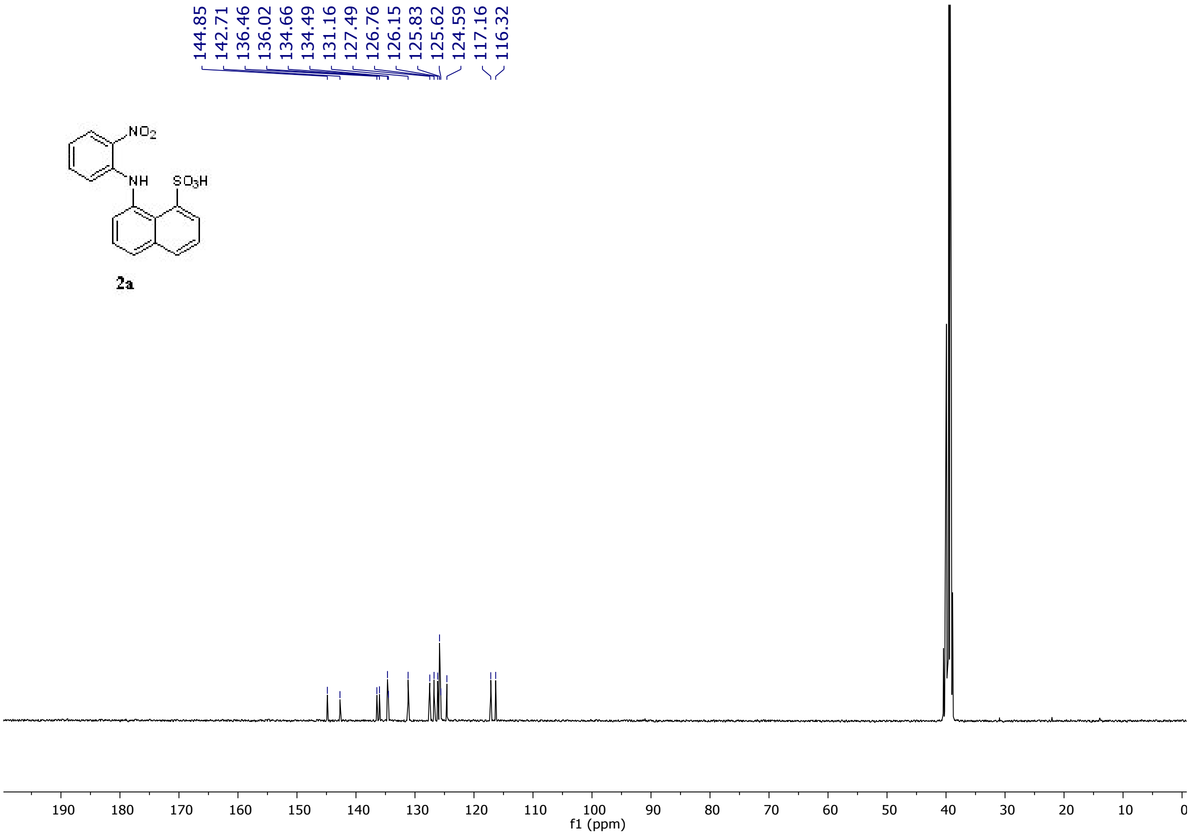

8-((2-Chloro-6-nitrophenyl)amino)naphthalene-1-sulfonic acid 2b

^{1}H NMR (500 MHz, DMSO-d_6)

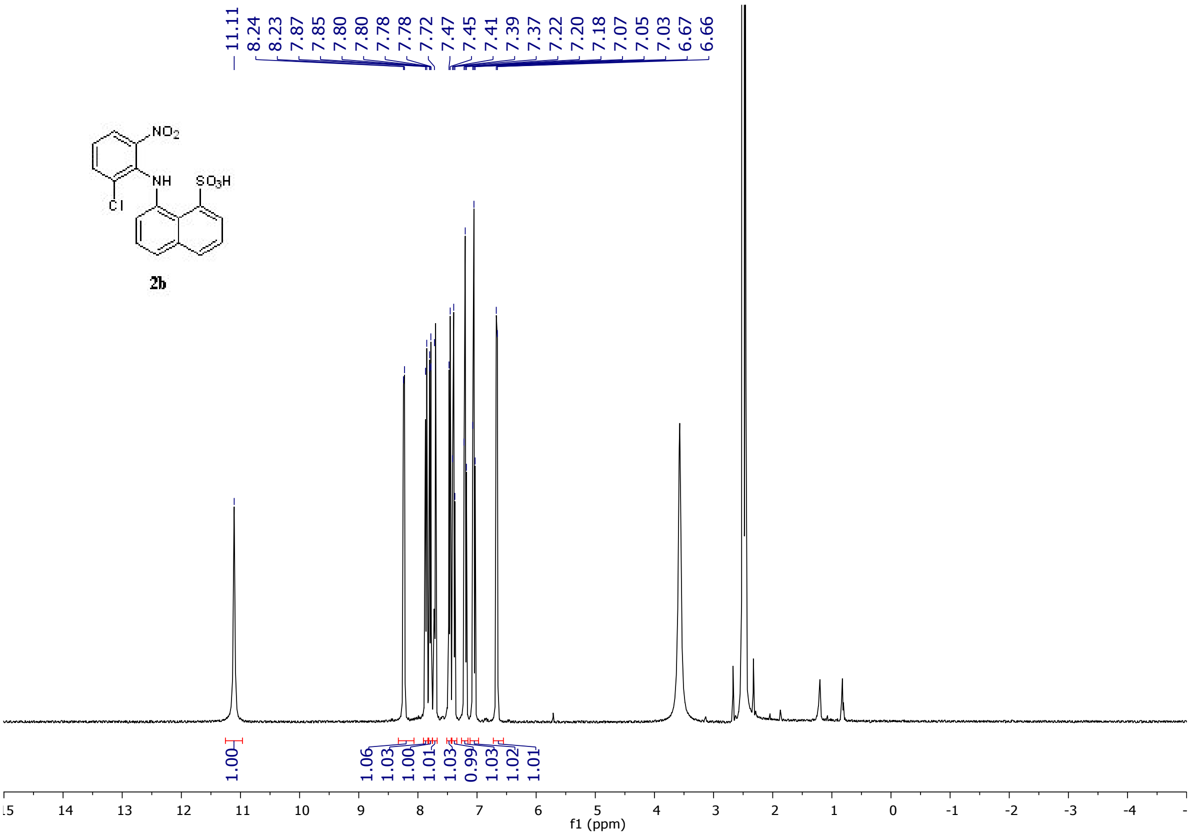

^{13}C{^{1}H} NMR (101 MHz, DMSO-d_6)

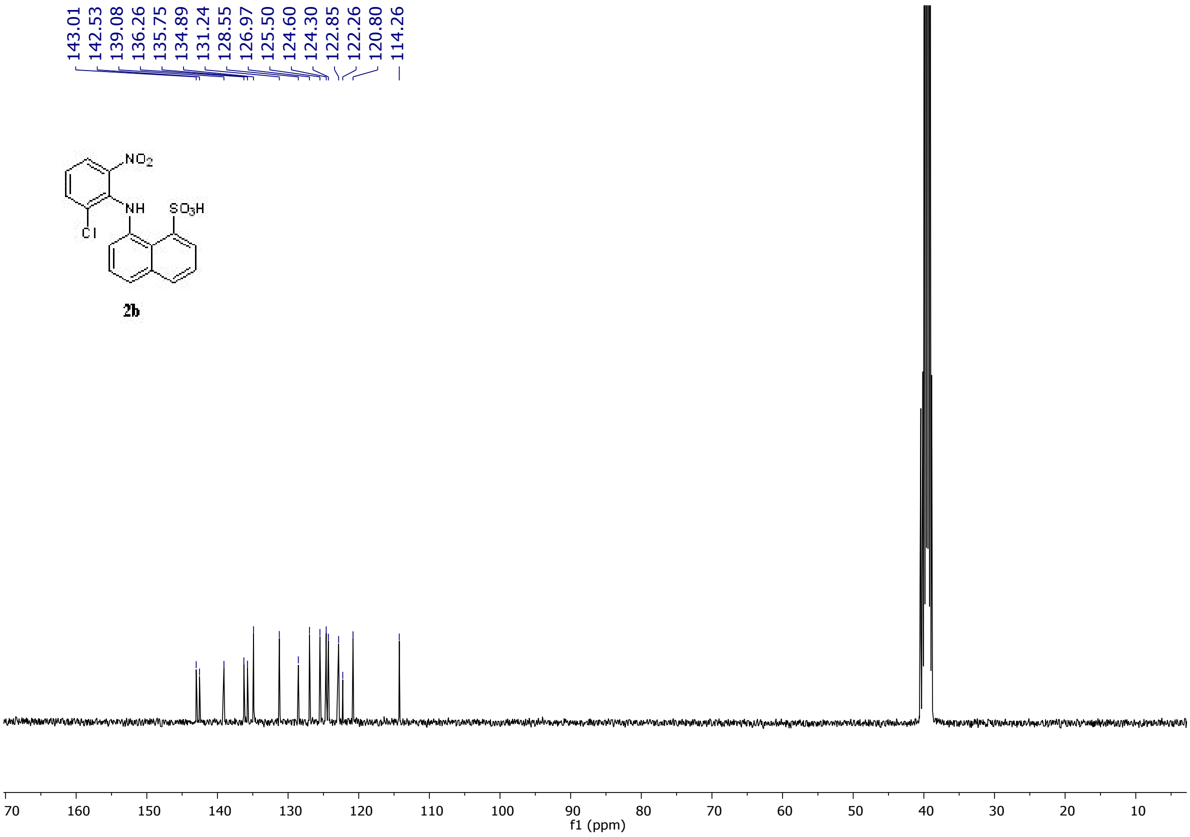

8-((2-Aminophenyl)amino)naphthalene-1-sulfonic acid 3a

^{1}H NMR (400 MHz, DMSO-d_6)

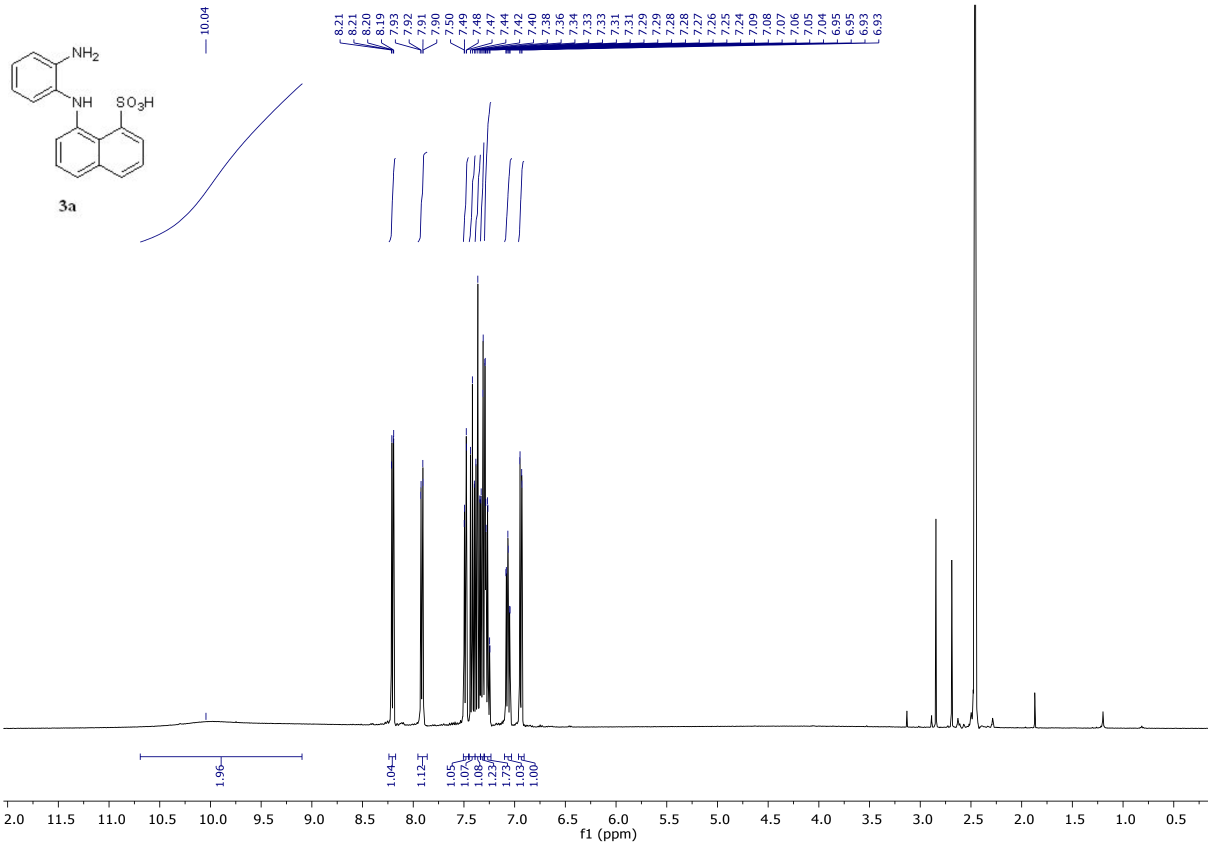

^{13}C{^{1}H} NMR (101 MHz, DMSO-d_6)

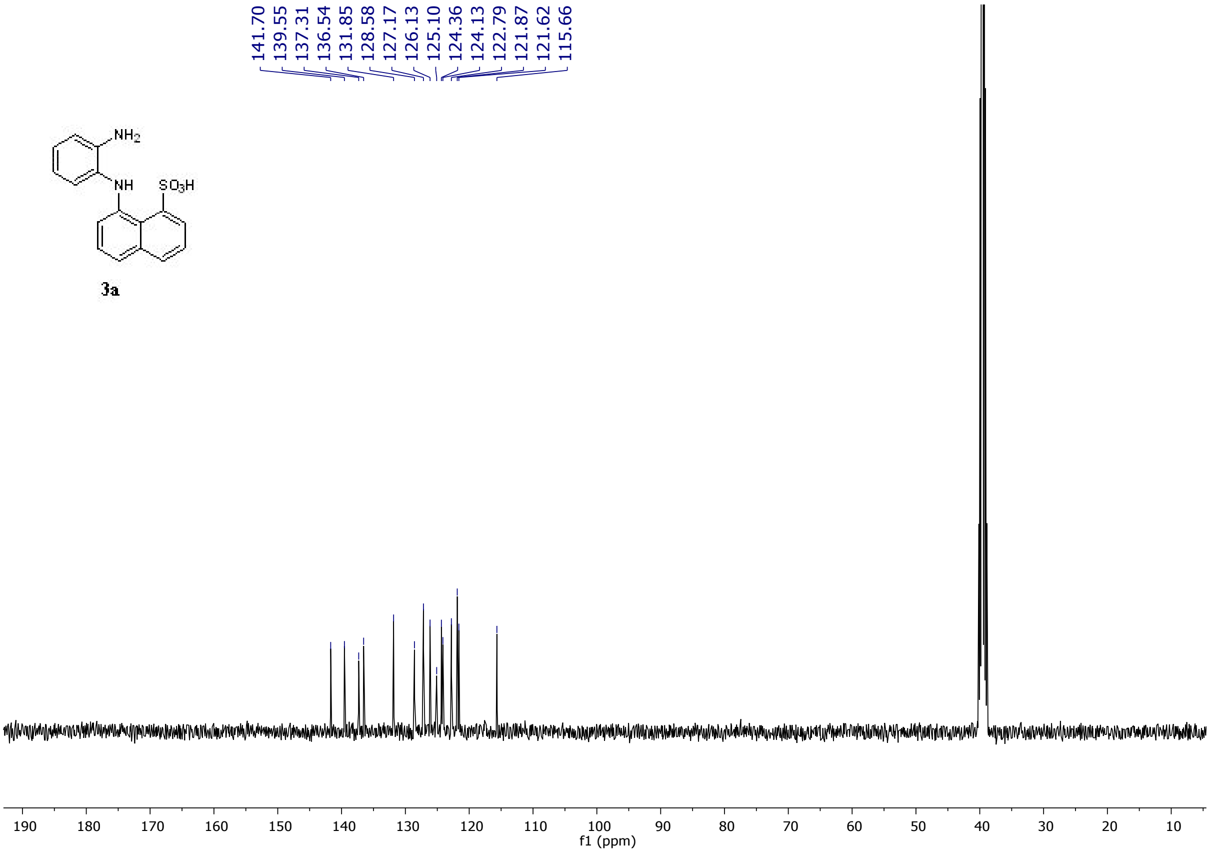

8-((2-Amino-6-chlorophenyl)amino)naphthalene-1-sulfonic acid 3b

^1H NMR (400 MHz, DMSO-d_6)

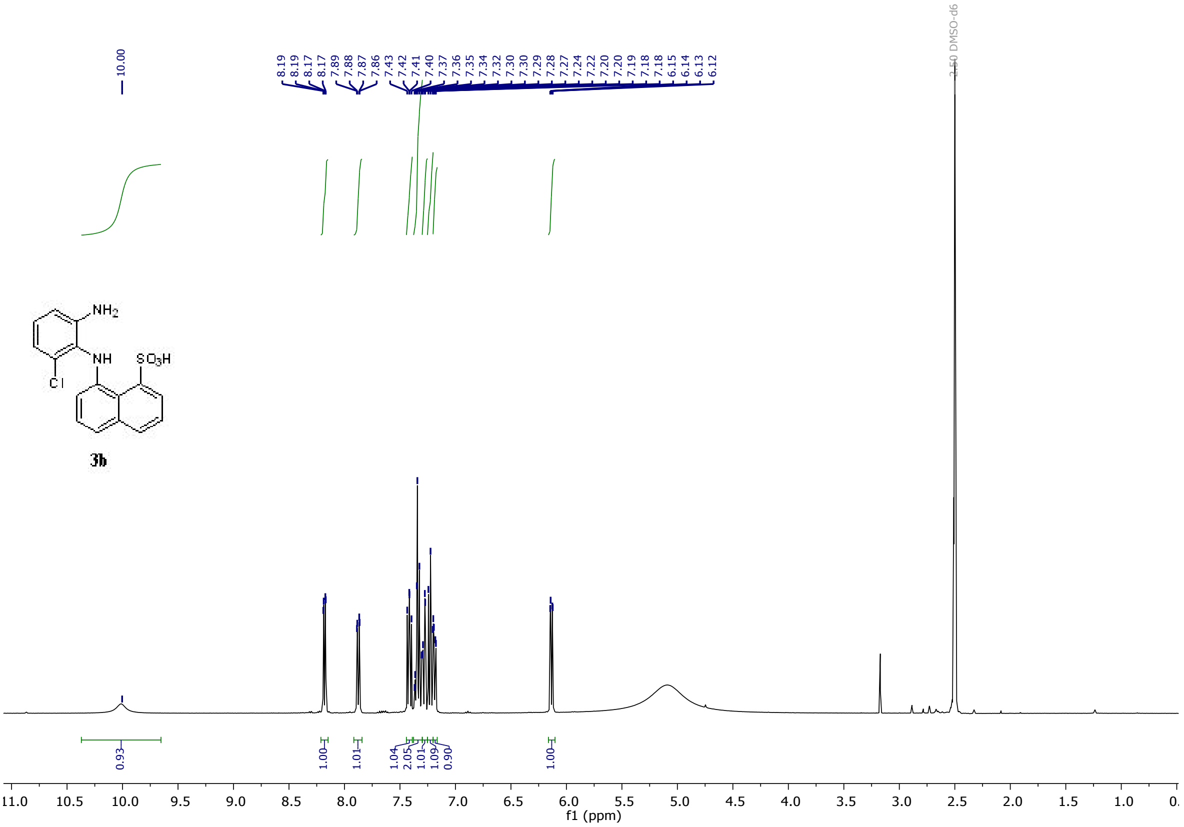

$^{13}C\{^1H\}$ NMR (101 MHz, DMSO-d_6)

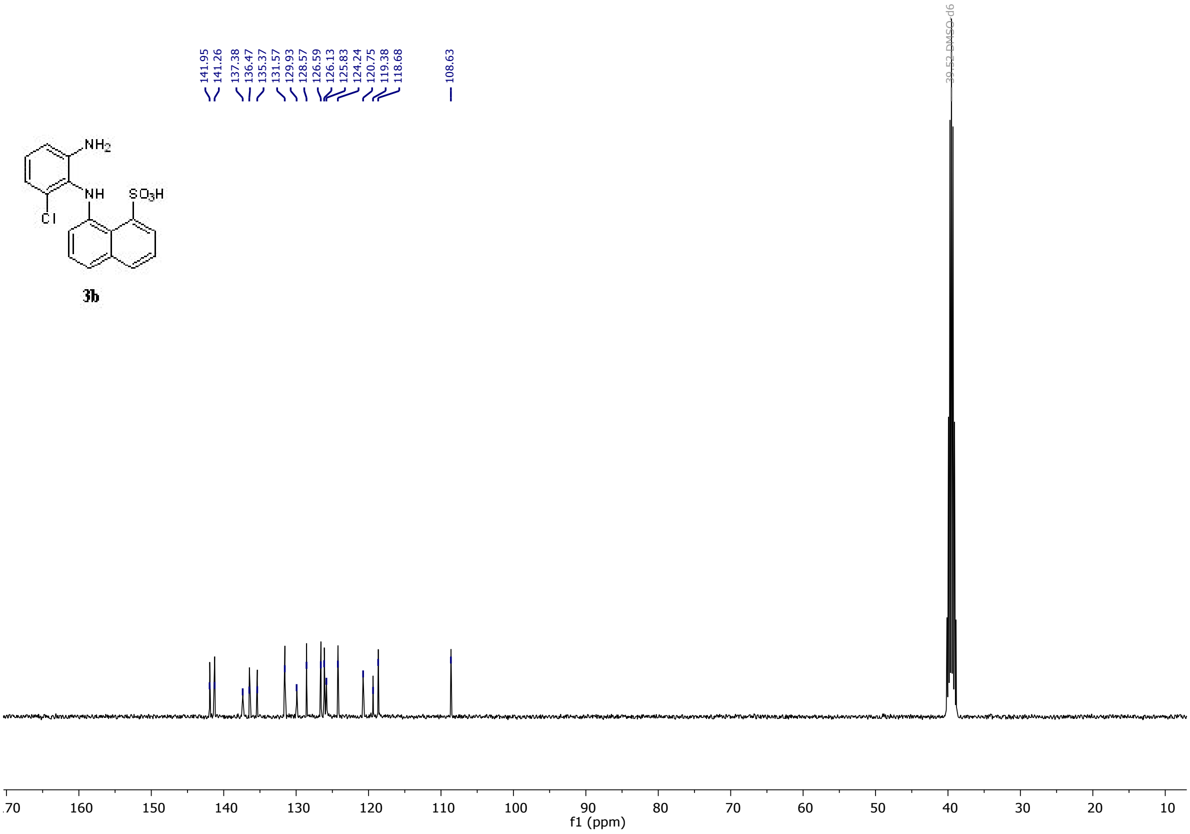

8-((2-(Isopropylamino)phenyl)amino)naphthalene-1-sulfonic acid 4a

^{1}H NMR (400 MHz, DMSO-d_6)

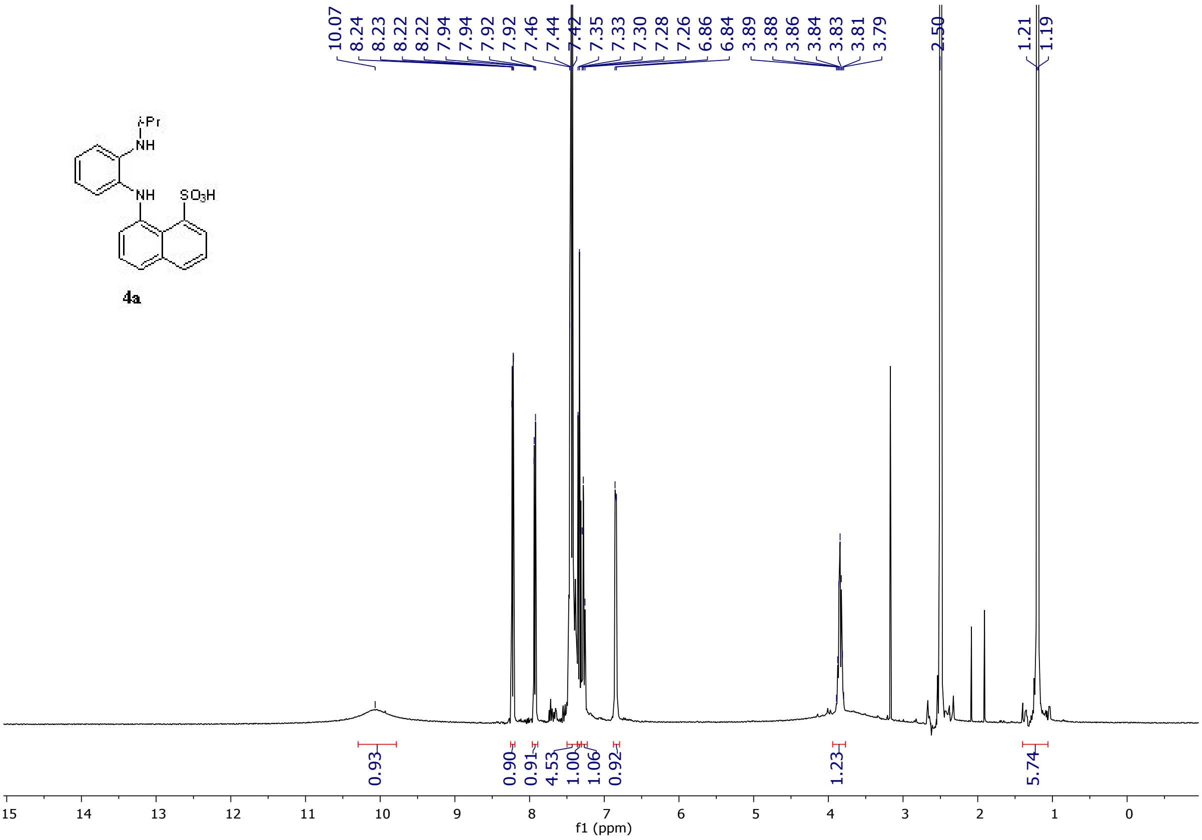

$^{13}C\{^{1}H\}$ NMR (101 MHz, DMSO-d_6)

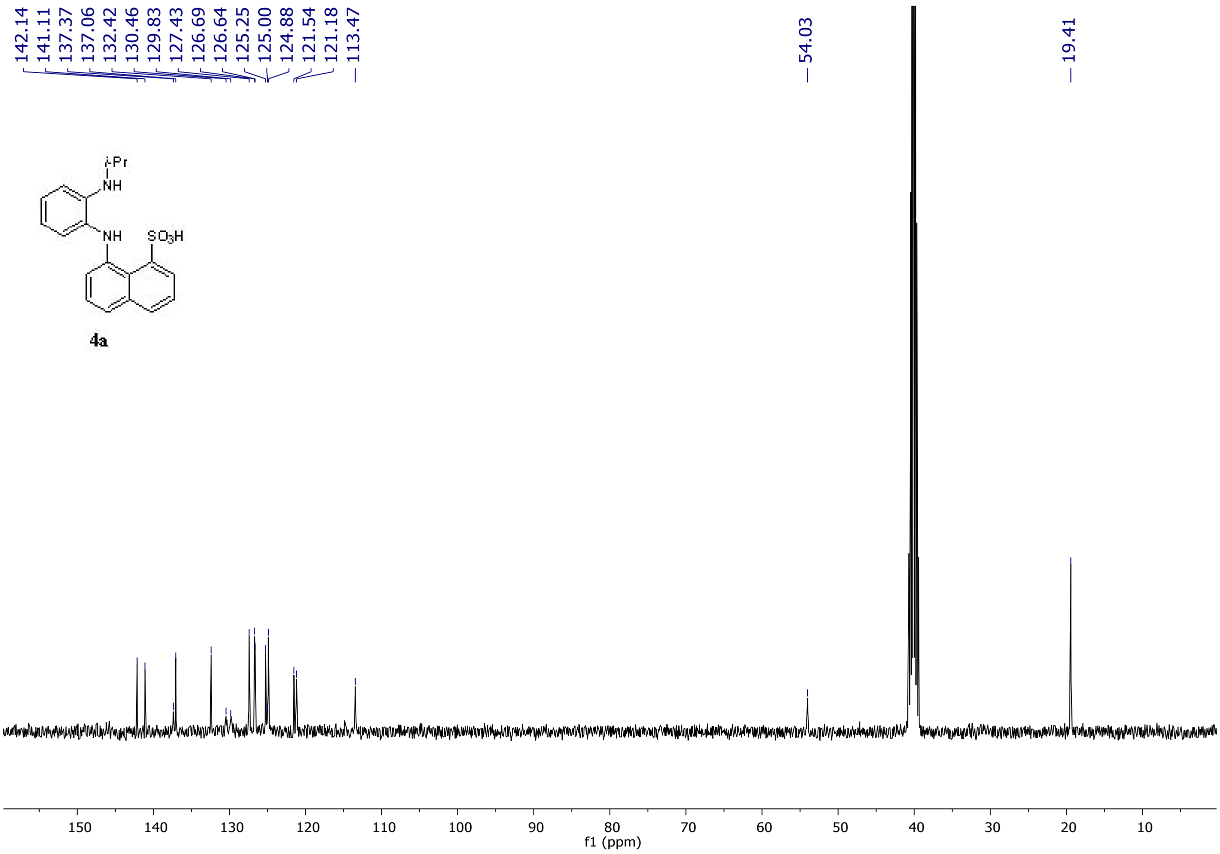

8-((2-(Cyclohexylamino)phenyl)amino)naphthalene-1-sulfonic acid 4b

^{1}H NMR (400 MHz, DMSO-d_6)

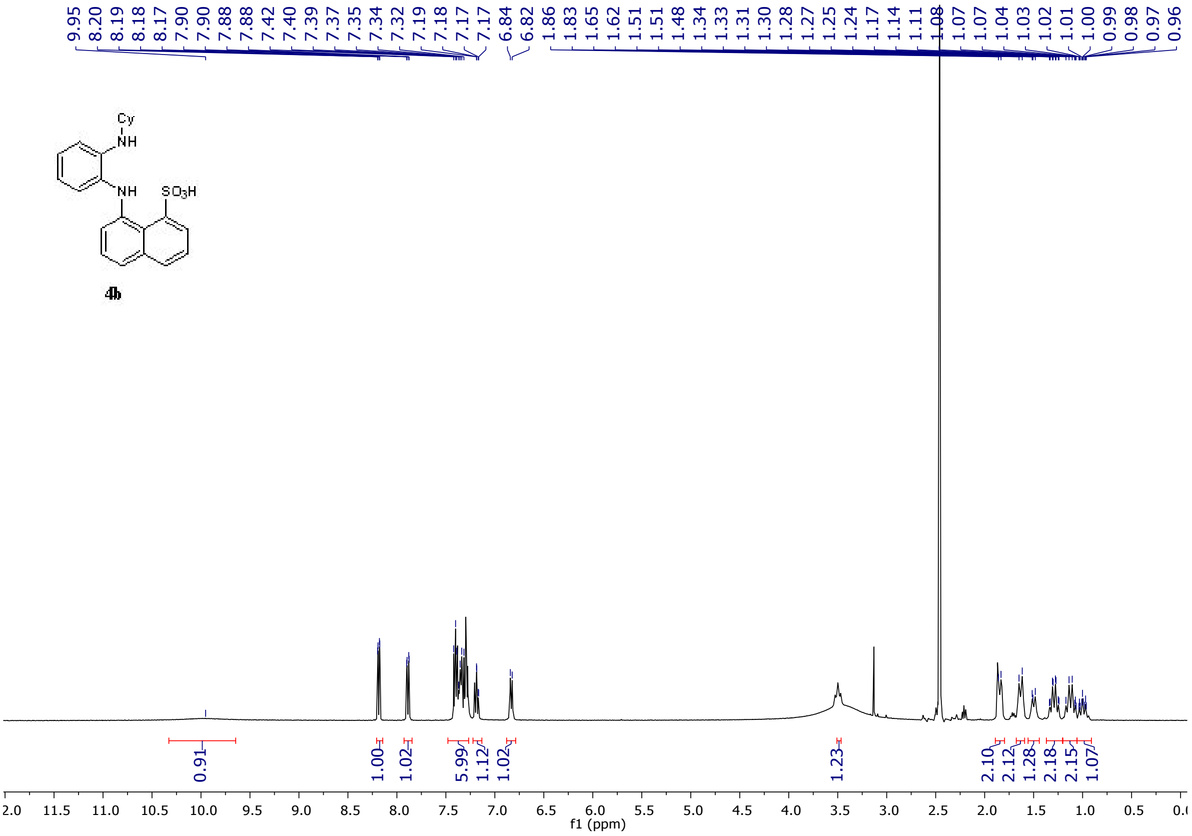

^{13}C{^{1}H} NMR (101 MHz, DMSO-d_6)

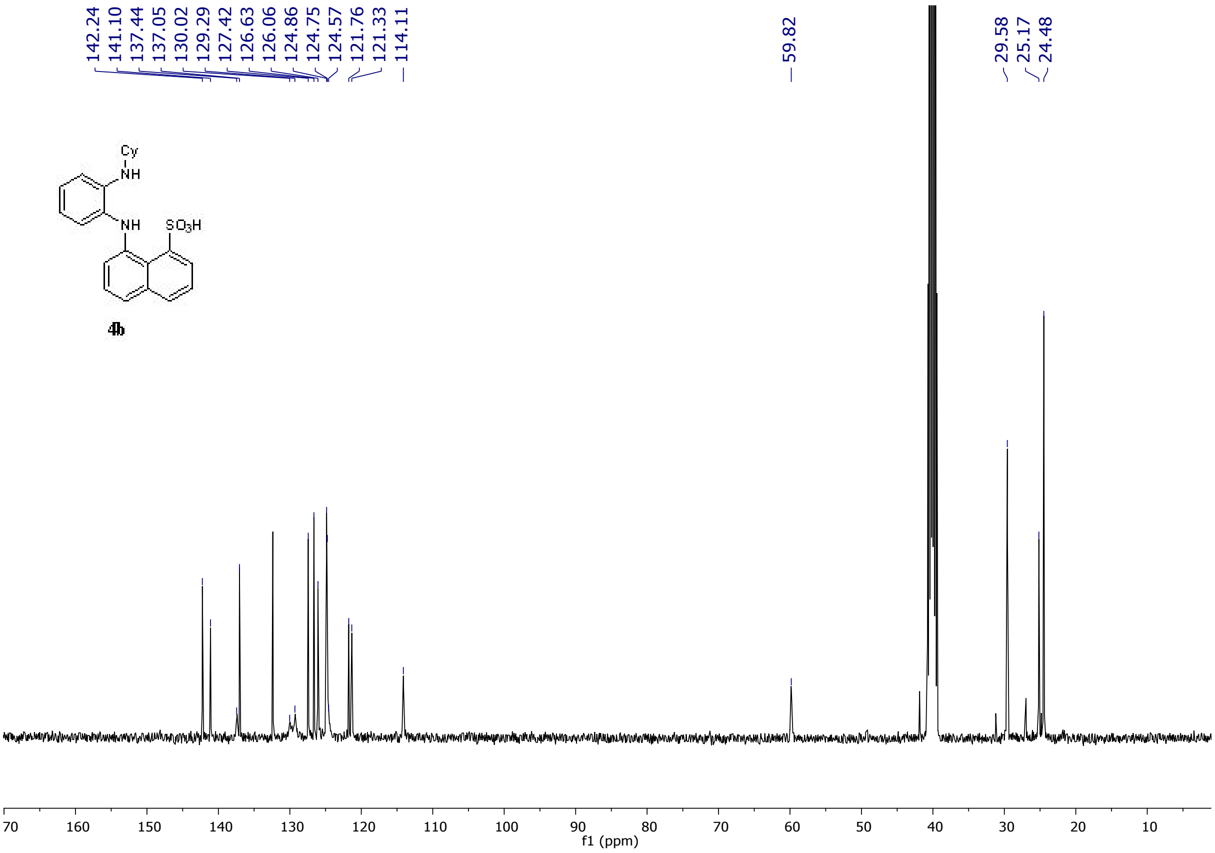

8-((2-Chloro-6-(isopropylamino)phenyl)amino)naphthalene-1-sulfonic acid 4c

^{1}H NMR (500 MHz, DMSO-d_6)

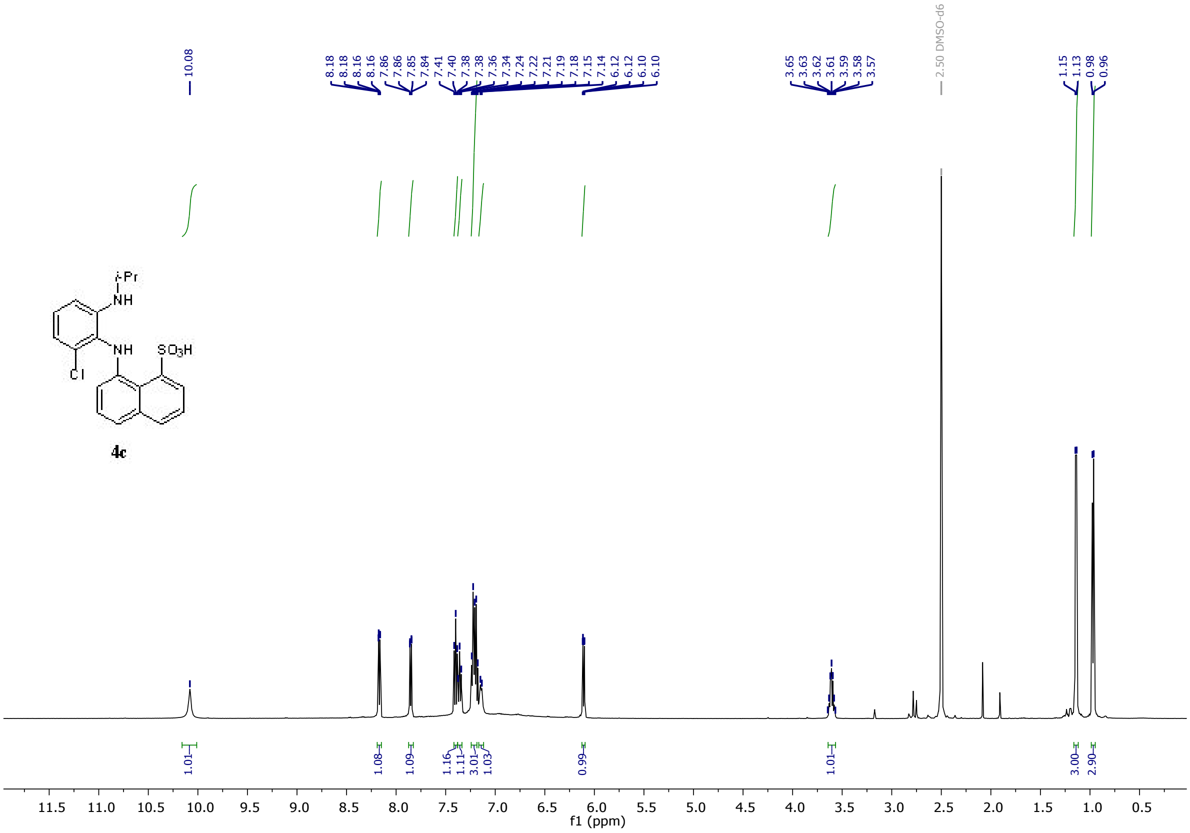

^{13}C{^{1}H} NMR (101 MHz, DMSO-d_6)

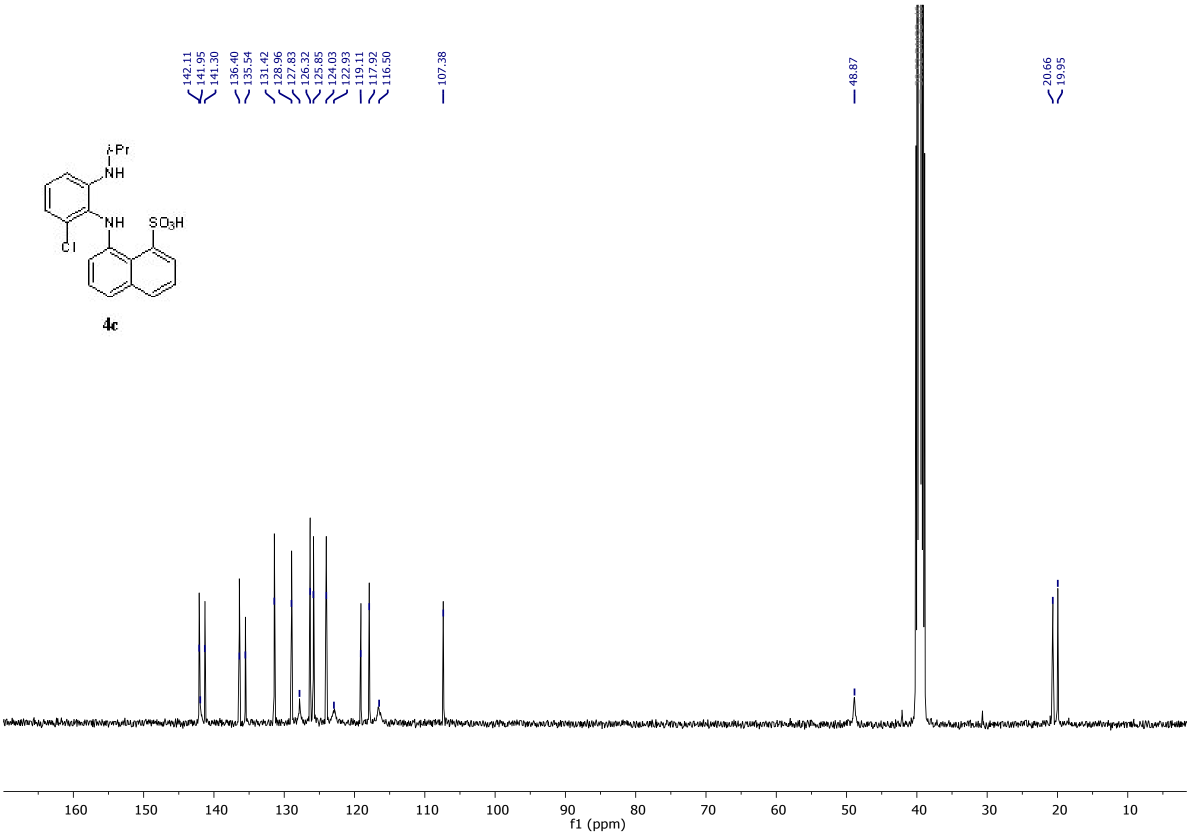

8-((2-Chloro-6-(cyclohexylamino)phenyl)amino)naphthalene-1-sulfonic acid 4d

^{1}H NMR (400 MHz, DMSO-d_6)

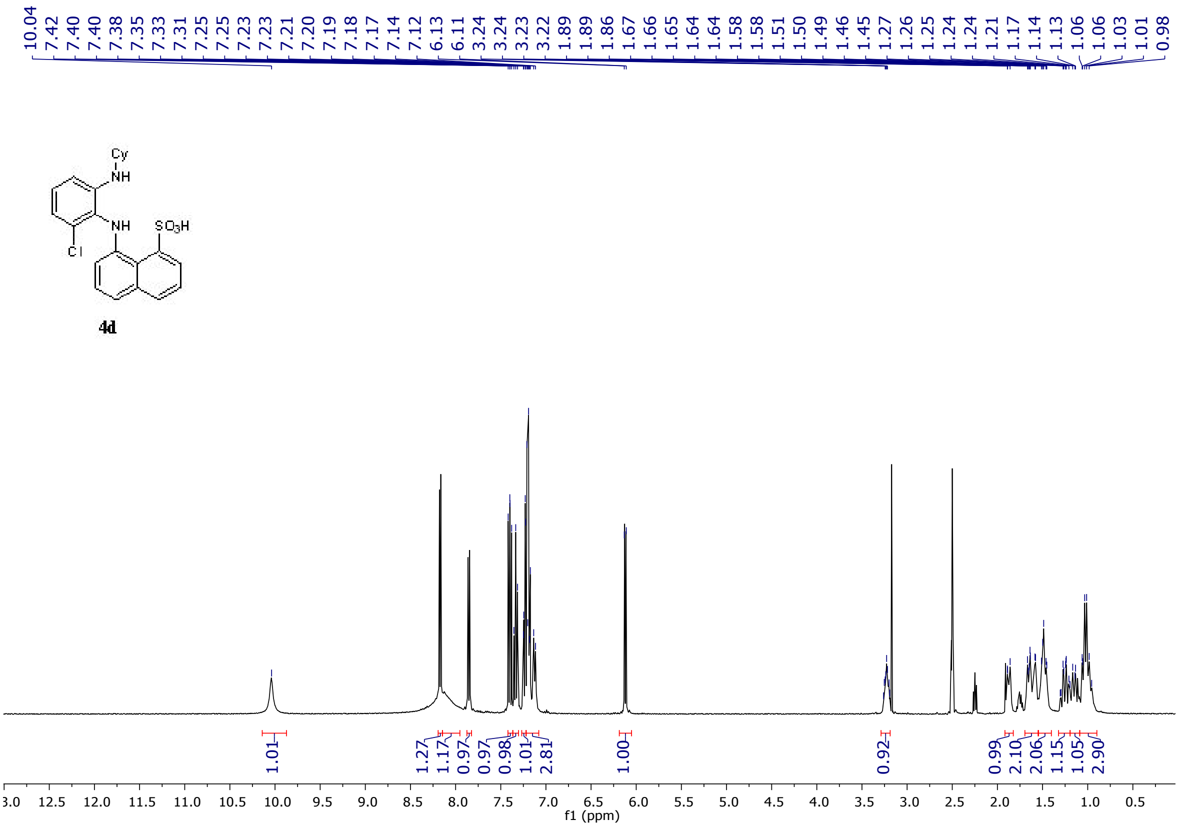

^{13}C{^{1}H} NMR (101 MHz, DMSO-d_6)

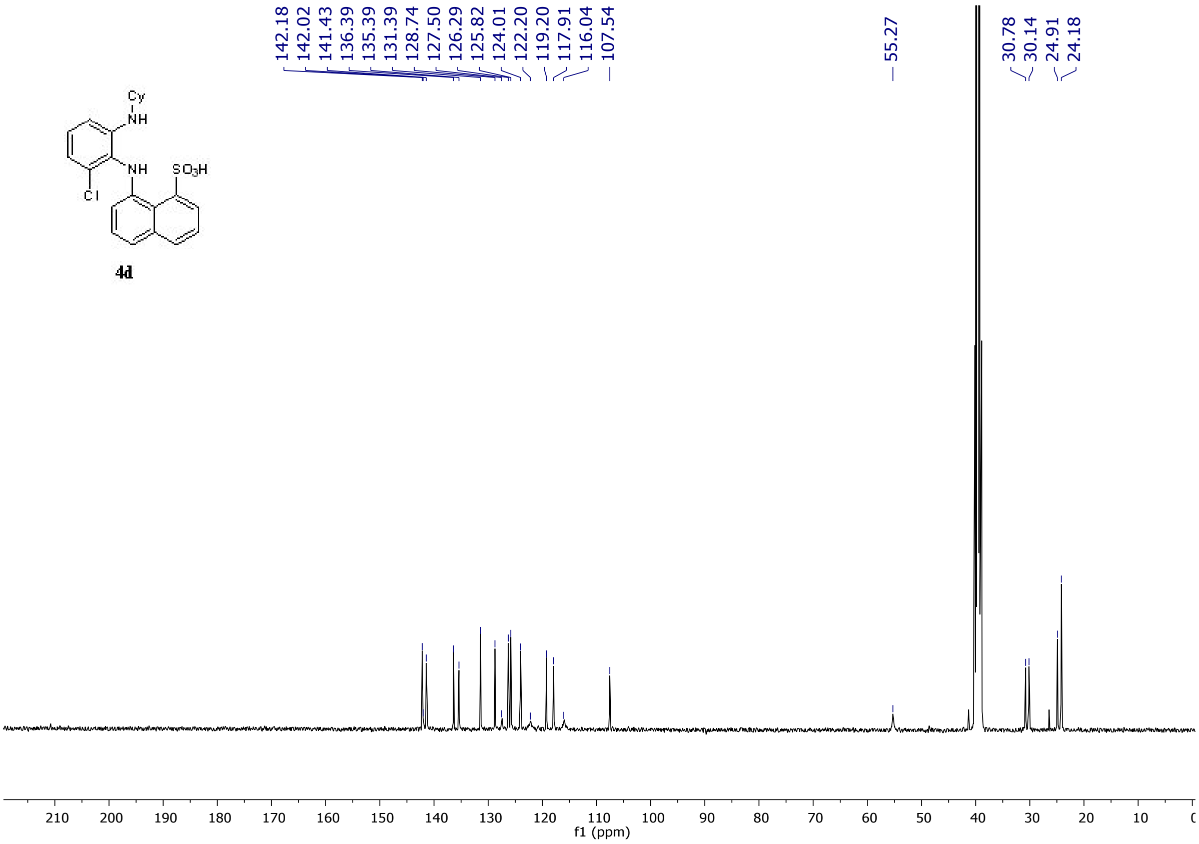

8-(2-Methyl-1*H*-benzo[*d*]imidazol-1-yl)naphthalene-1-sulfonic acid 5a

^{1}H NMR (500 MHz, DMSO-*d*$_6$)

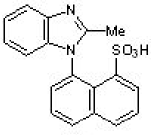

5a

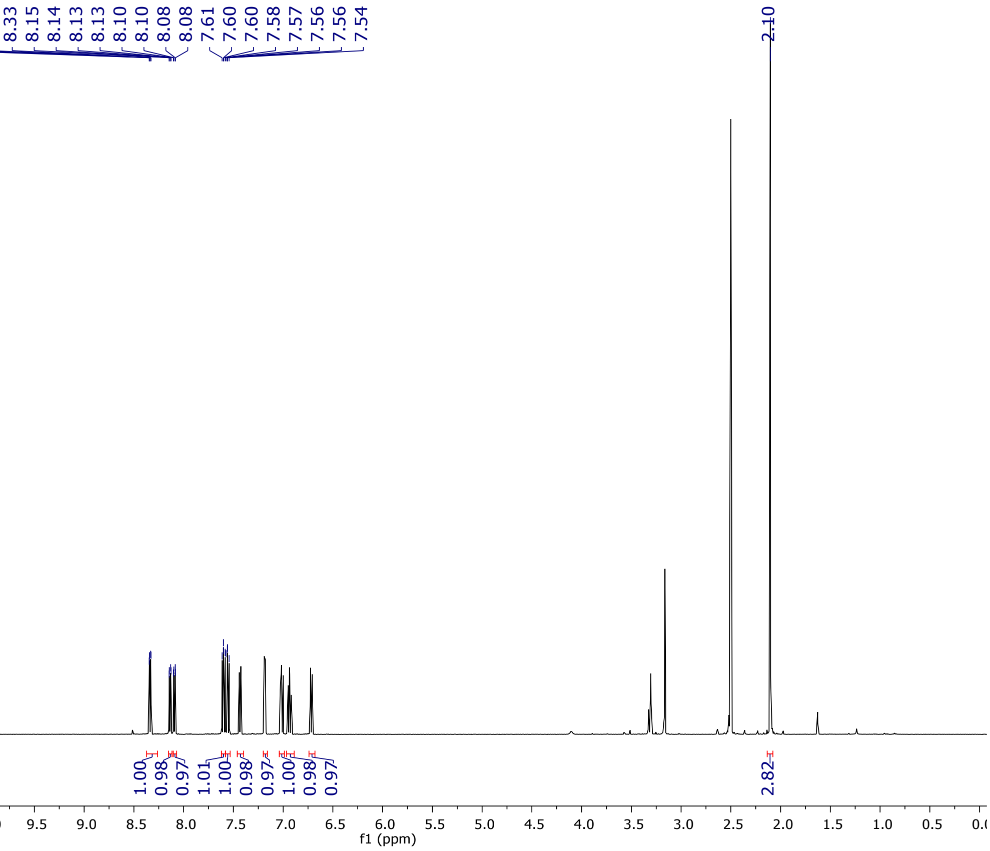

$^{13}C\{^{1}H\}$ NMR (126 MHz, DMSO-*d*$_6$)

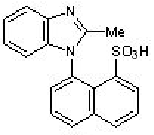

5a

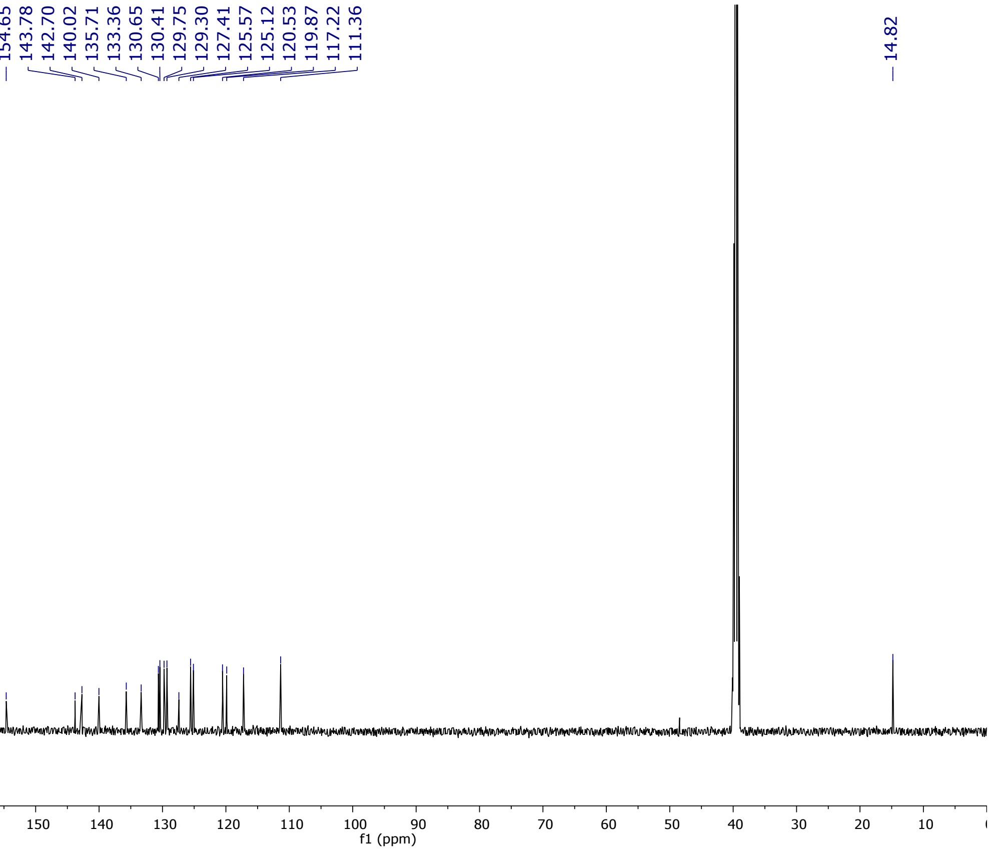

8-(2-Ethyl-1*H*-benzo[*d*]imidazol-1-yl)naphthalene-1-sulfonic acid 5b

^{1}H NMR (500 MHz, DMSO-d_6)

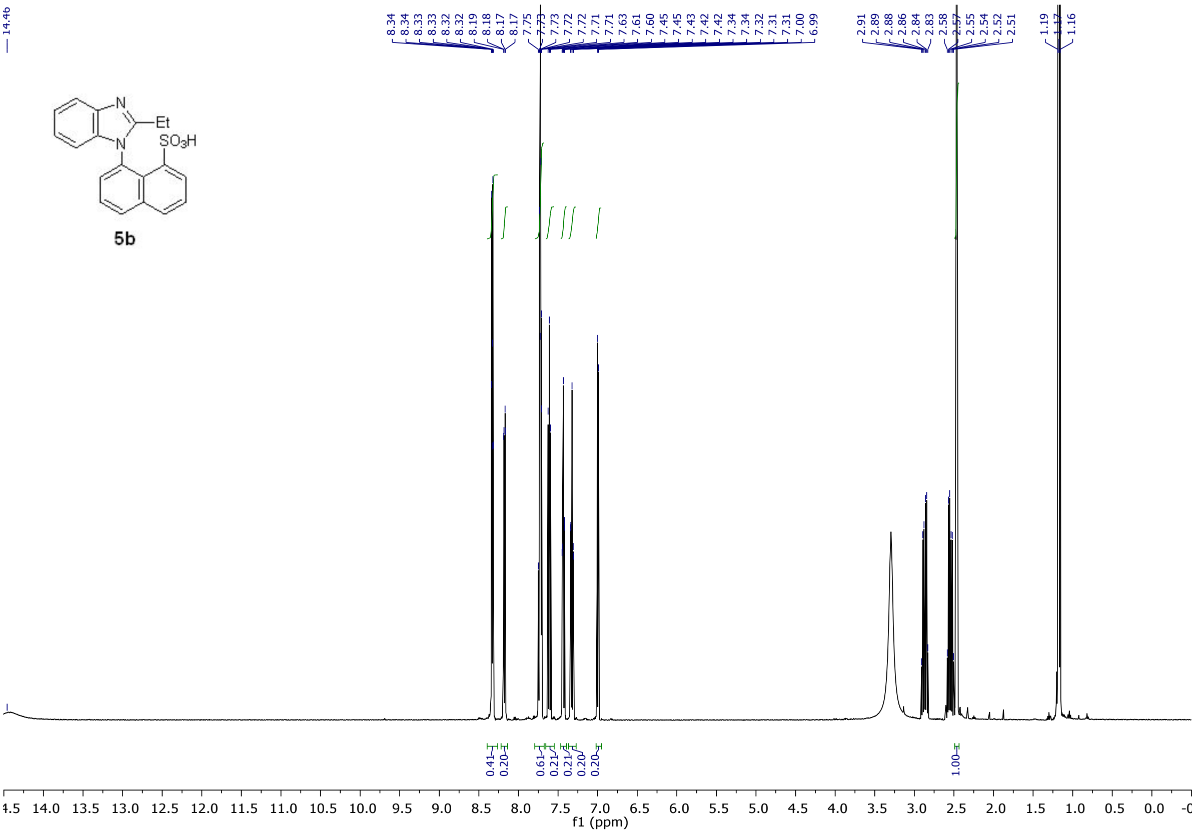

^{13}C{^{1}H} NMR (126 MHz, DMSO-d_6)

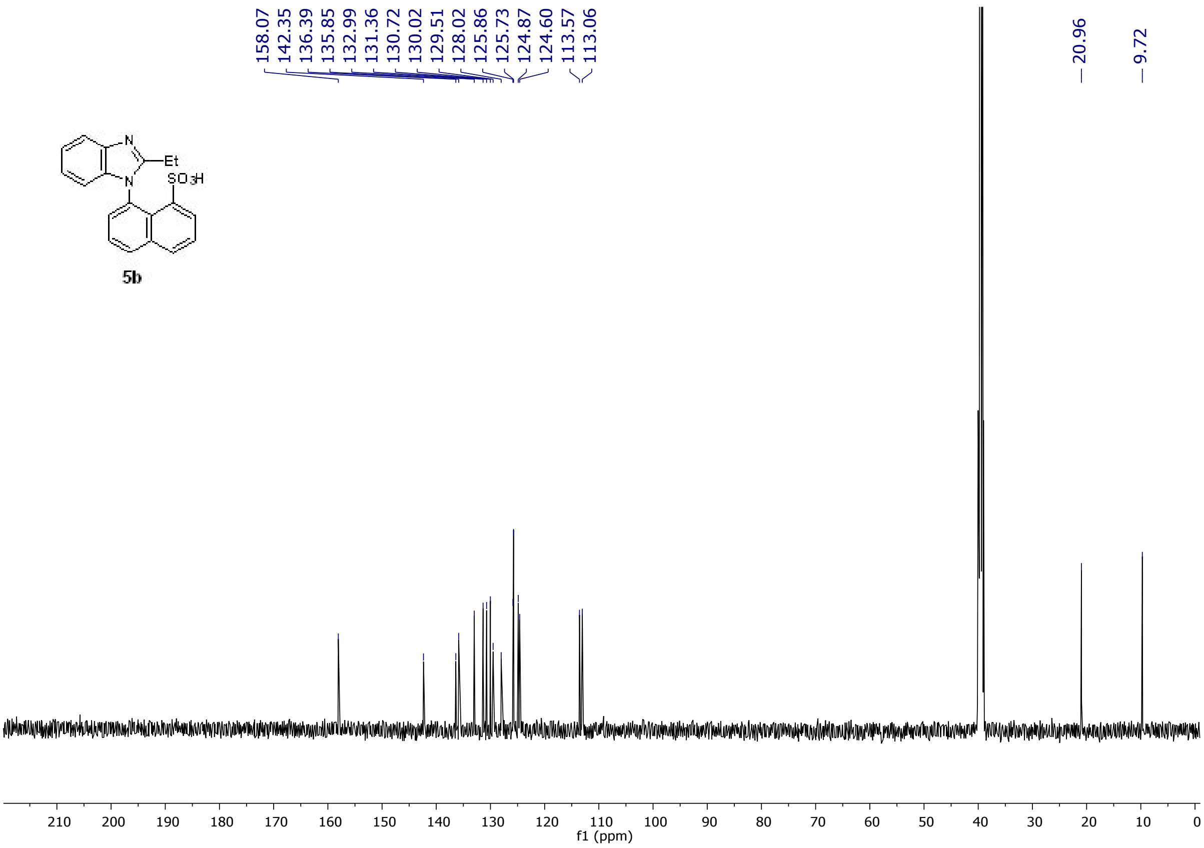

8-(2-Isopropyl-1*H*-benzo[*d*]imidazol-1-yl)naphthalene-1-sulfonic acid 5c

^{1}H NMR (500 MHz, DMSO-d_6)

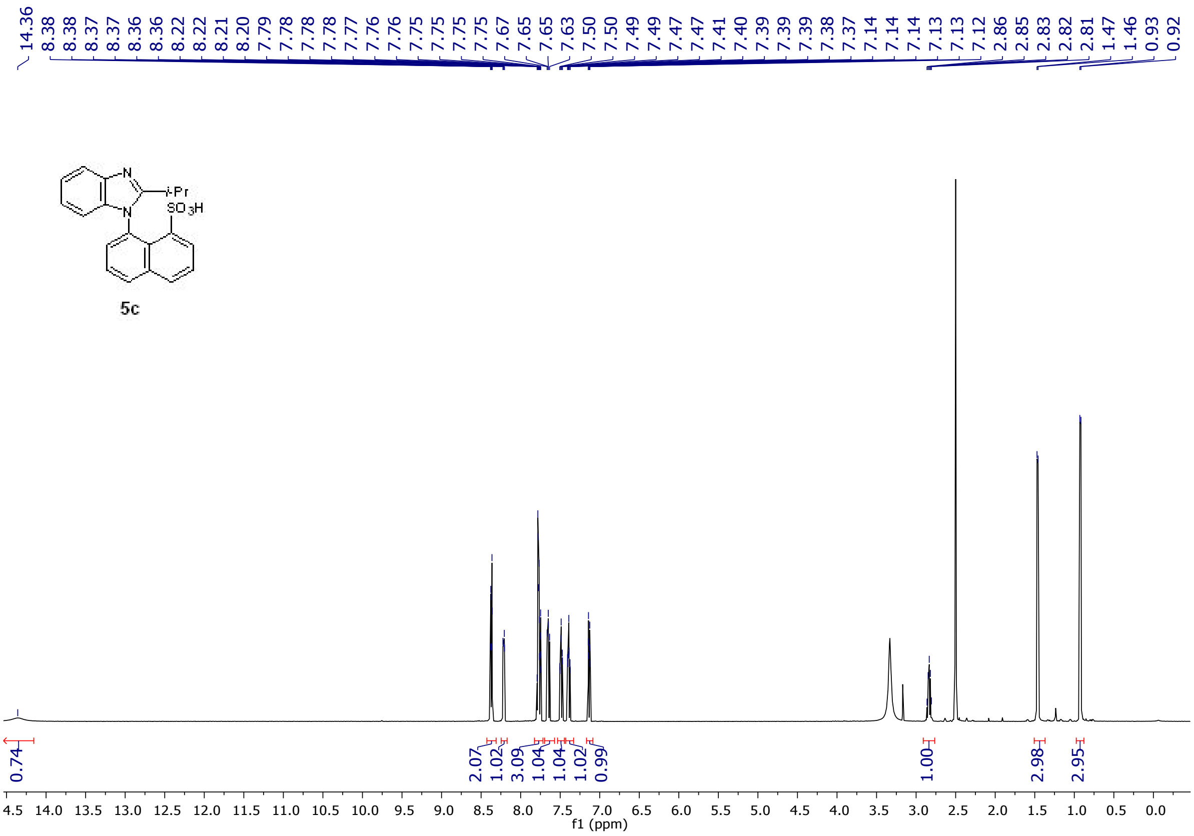

^{13}C{^{1}H} NMR (126 MHz, DMSO-d_6)

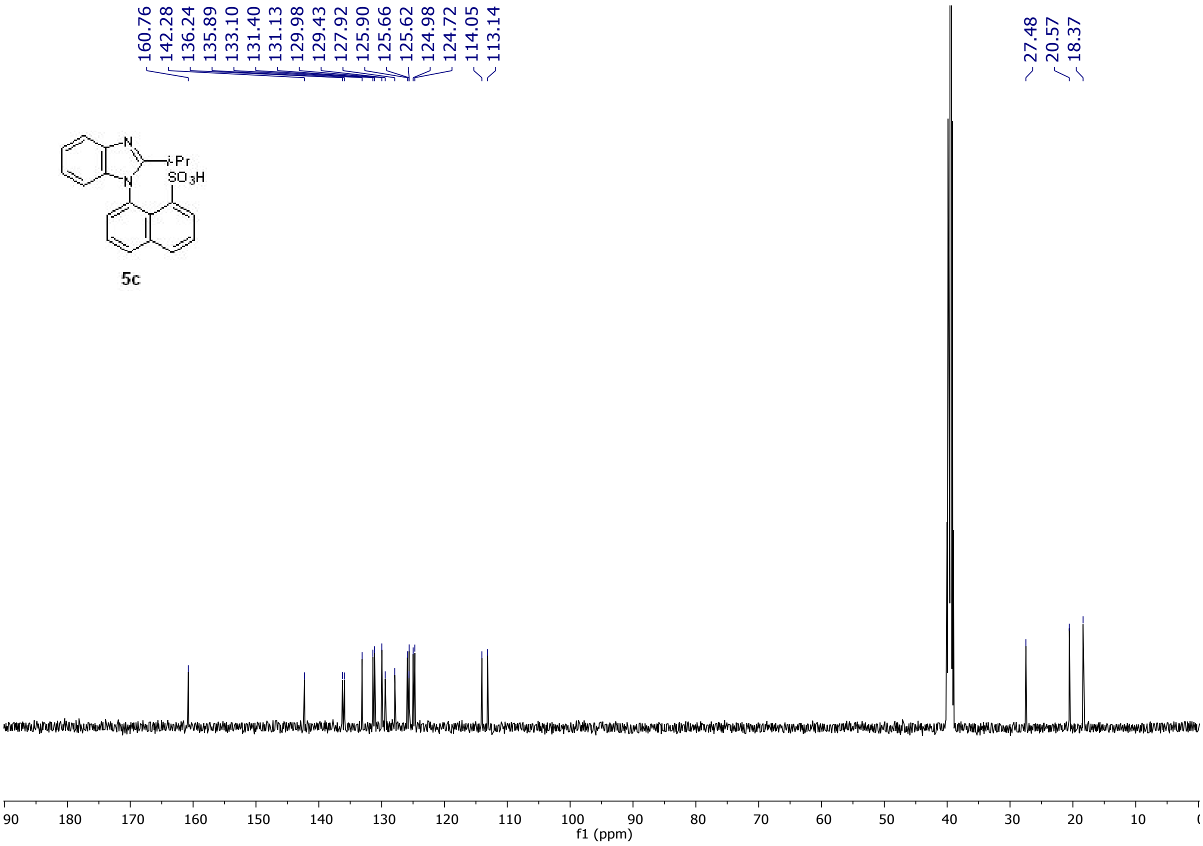

8-(7-Chloro-2-methyl-1*H*-benzo[*d*]imidazol-1-yl)naphthalene-1-sulfonic acid 5d

^{1}H NMR (400 MHz, DMSO-d_6)

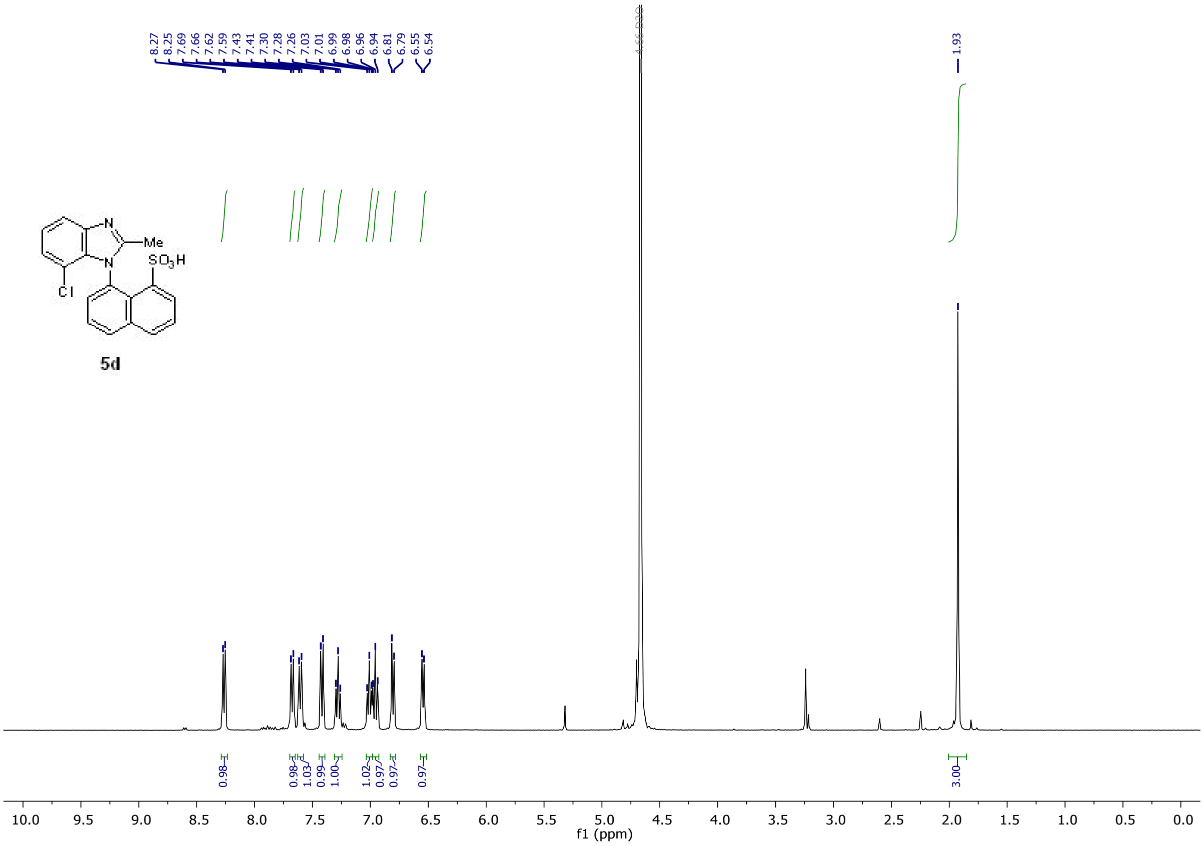

$^{13}C\{^{1}H\}$ NMR (101 MHz, DMSO-d_6)

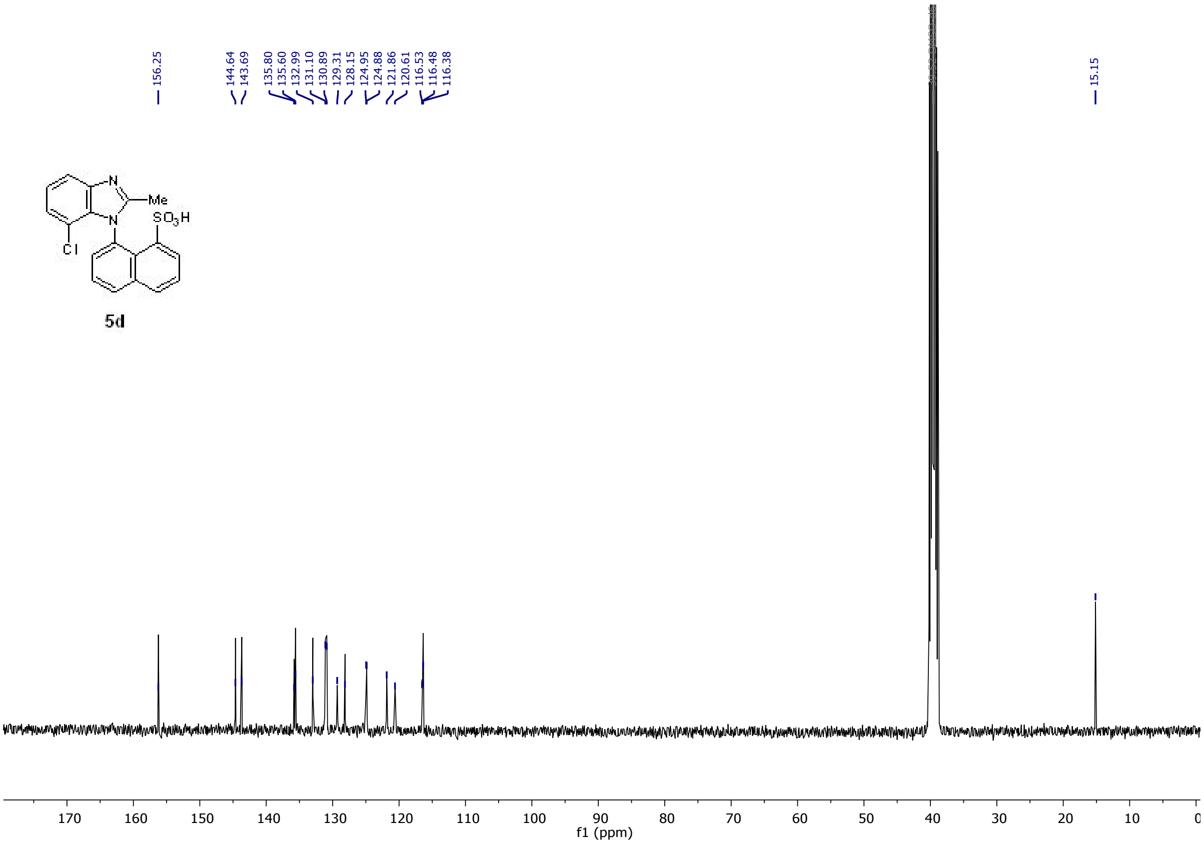

8-(7-Chloro-2-ethyl-1*H*-benzo[*d*]imidazol-1-yl)naphthalene-1-sulfonic acid 5e

^{1}H NMR (400 MHz, DMSO-d_6)

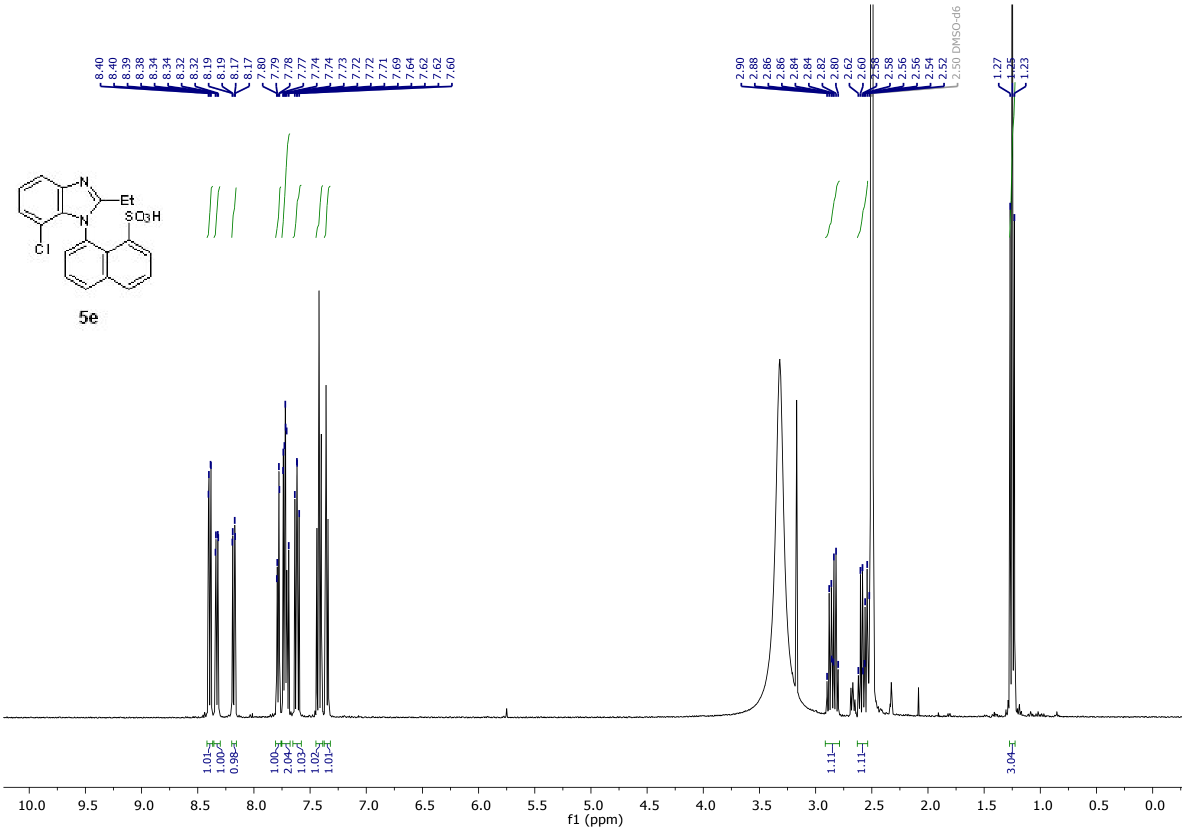

$^{13}C\{^{1}H\}$ NMR (101 MHz, DMSO-d_6)

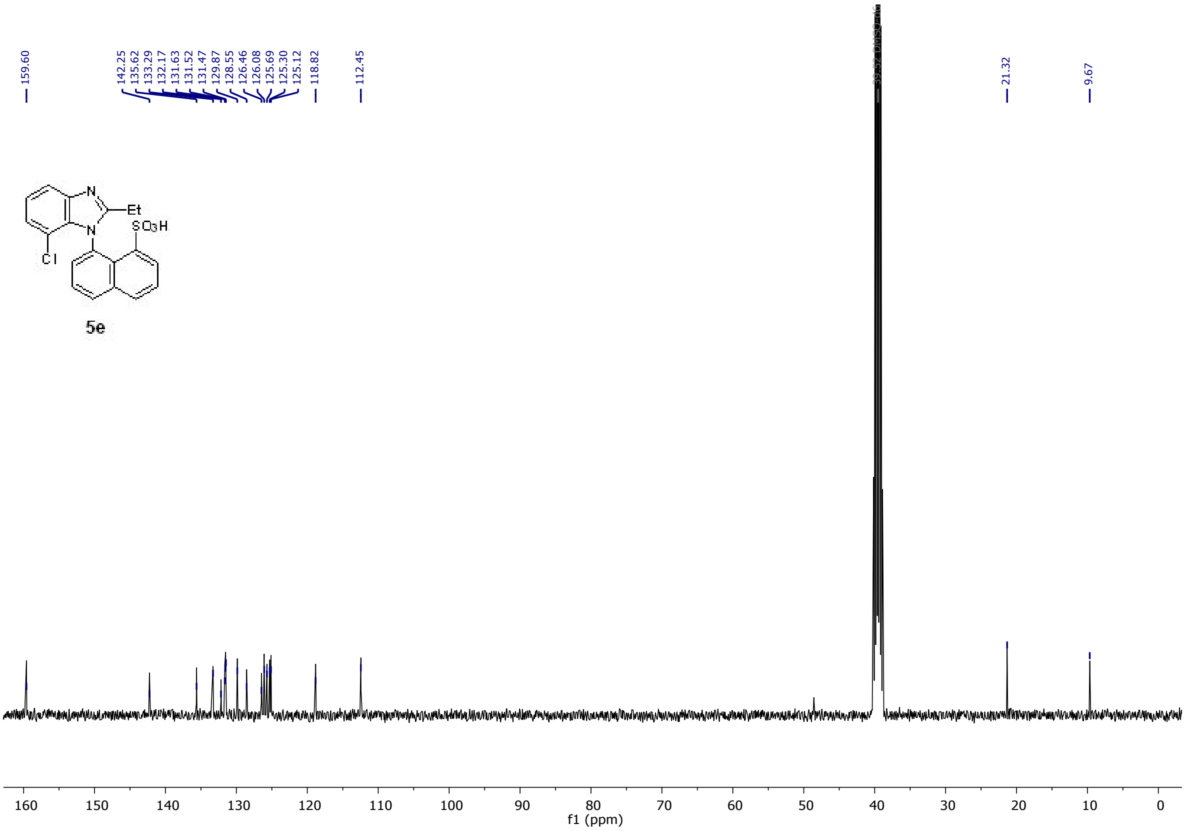

8-(7-Chloro-2-isopropyl-1*H*-benzo[*d*]imidazol-1-yl)naphthalene-1-sulfonic acid 5f

^{1}H NMR (400 MHz, DMSO-d_6)

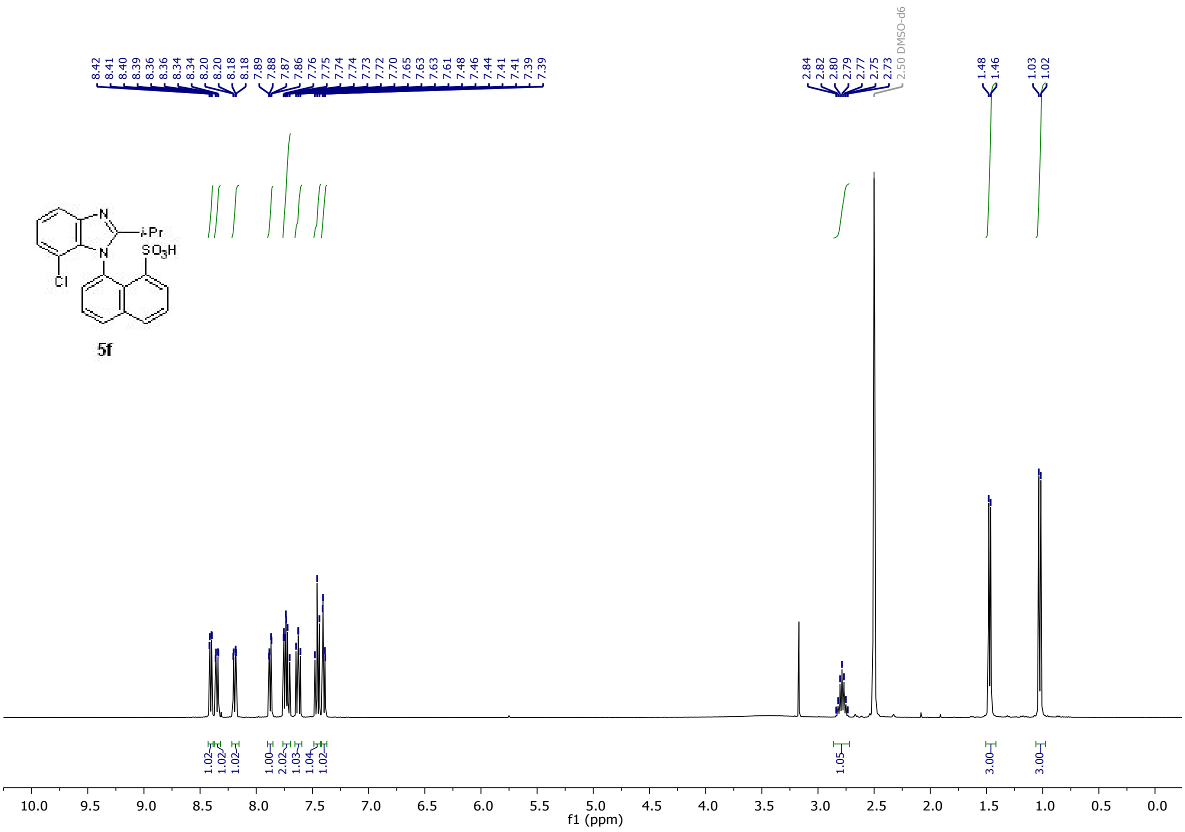

^{13}C{^{1}H} NMR (101 MHz, DMSO-d_6)

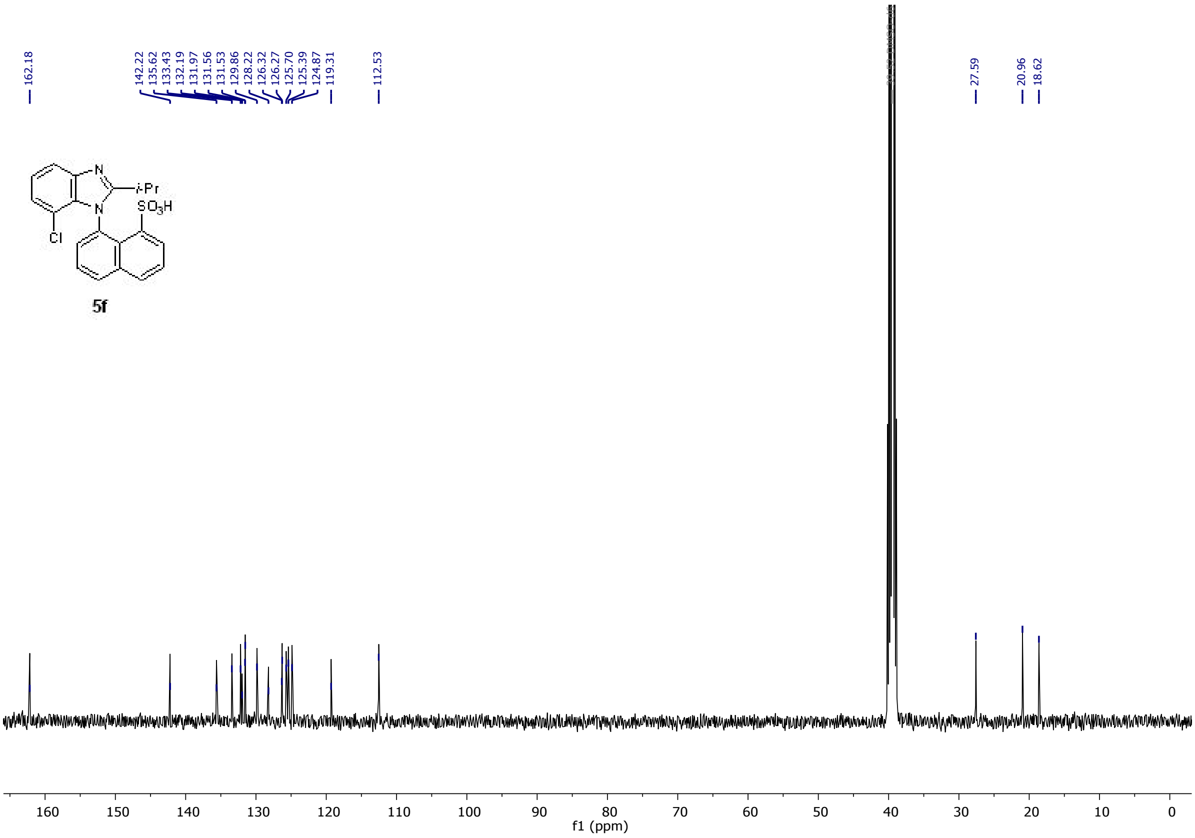

8-(7-Chloro-2-phenyl-1*H*-benzo[*d*]imidazol-1-yl)naphthalene-1-sulfonic acid 5g

^{1}H NMR (400 MHz, DMSO-d_6)

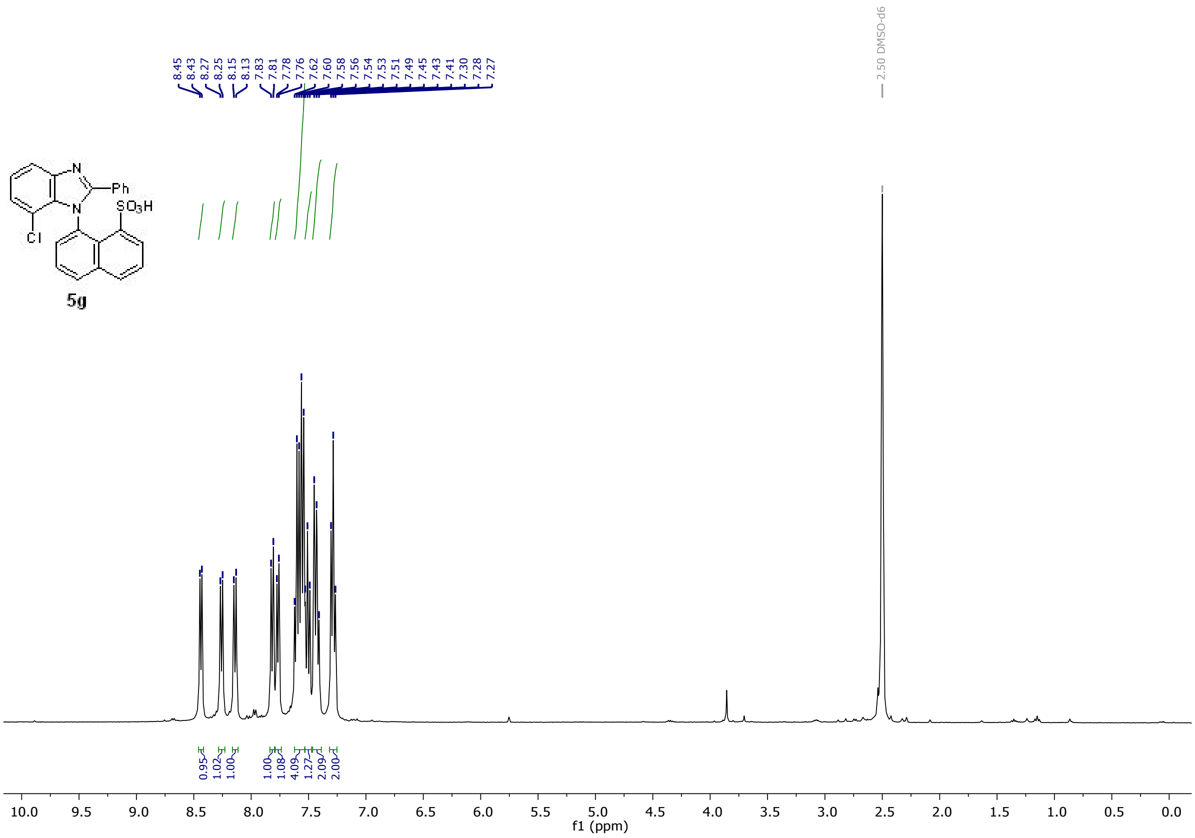

^{13}C{^{1}H} NMR (101 MHz, DMSO-d_6)

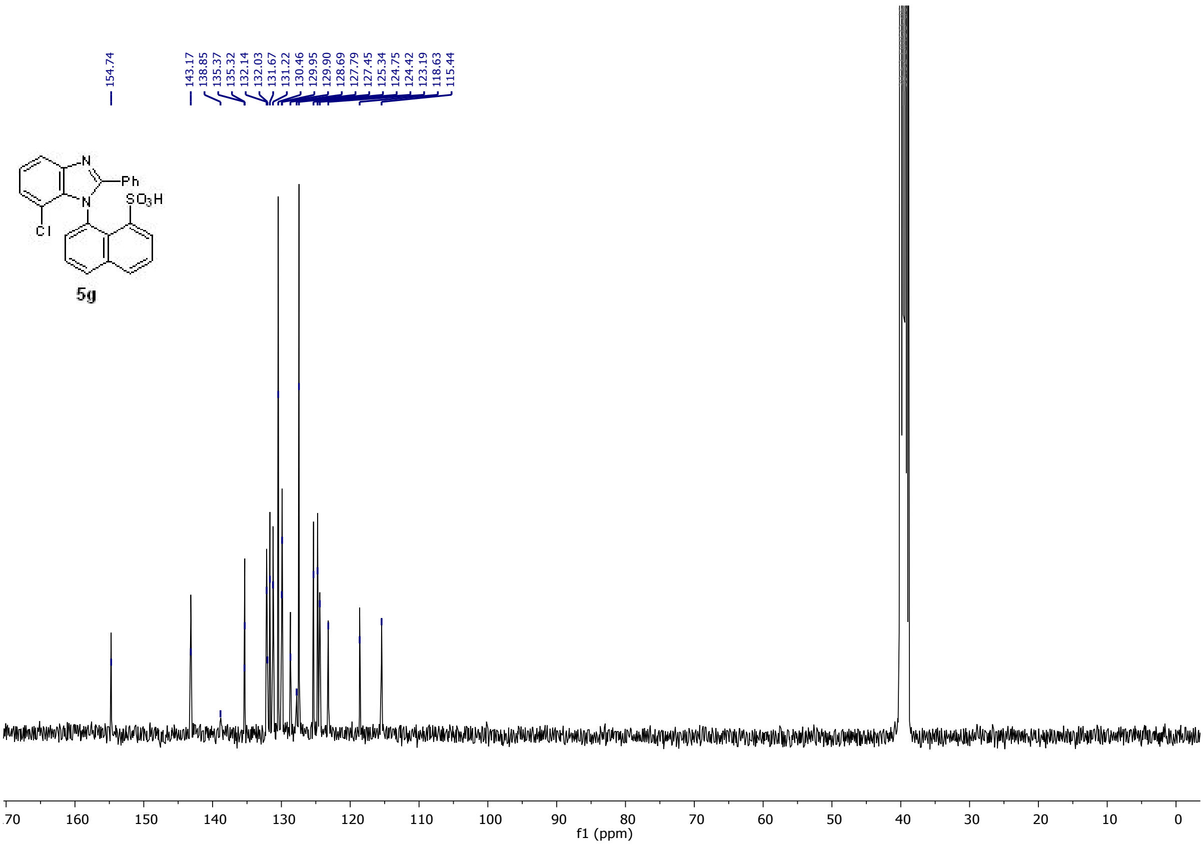

8-(3-Isopropyl-2-oxo-2,3-dihydro-1*H*-benzo[*d*]imidazol-1-yl)naphthalene-1-sulfonic acid 6a

^{1}H NMR (400 MHz, DMSO-d_6)

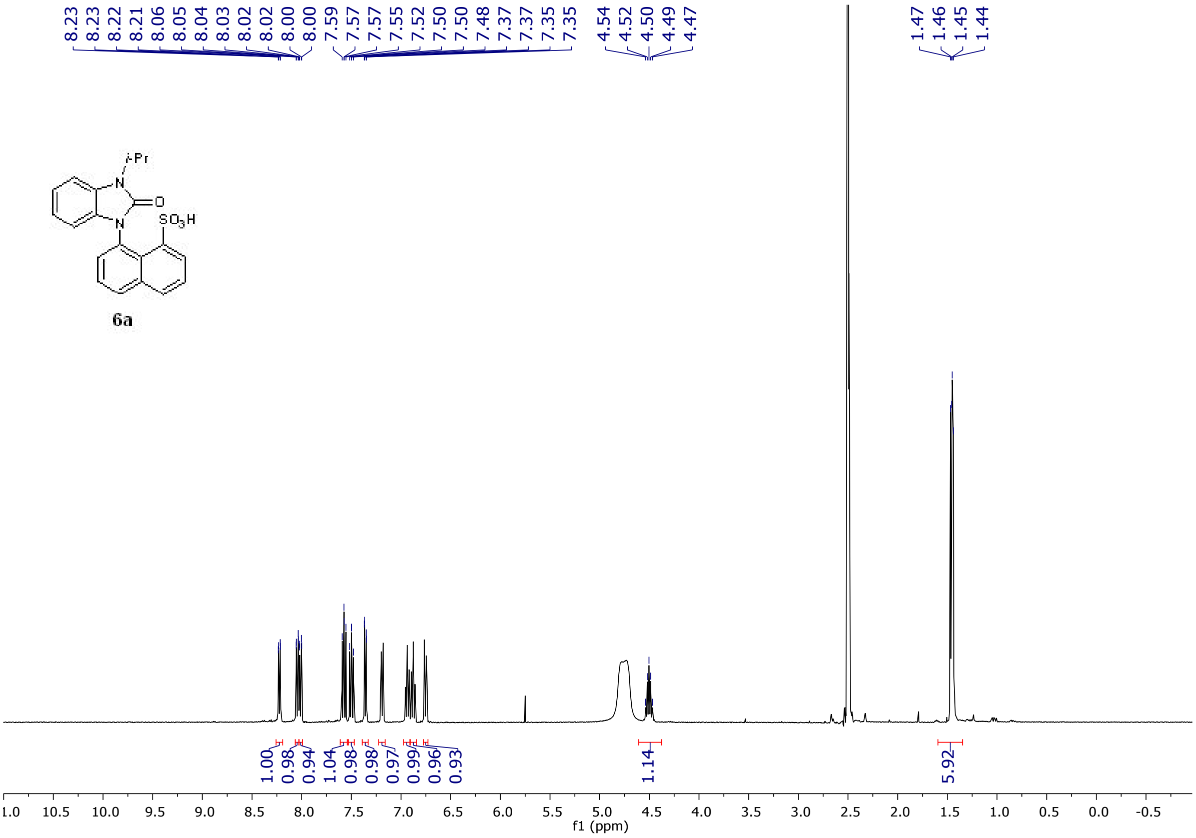

^{13}C{^{1}H} NMR (101 MHz, DMSO-d_6)

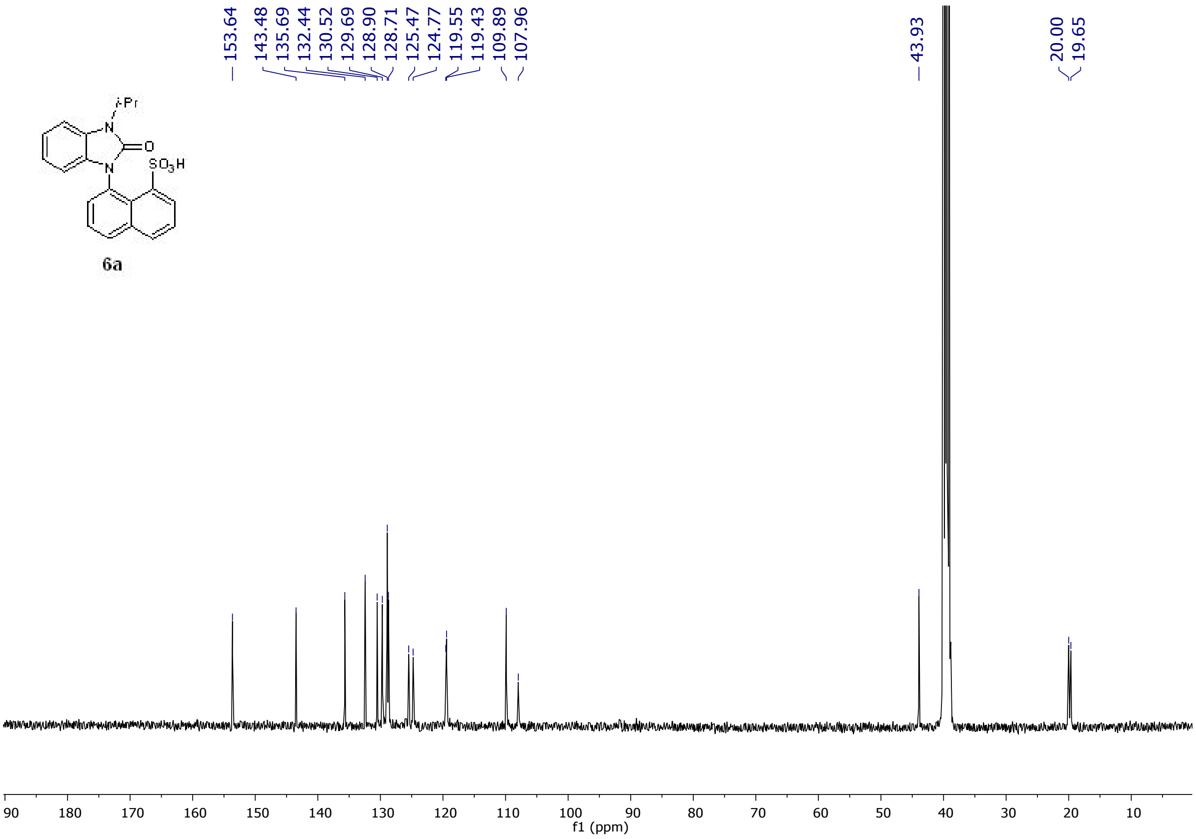

8-(3-Cyclohexyl-2-oxo-2,3-dihydro-1*H*-benzo[*d*]imidazol-1-yl)naphthalene-1-sulfonic acid 6b

^{1}H NMR (500 MHz, DMSO-d_6)

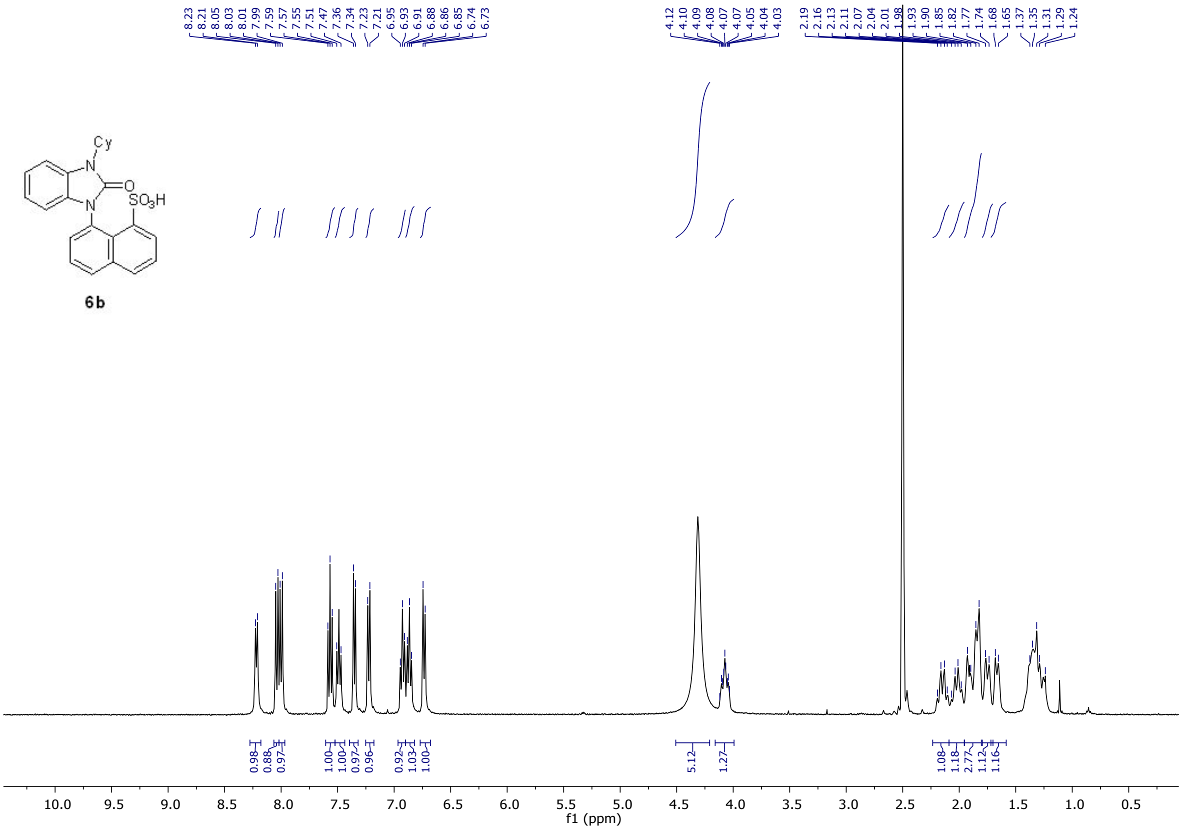

6b

^{13}C{^{1}H} NMR (101 MHz, DMSO-d_6)

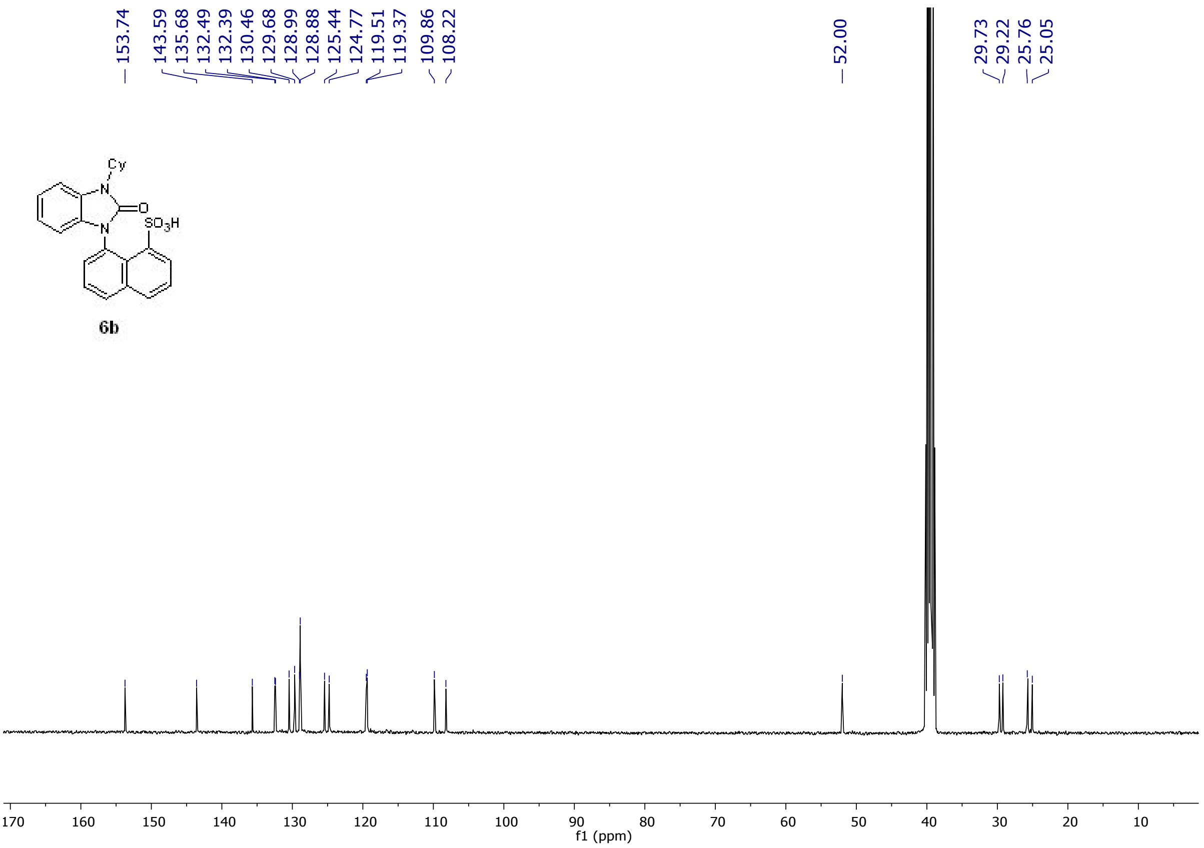

6b

8-(7-Chloro-3-isopropyl-2-oxo-2,3-dihydro-1*H*-benzo[*d*]imidazol-1-yl)naphthalene-1-sulfonic acid 6c

^{1}H NMR (400 MHz, $CDCl_3$)

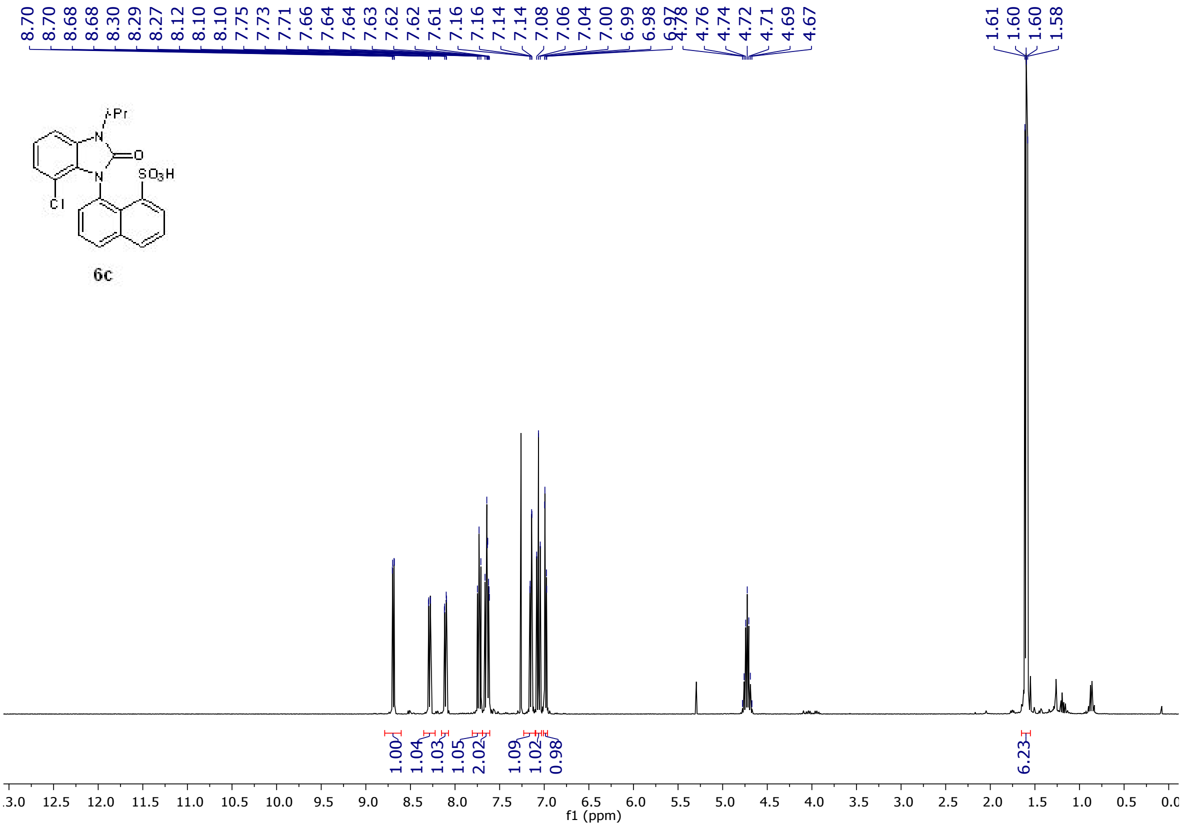

^{13}C{^{1}H} NMR (101 MHz, $CDCl_3$)

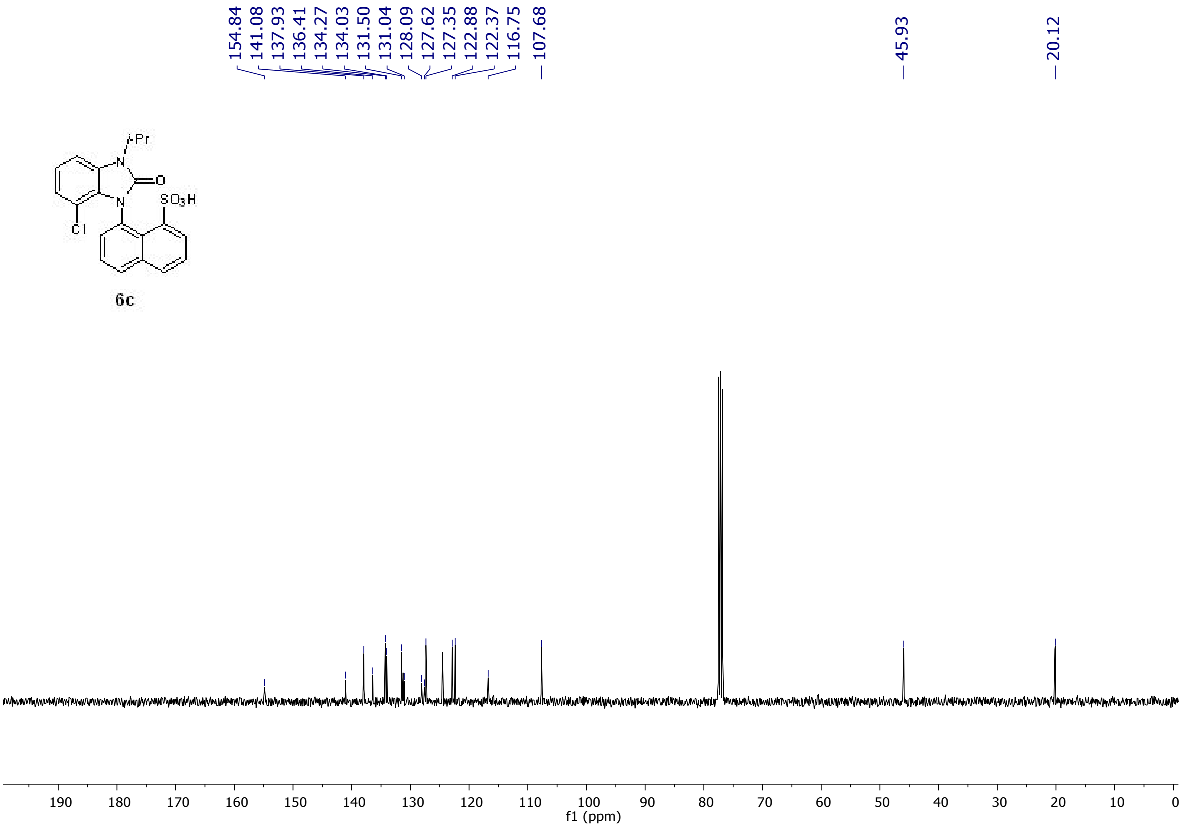

8-(7-Chloro-3-cyclohexyl-2-oxo-2,3-dihydro-1*H*-benzo[*d*]imidazol-1-yl)naphthalene-1-sulfonic acid 6d

^{1}H NMR (400 MHz, DMSO-d_6)

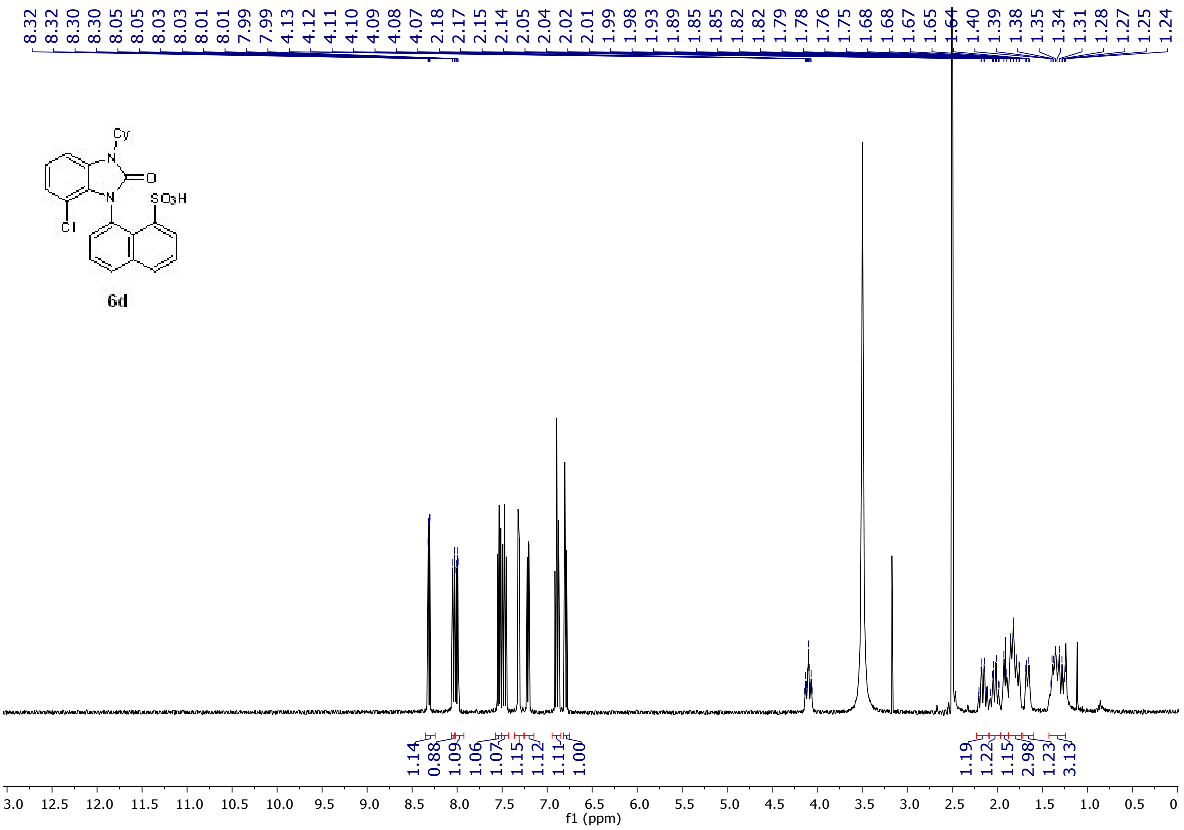

$^{13}C\{^{1}H\}$ NMR (101 MHz, DMSO-d_6)

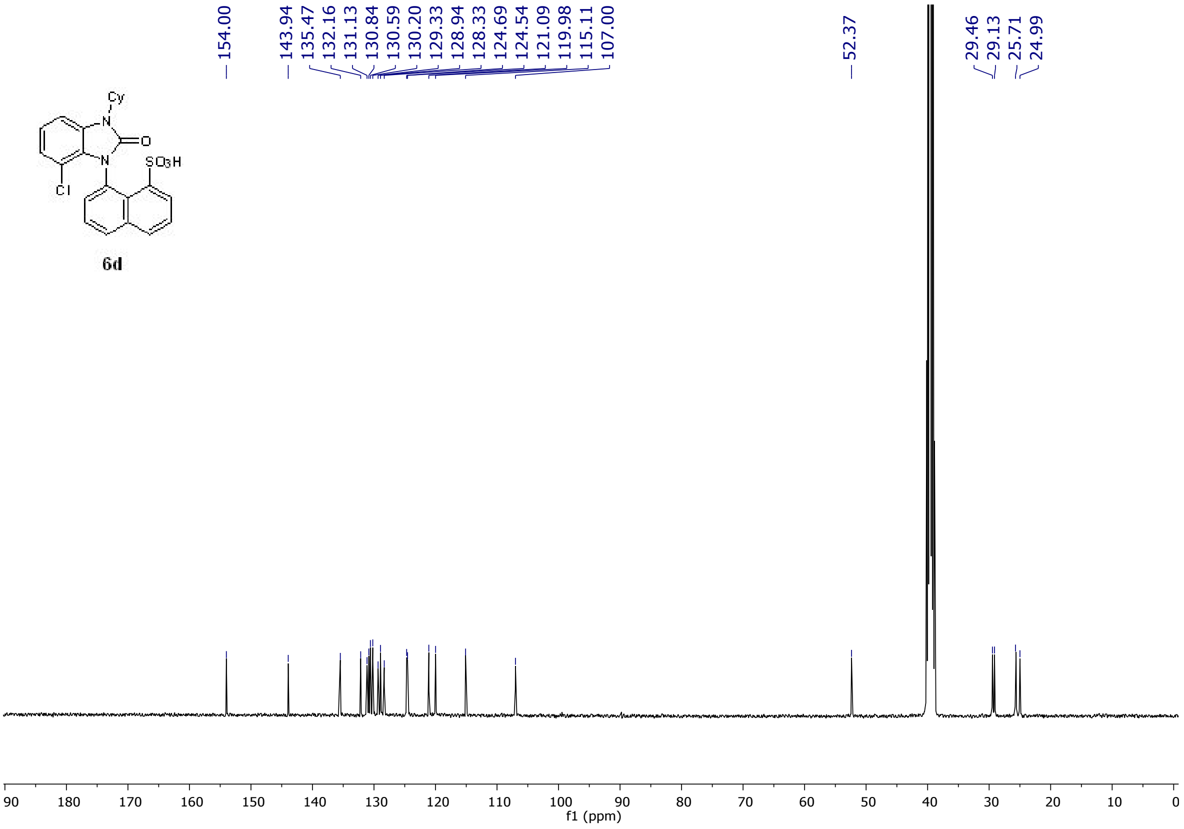

8-2-[(Dimethylamino)-3-isopropyl-*1H*-benzo[*d*]imidazol-3-ium-1-yl]naphthalene-1-sulfonate 7a

^{1}H NMR (500 MHz, $CDCl_3$)

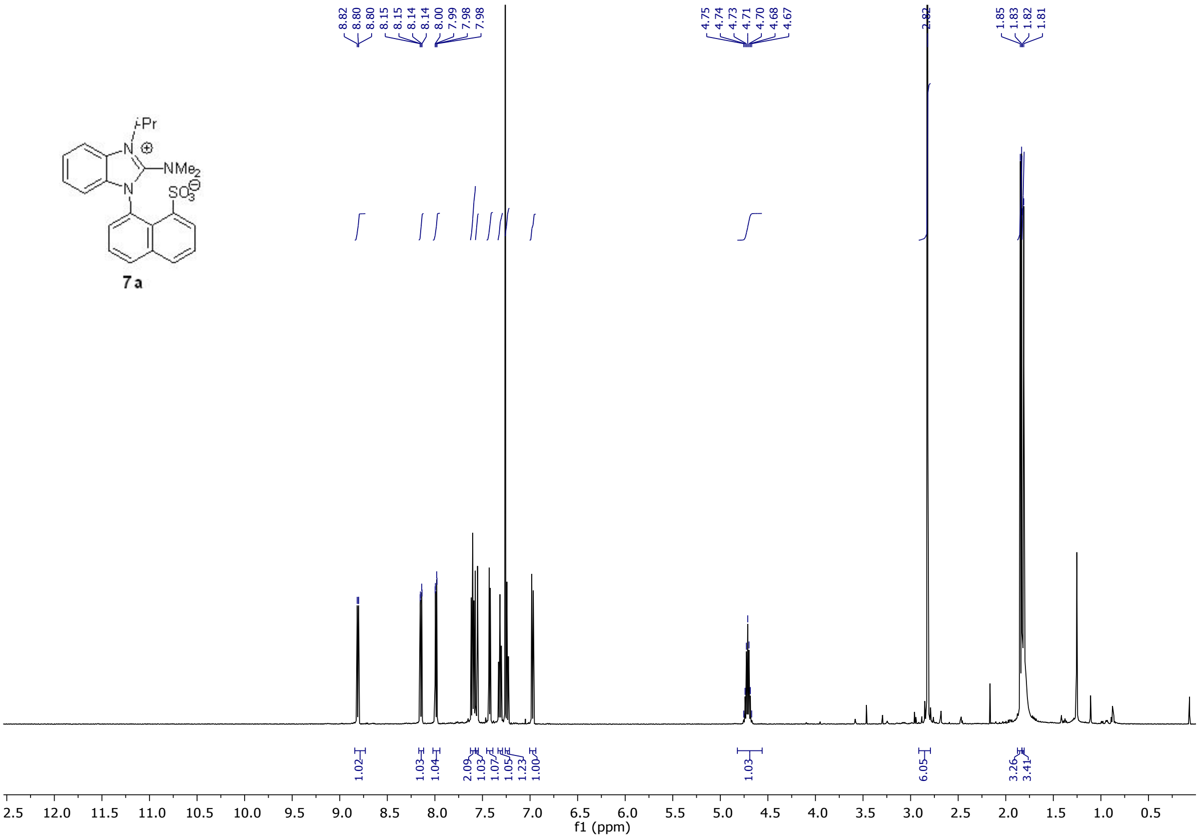

$^{13}C\{^{1}H\}$ NMR (126 MHz, $CDCl_3$)

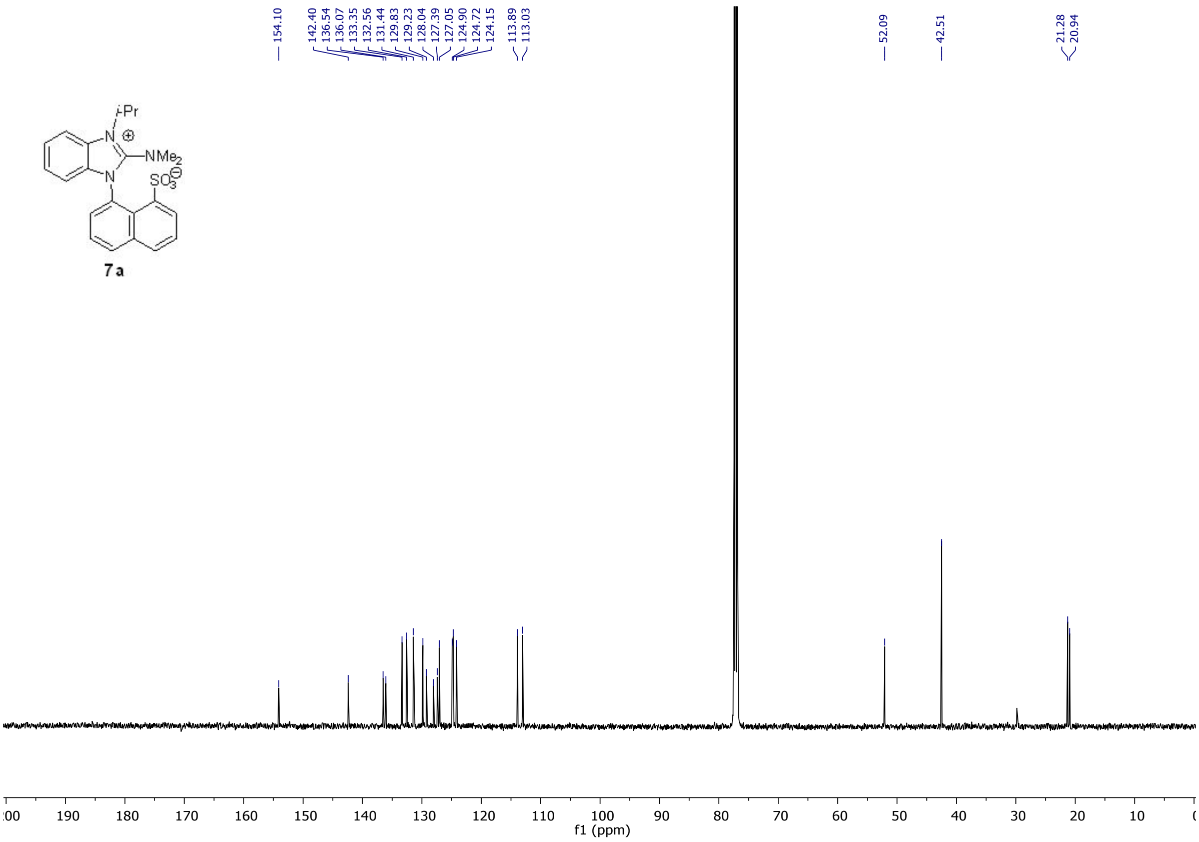

8-[3-Cyclohexyl-2-(dimethylamino)-*1H*-benzo[*d*]imidazol-3-ium-1-yl]naphthalene-1-sulfonate 7b

^{1}H NMR (500 MHz, $CDCl_3$)

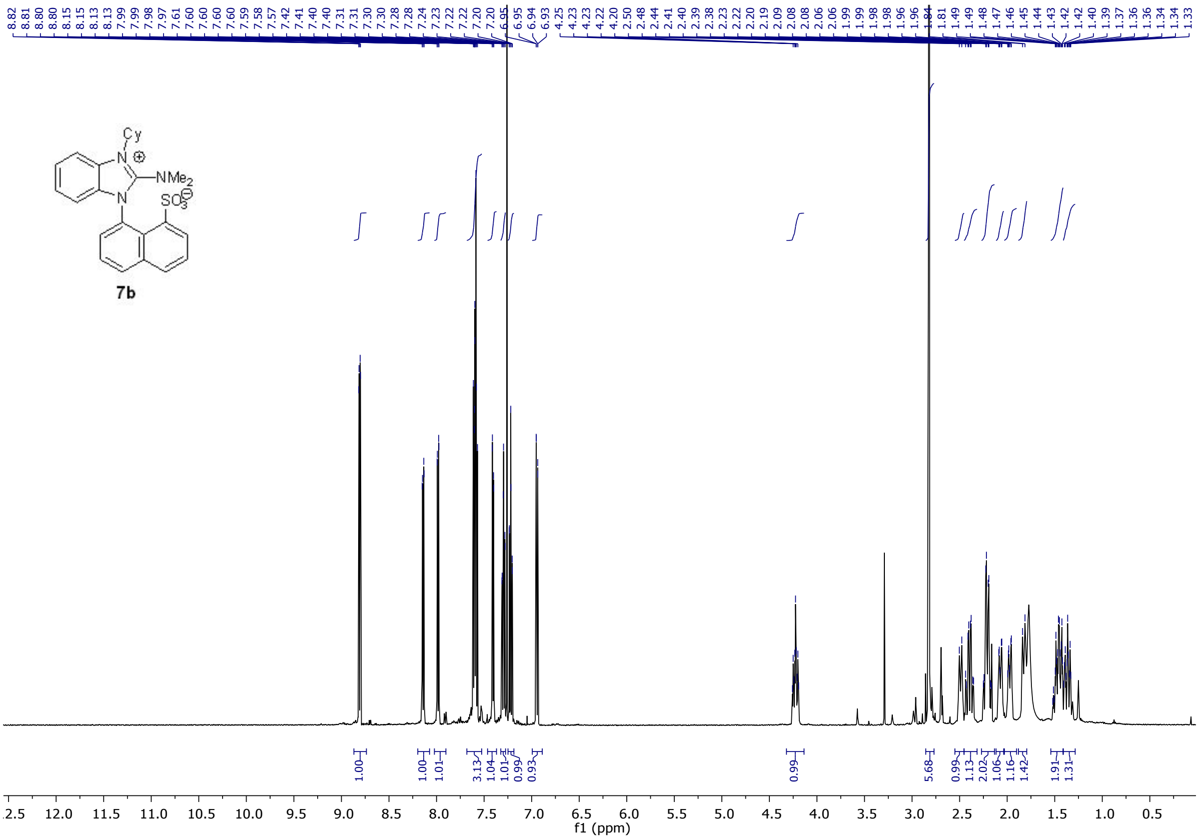

^{13}C{^{1}H} NMR (126 MHz, $CDCl_3$)

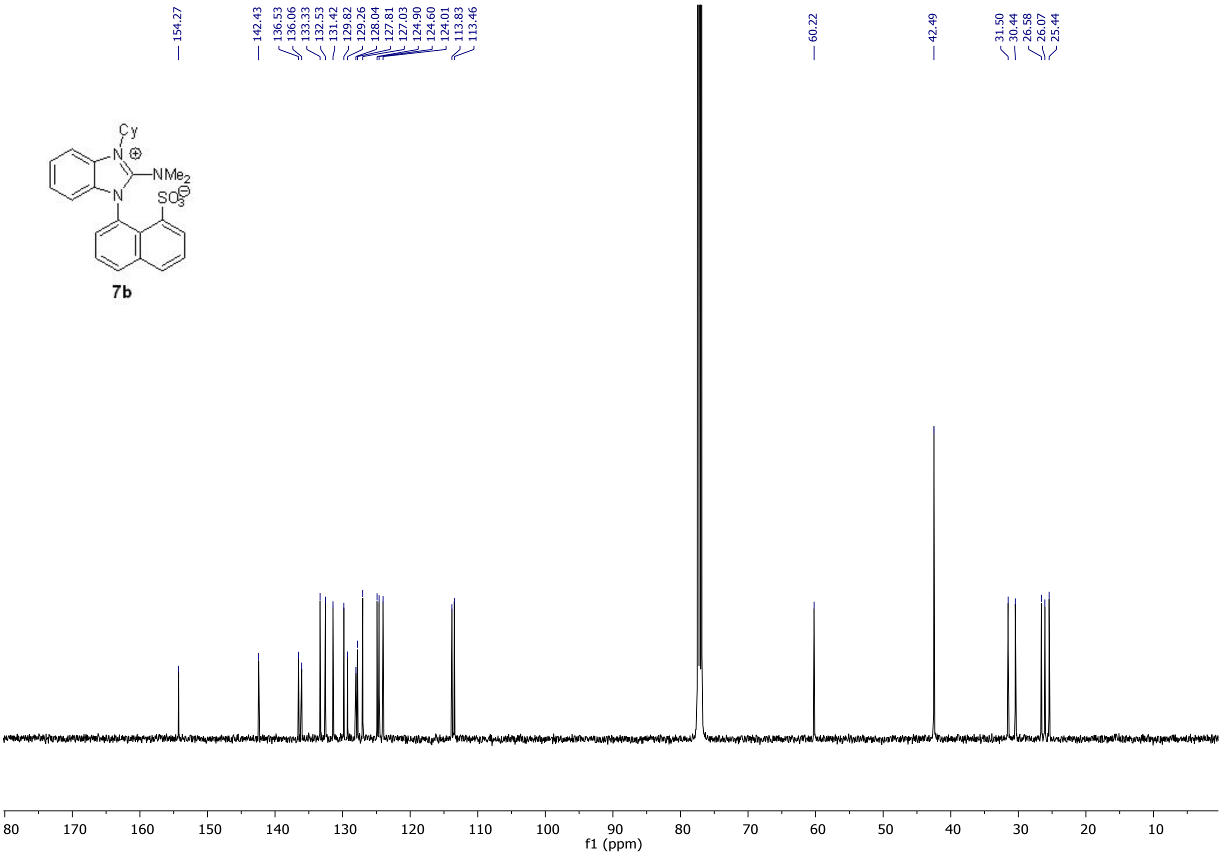

8-[7-Chloro-2-(dimethylamino)-3-isopropyl-1*H*-benzo[*d*]imidazol-3-ium-1-yl]naphthalene-1-sulfonate 7c

^{1}H NMR (500 MHz, DMSO-d_6)

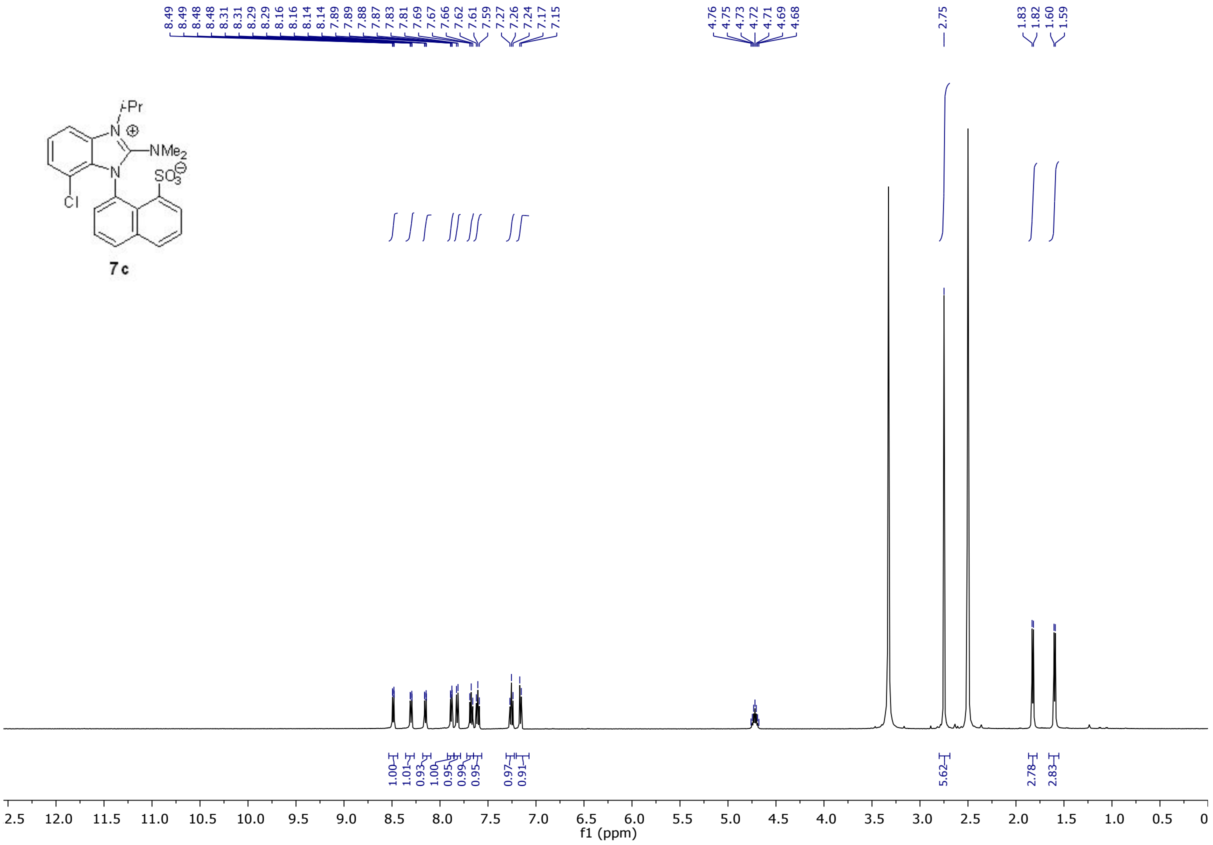

^{13}C{^{1}H} NMR (126 MHz, DMSO-d_6)

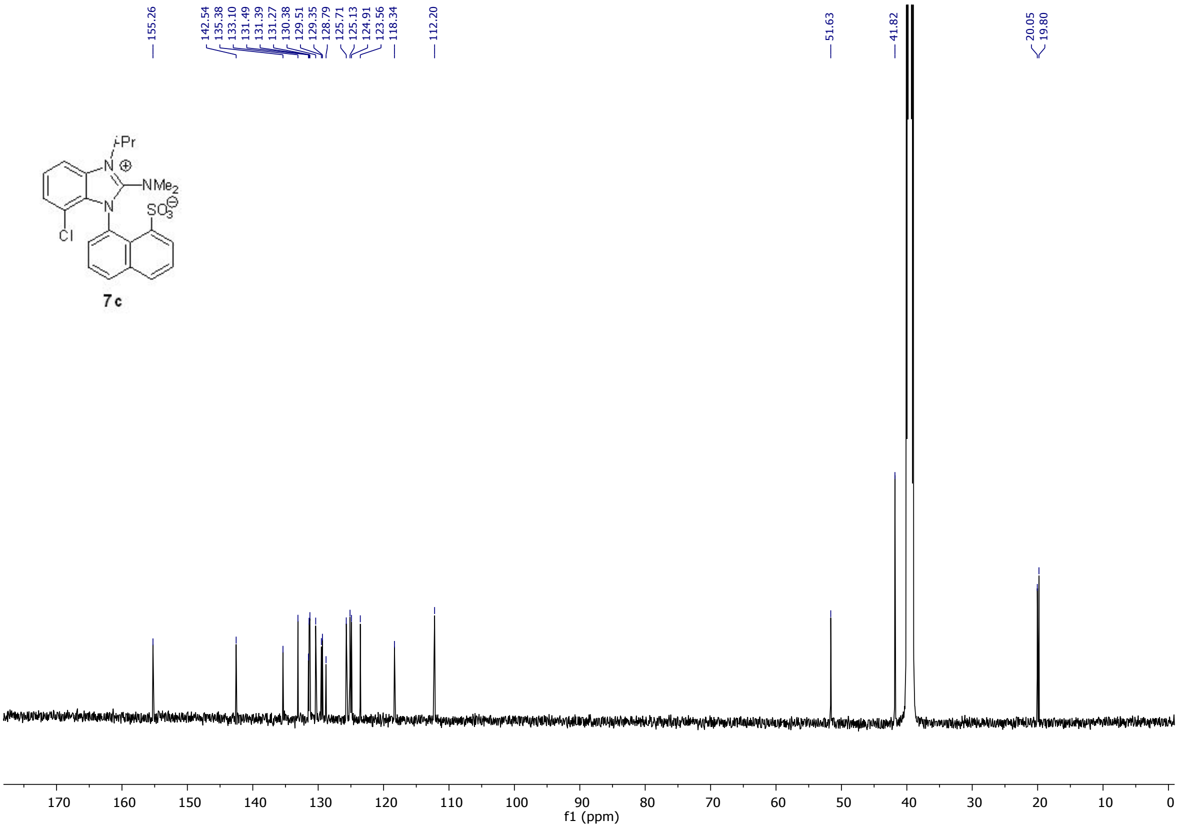

8-[7-Chloro-3-cyclohexyl-2-(dimethylamino)-1*H*-benzo[*d*]imidazol-3-ium-1-yl]naphthalene-1-sulfonate 7d

^{1}H NMR (500 MHz, DMSO-d_6)

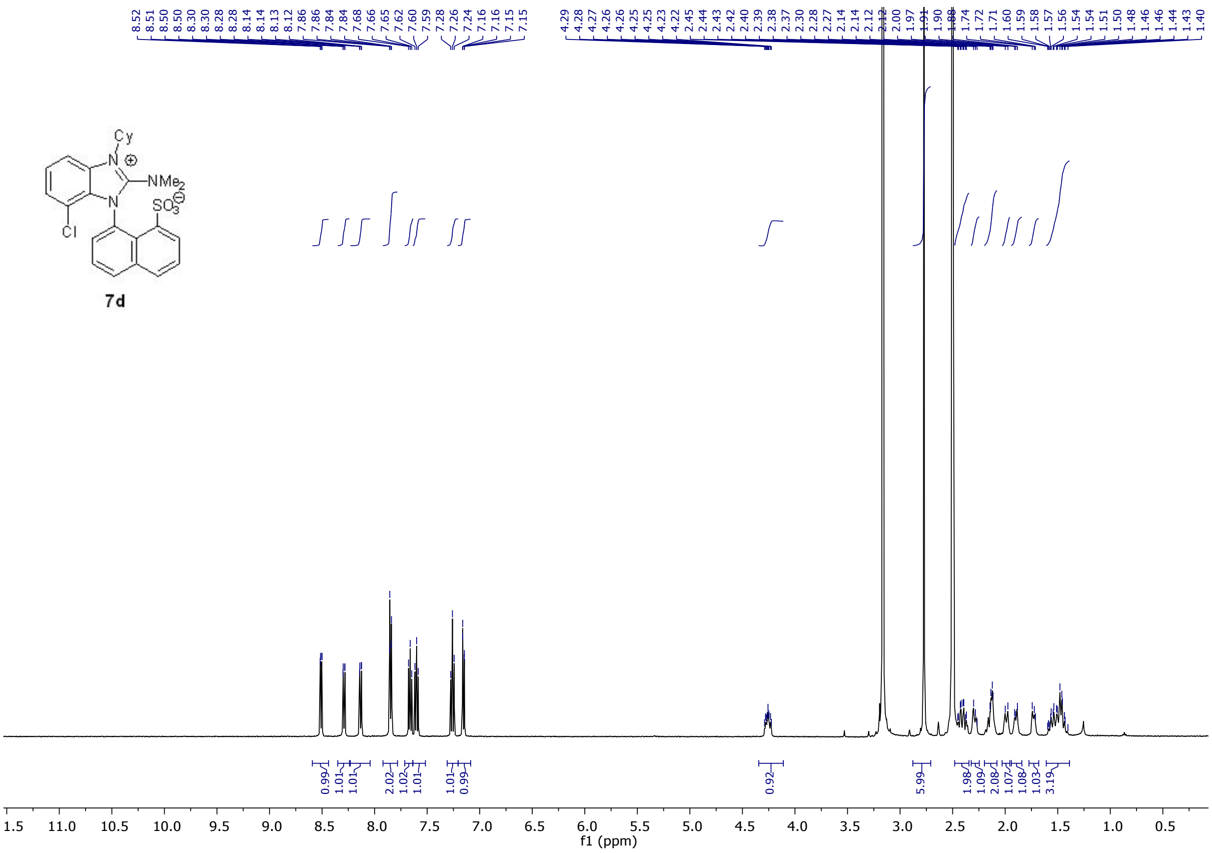

$^{13}C\{^{1}H\}$ NMR (126 MHz, DMSO-d_6)

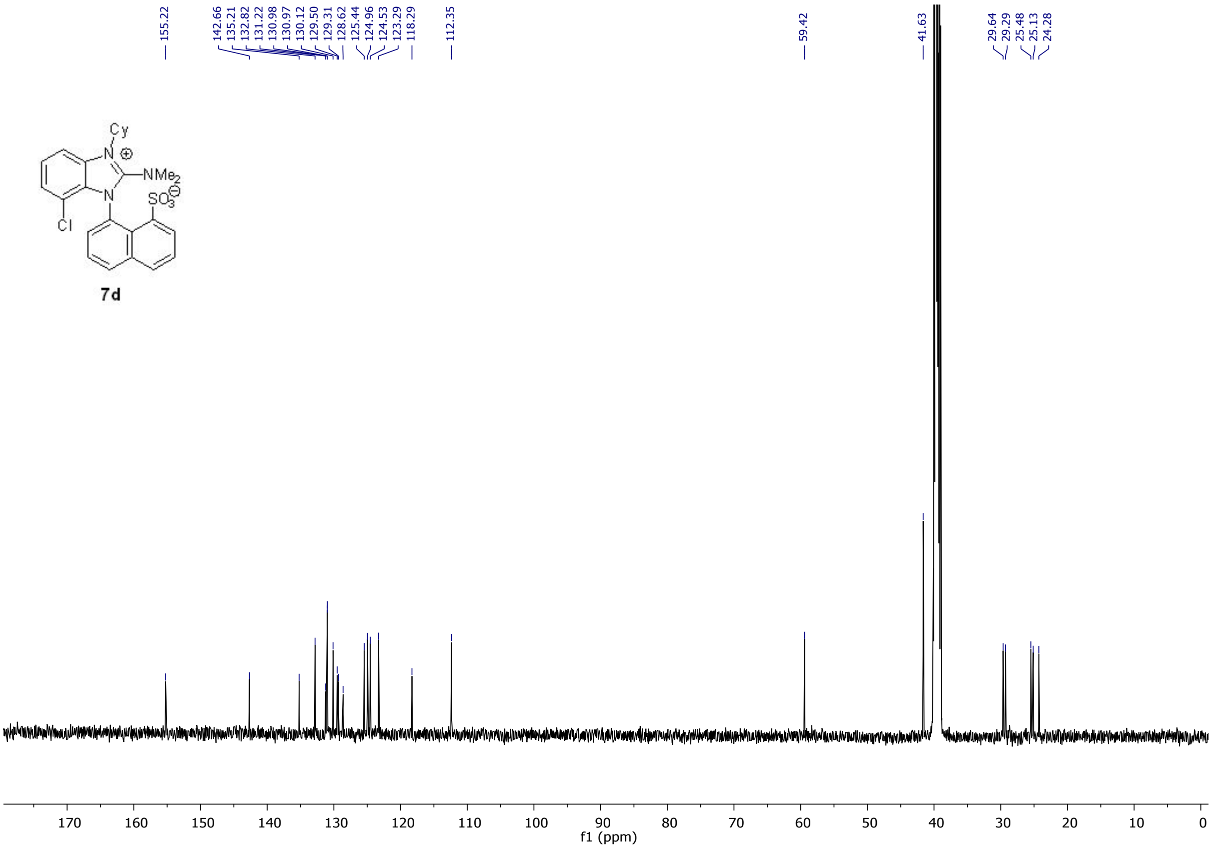

8-(2-Ethyl-1*H*-benzo[*d*]imidazol-1-yl)-*N*-((trifluoromethyl)sulfonyl)naphthalene-1-sulfonamide 9b

^{1}H NMR (400 MHz, DMSO-d_6)

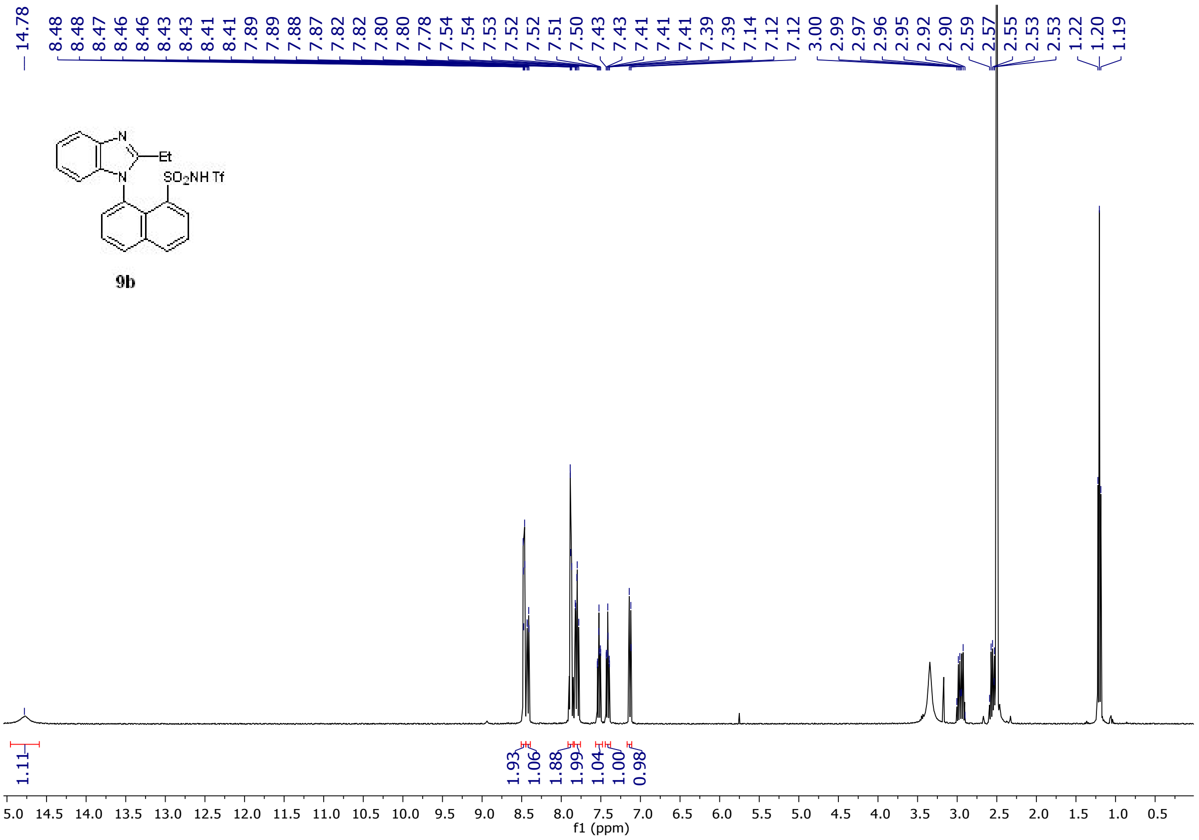

^{13}C{^{1}H} NMR (101 MHz, DMSO-d_6)

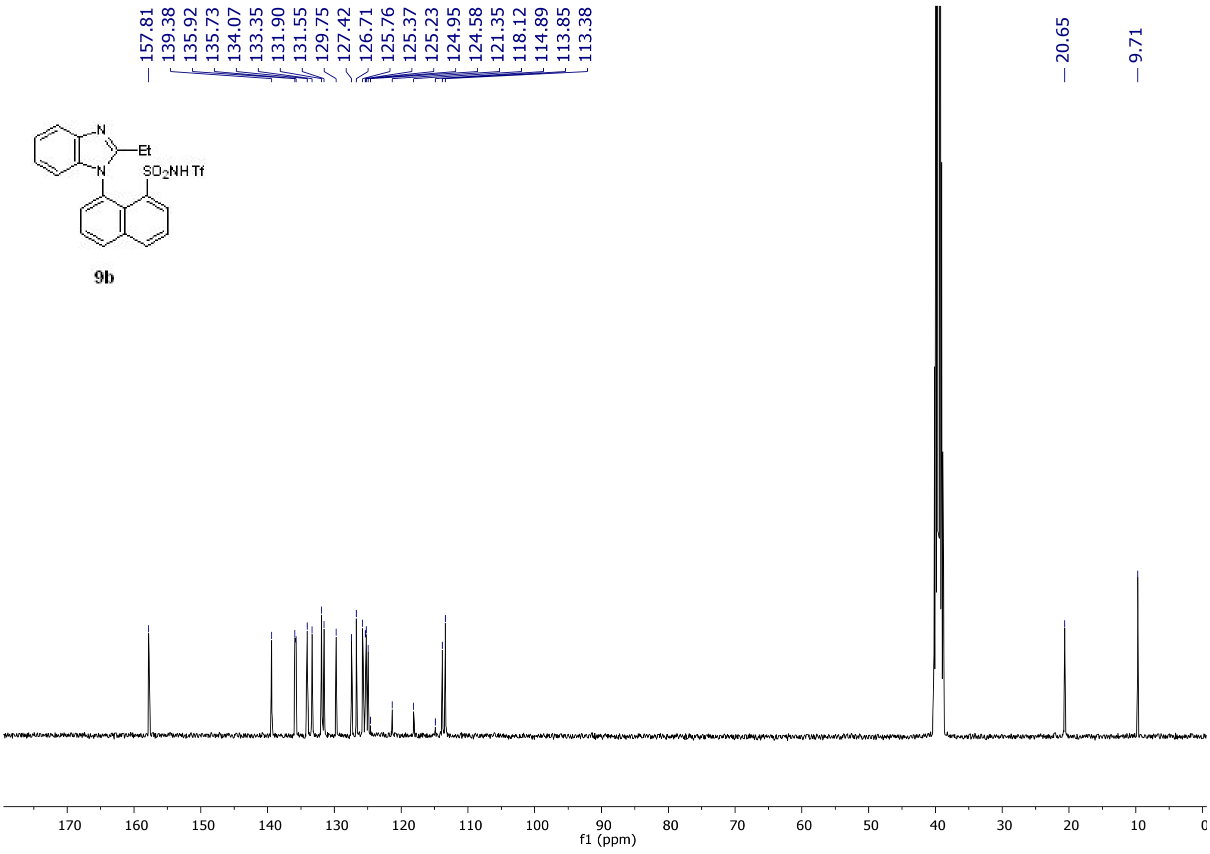

8-(7-Chloro-2-ethyl-1*H*-benzo[*d*]imidazol-1-yl)-*N*-((trifluoromethyl)sulfonyl)naphthalene-1-sulfonamide 9e

^{1}H NMR (400 MHz, DMSO-d_6)

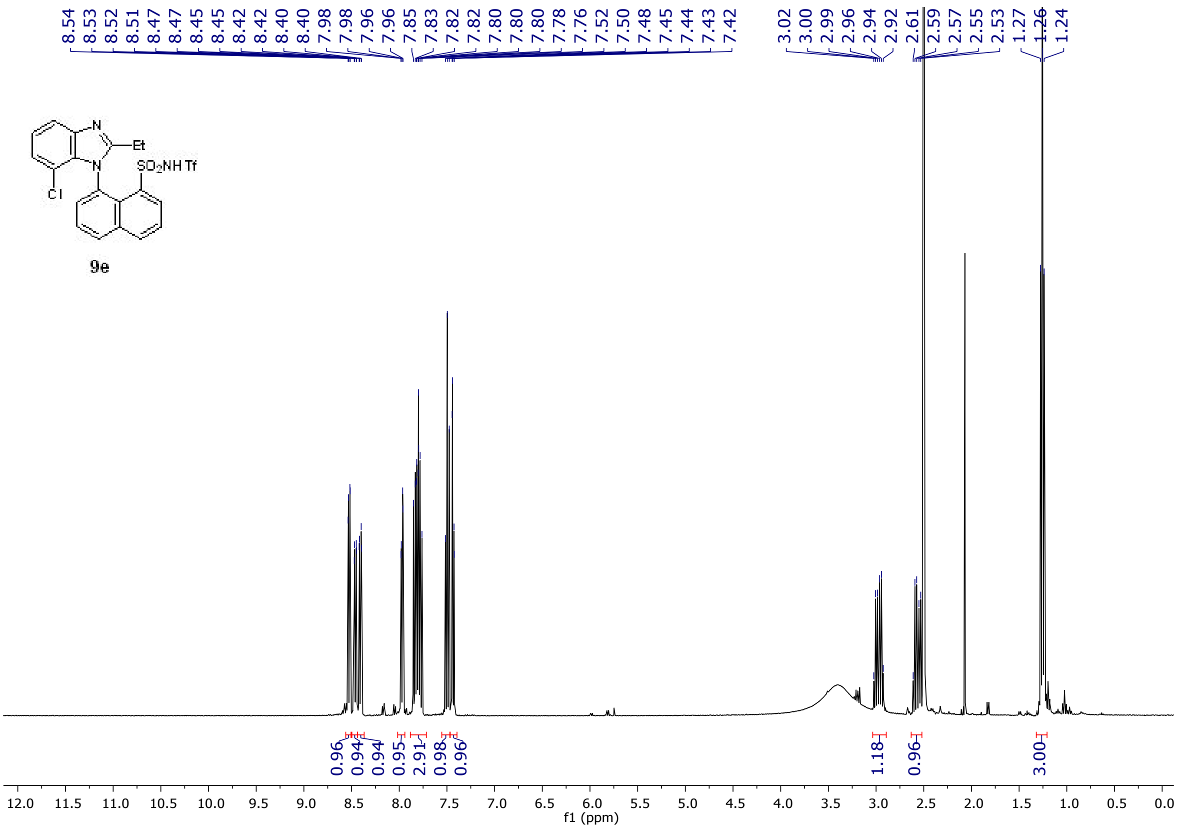

^{13}C{^{1}H} NMR (101 MHz, DMSO-d_6)

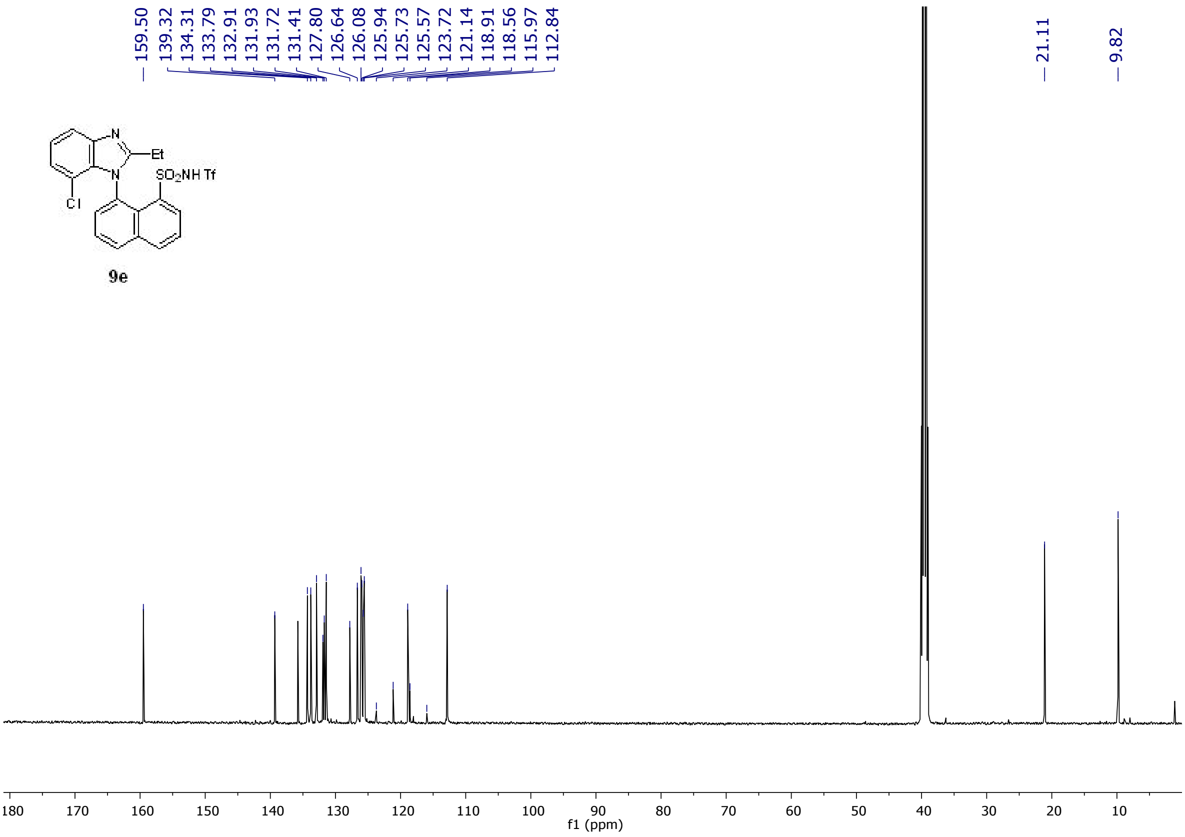

8-(3-Isopropyl-2-oxo-2,3-dihydro-1*H*-benzo[*d*]imidazol-1-yl)naphthalene-1-sulfonyl chloride 10a

^{1}H NMR (500 MHz, $CDCl_3$)

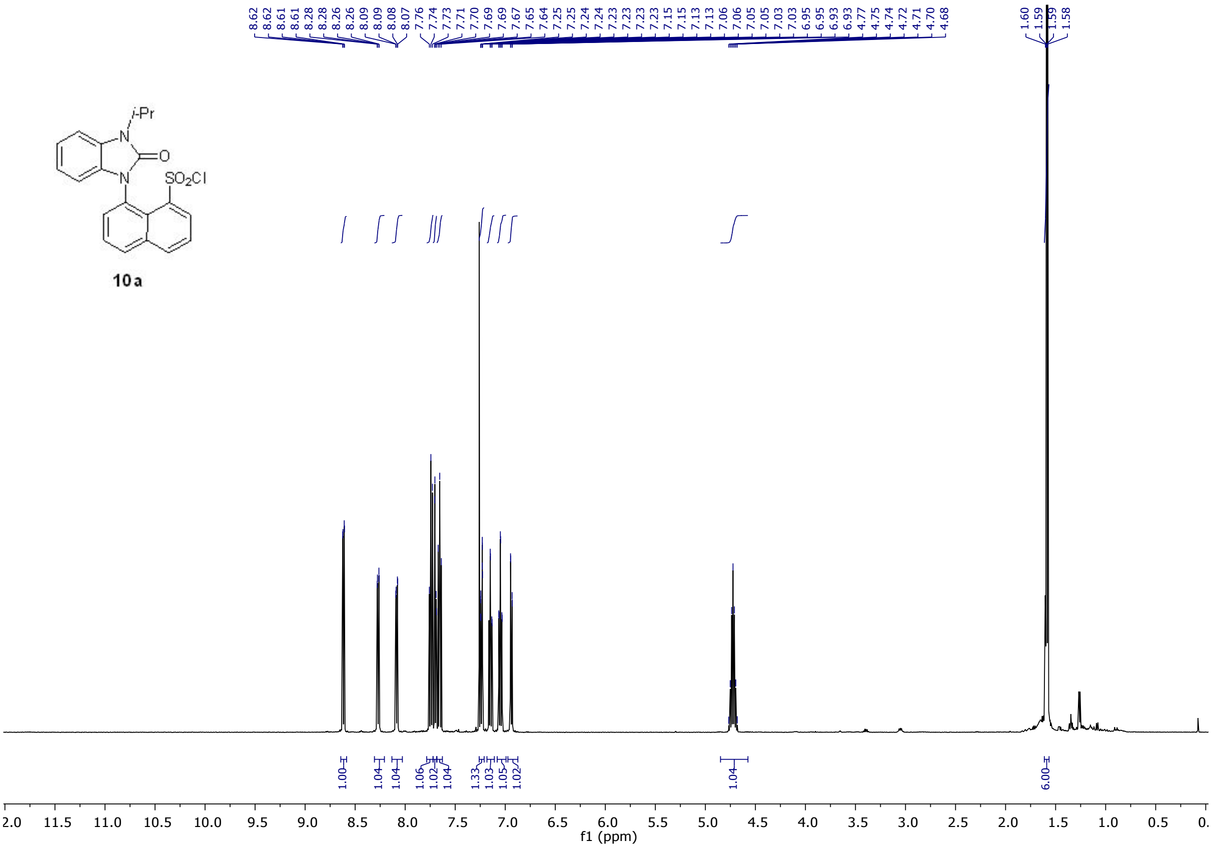

$^{13}C\{^{1}H\}$ NMR (126 MHz, $CDCl_3$)

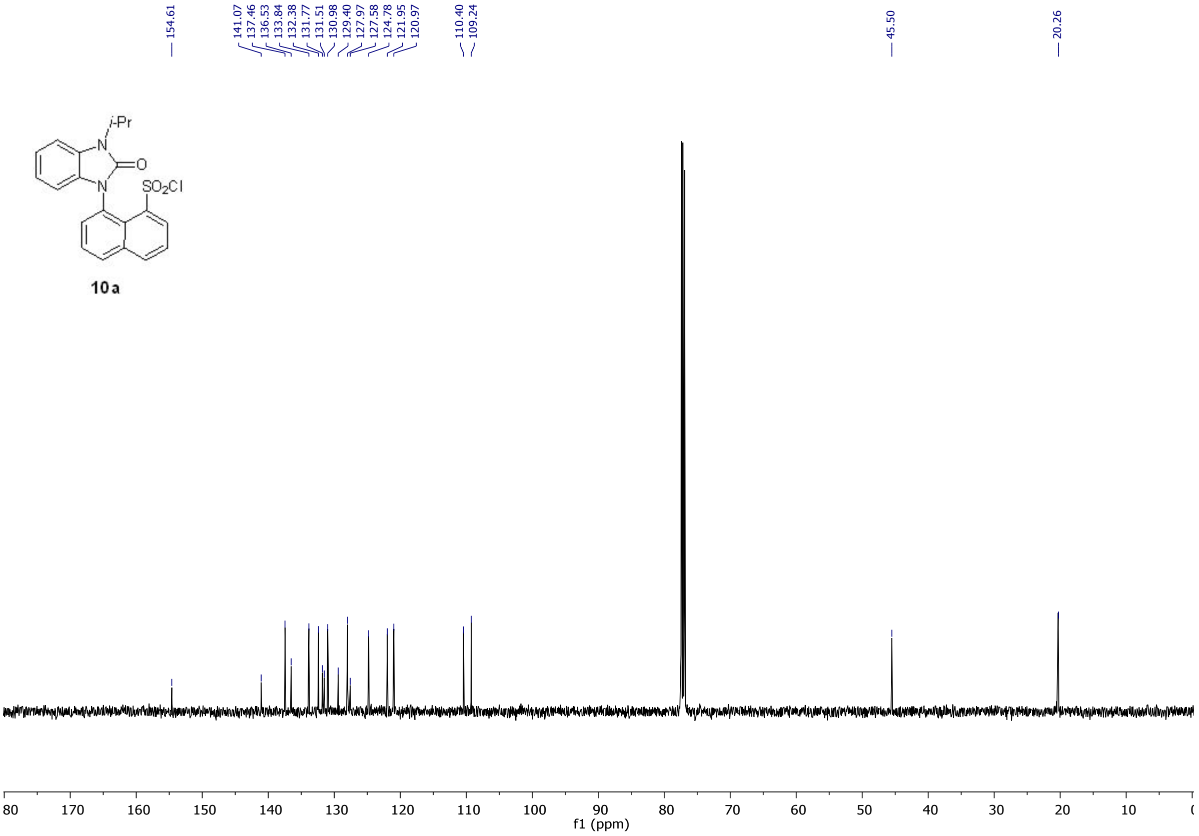

8-(3-Cyclohexyl-2-oxo-2,3-dihydro-1*H*-benzo[*d*]imidazol-1-yl)naphthalene-1-sulfonyl chloride 10b

^{1}H NMR (500 MHz, $CDCl_3$)

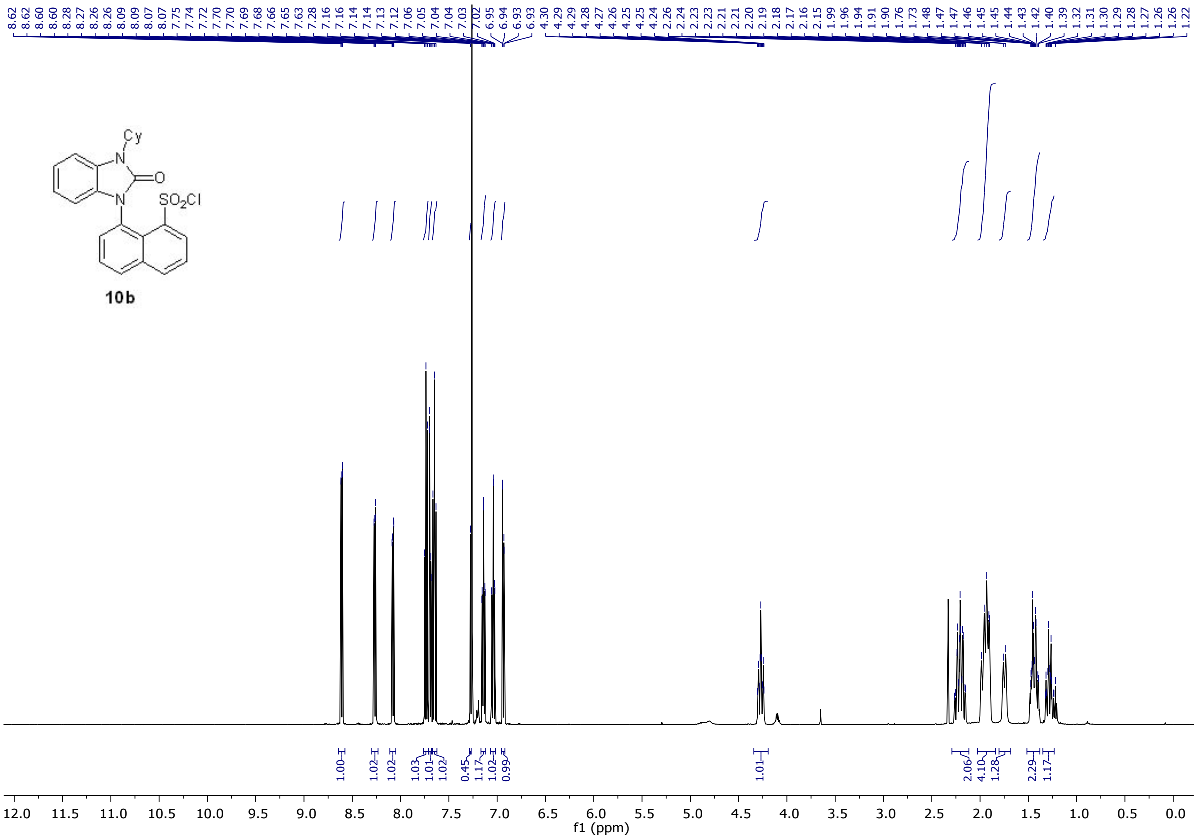

^{13}C{^{1}H} NMR (126 MHz, $CDCl_3$)

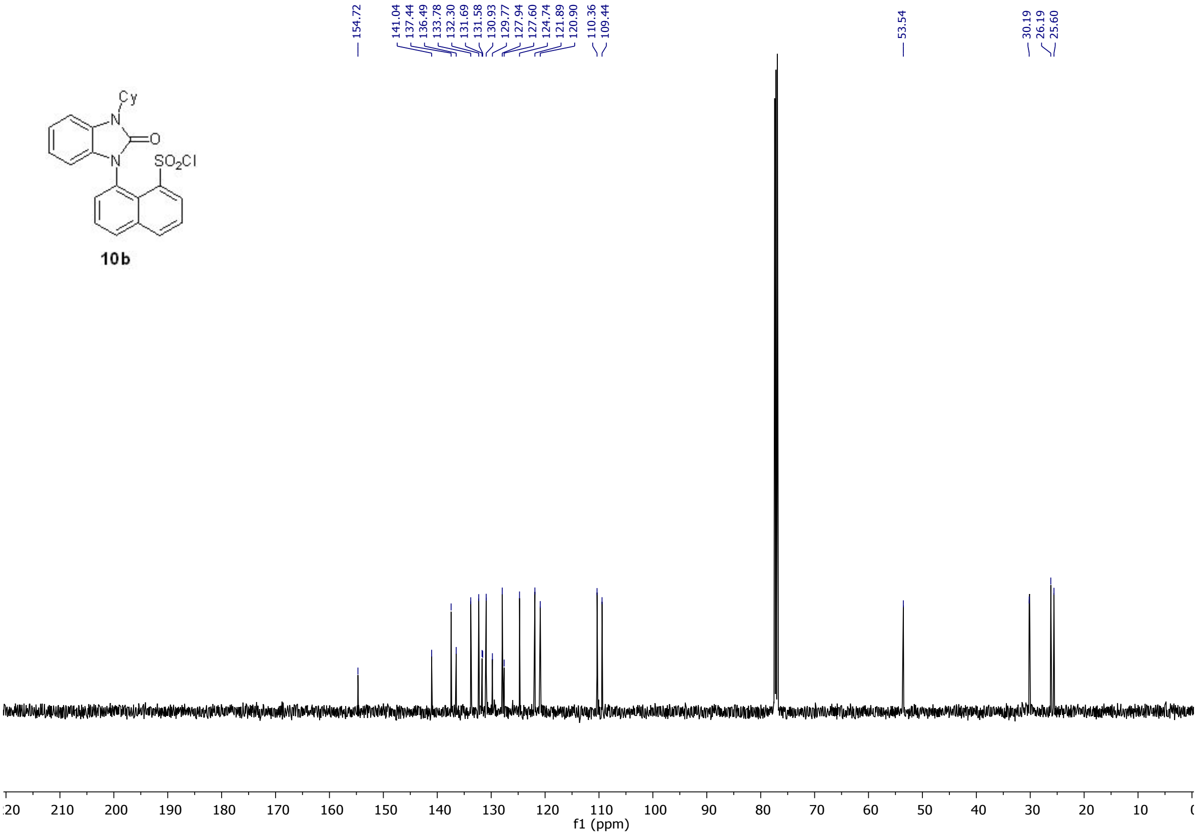

8-(7-Chloro-3-isopropyl-2-oxo-2,3-dihydro-1*H*-benzo[*d*]imidazol-1-yl)naphthalene-1-sulfonyl chloride 10c

^{1}H NMR (500 MHz, $CDCl_3$)

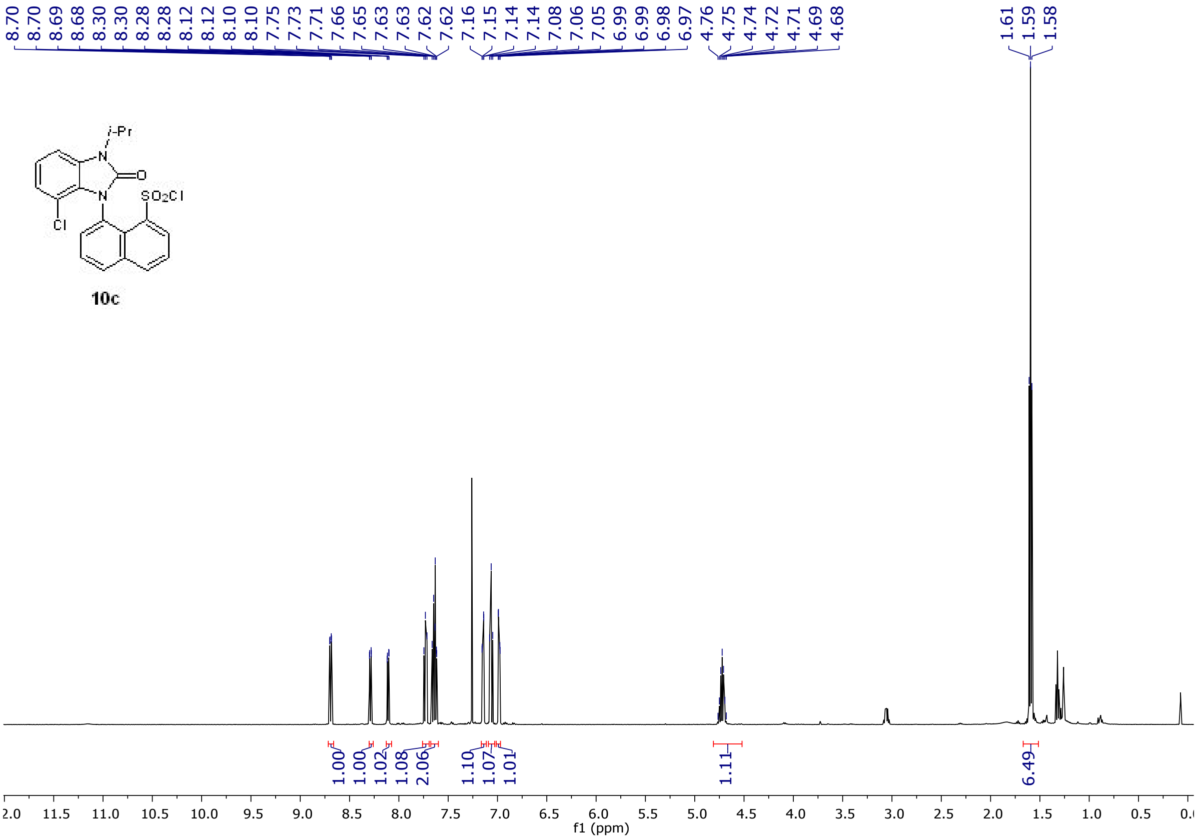

^{13}C{^{1}H} NMR (126 MHz, $CDCl_3$)

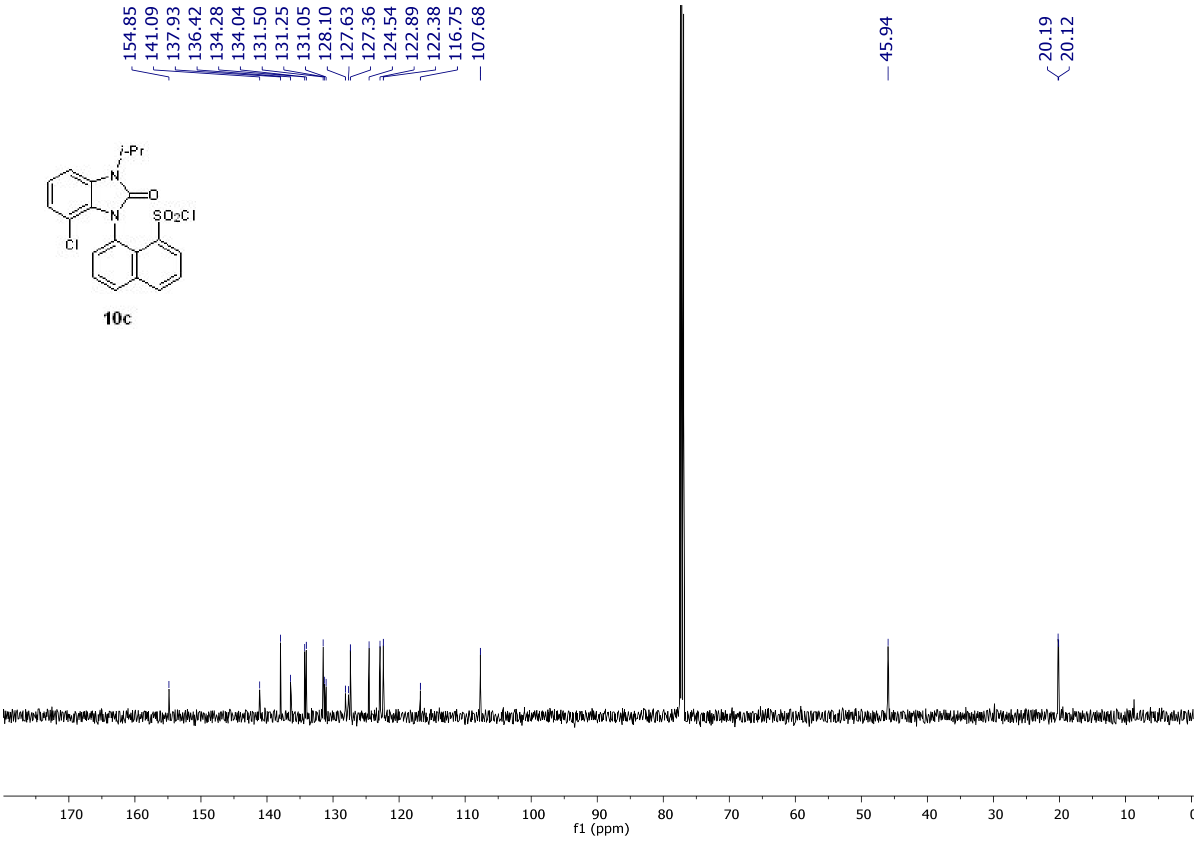

8-(7-Chloro-3-cyclohexyl-2-oxo-2,3-dihydro-1*H*-benzo[*d*]imidazol-1-yl)naphthalene-1-sulfonyl chloride 10d

^{1}H NMR (500 MHz, $CDCl_3$)

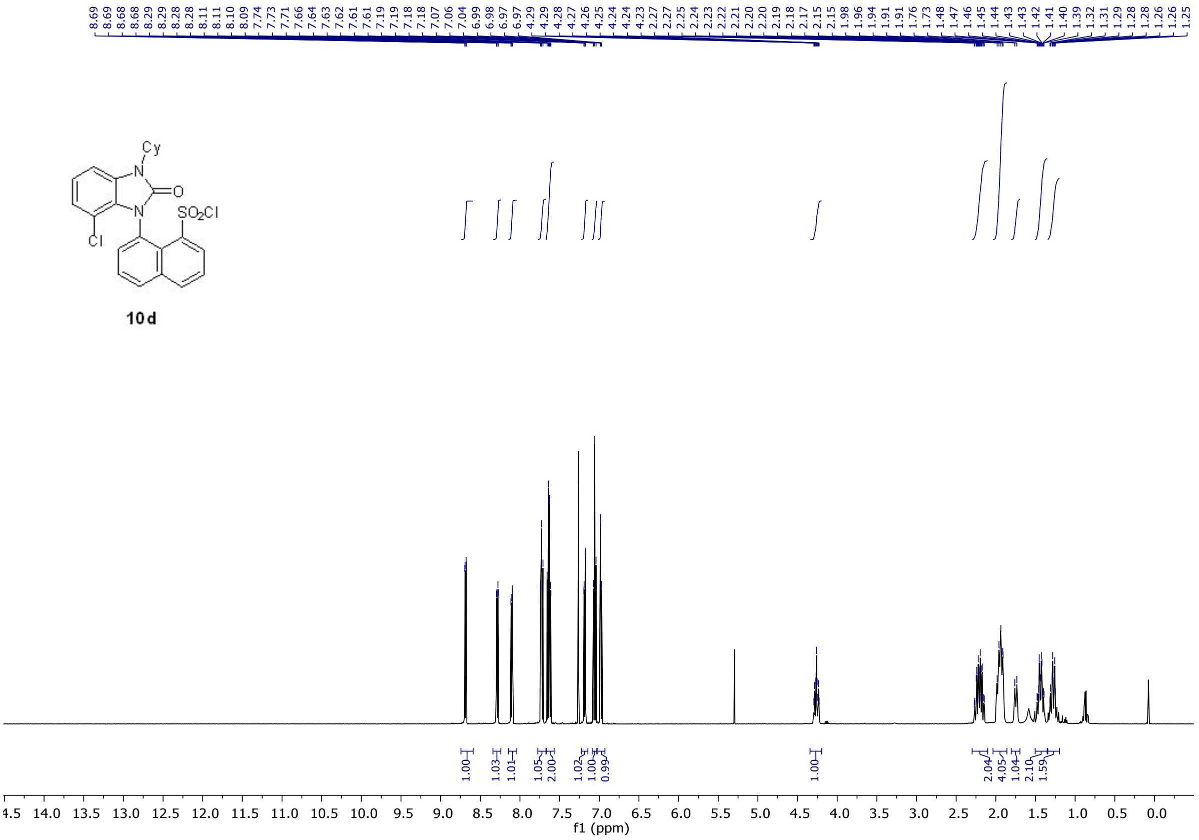

^{13}C{^{1}H} NMR (126 MHz, $CDCl_3$)

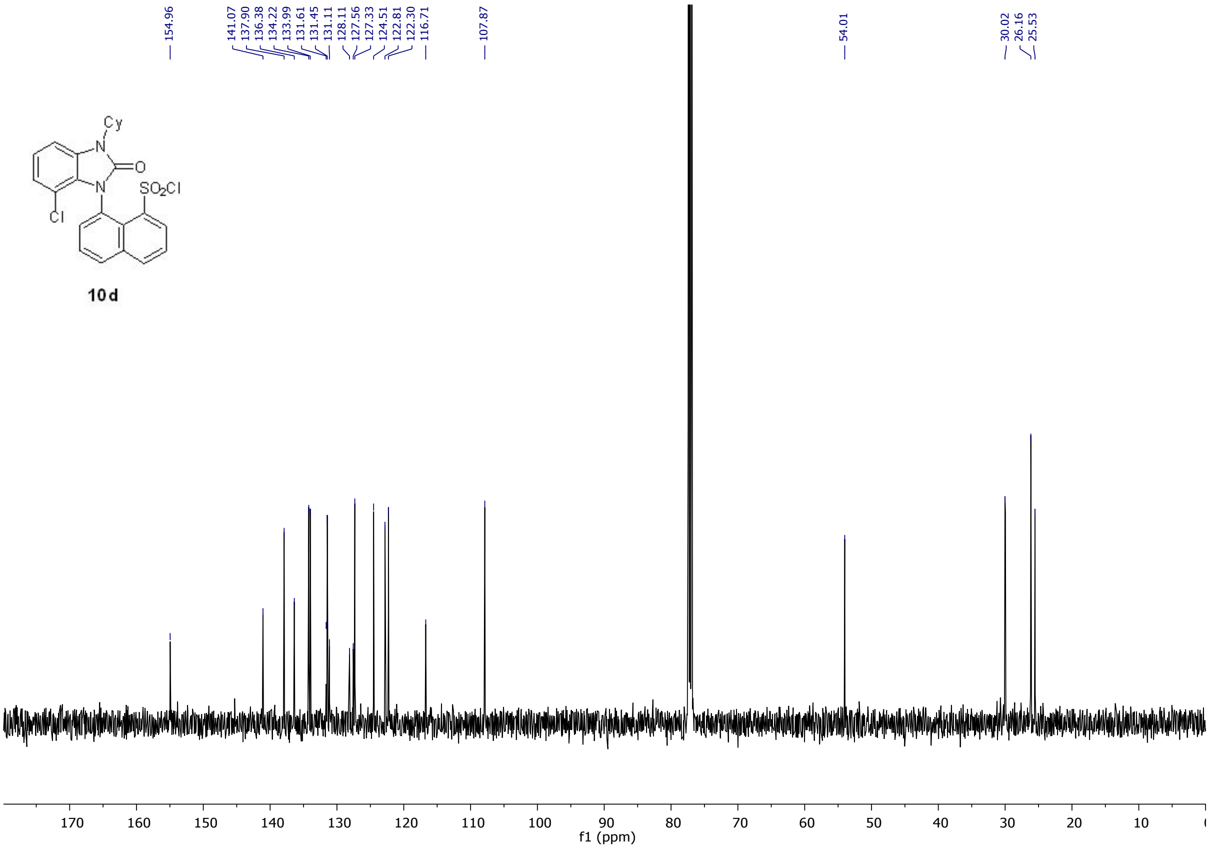

8-(3-Isopropyl-2-oxo-2,3-dihydro-1*H*-benzo[*d*]imidazol-1-yl)-*N*-(trifluoromethyl)sulfonyl)naphthalene-1-sulfonamide 11a

^{1}H NMR (500 MHz, DMSO-d_6)

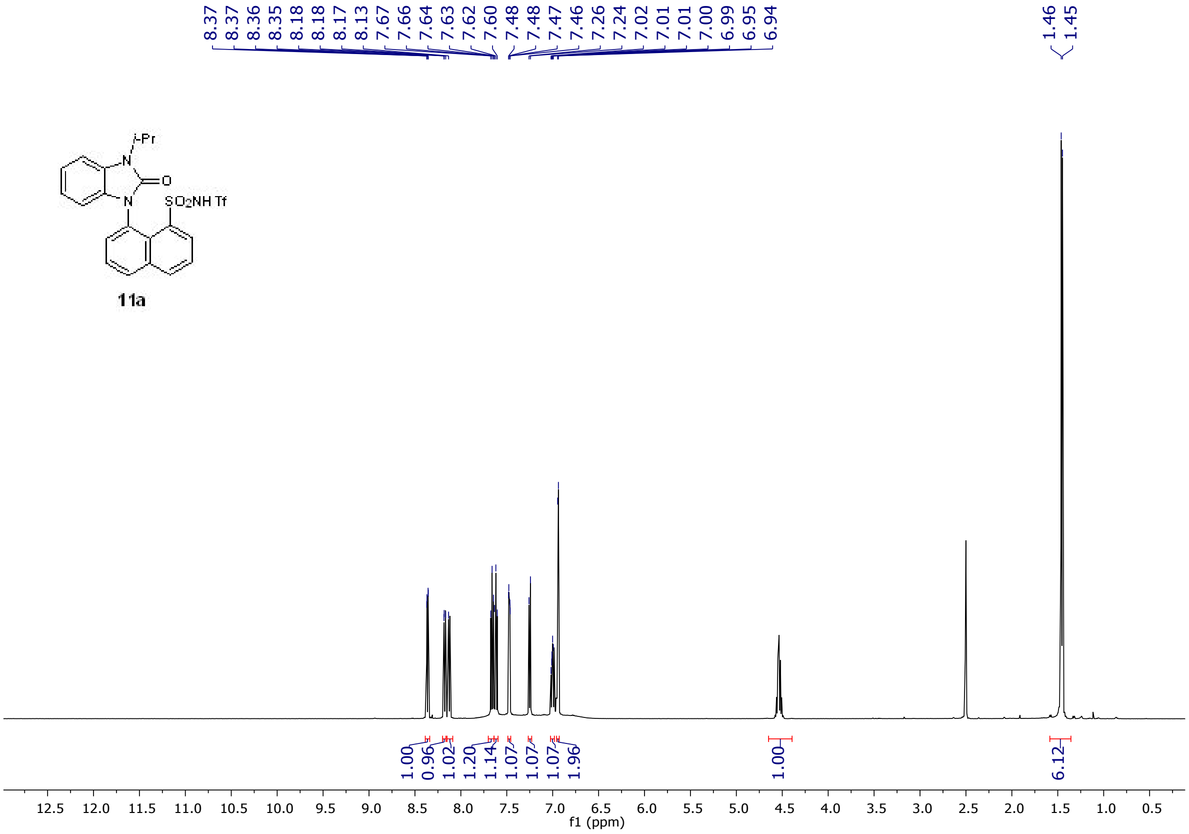

$^{13}C\{^{1}H\}$ NMR (126 MHz, DMSO-d_6)

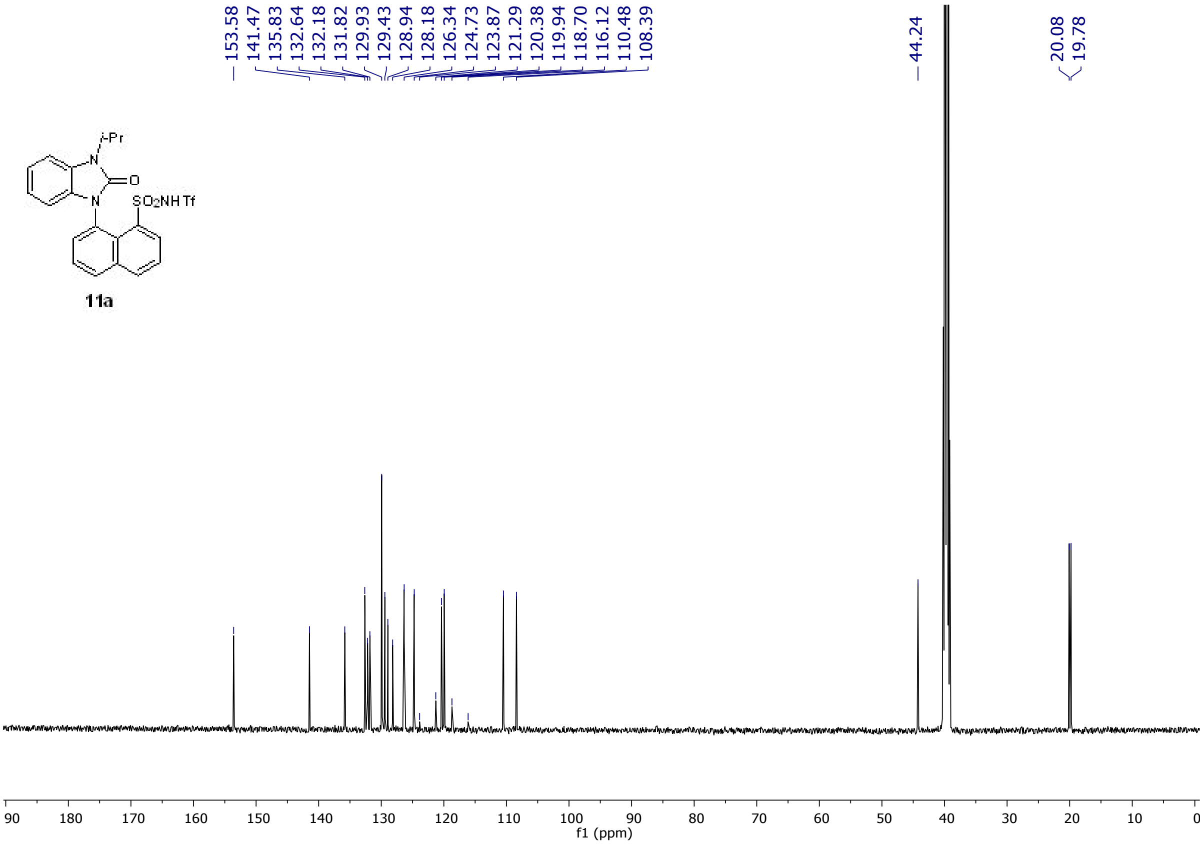

8-(3-Cyclohexyl-2-oxo-2,3-dihydro-1*H*-benzo[*d*]imidazol-1-yl)-*N*-((trifluoromethyl)sulfonyl)naphthalene-1-sulfonamide 11b

^{1}H NMR (400 MHz, DMSO-d_6)

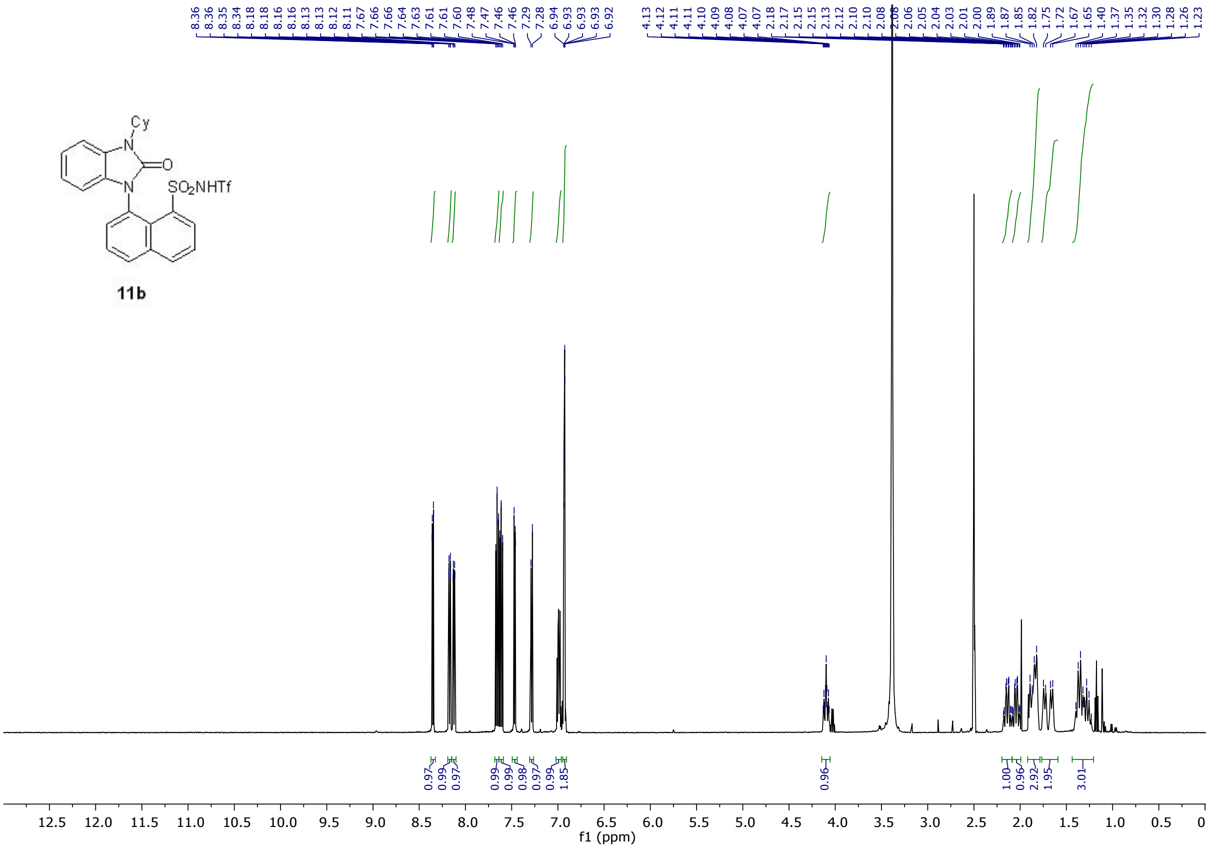

$^{13}C\{^{1}H\}$ NMR (126 MHz, DMSO-d_6)

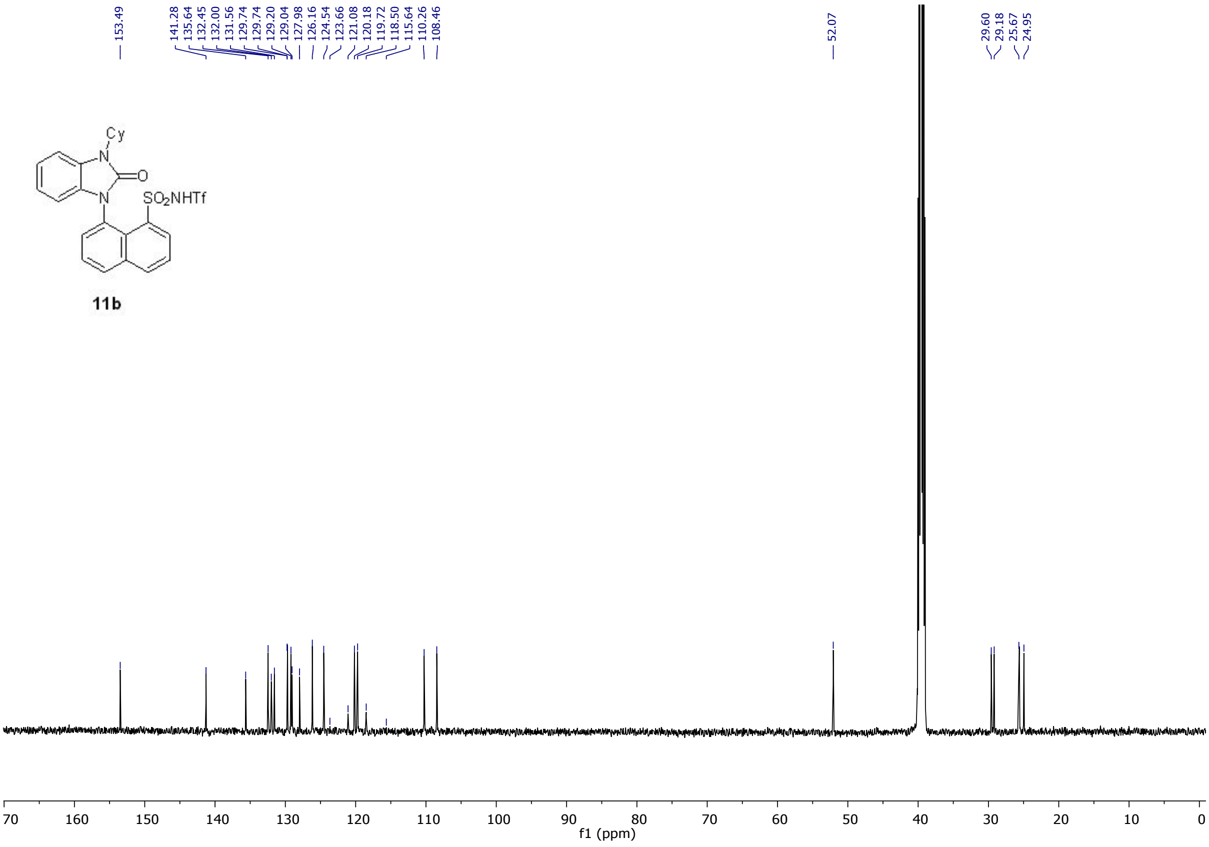

8-(7-Chloro-3-isopropyl-2-oxo-2,3-dihydro-1*H*-benzo[*d*]imidazol-1-yl)-*N*-((trifluoromethyl)sulfonyl)naphthalene-1-sulfonamide 11c

^{1}H NMR (400 MHz, DMSO-d_6)

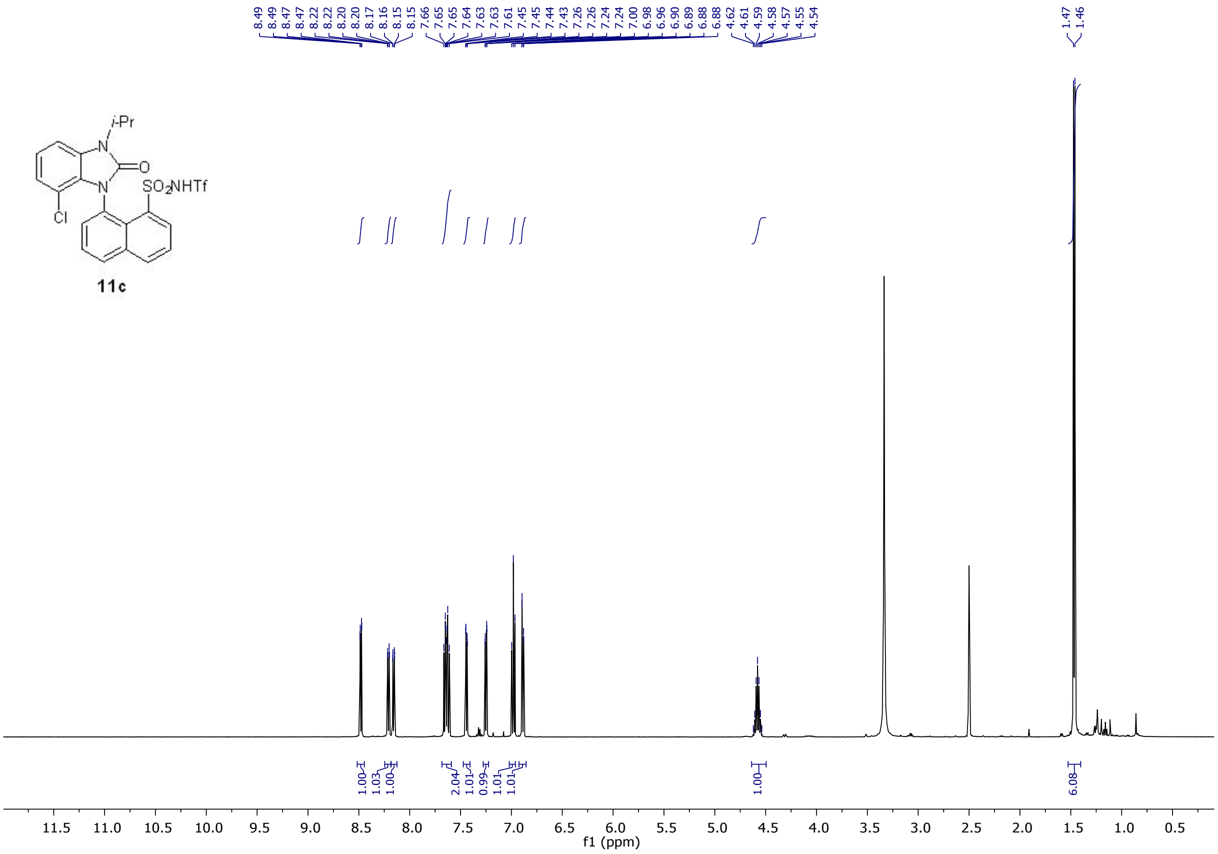

^{13}C{^{1}H} NMR (126 MHz, DMSO-d_6)

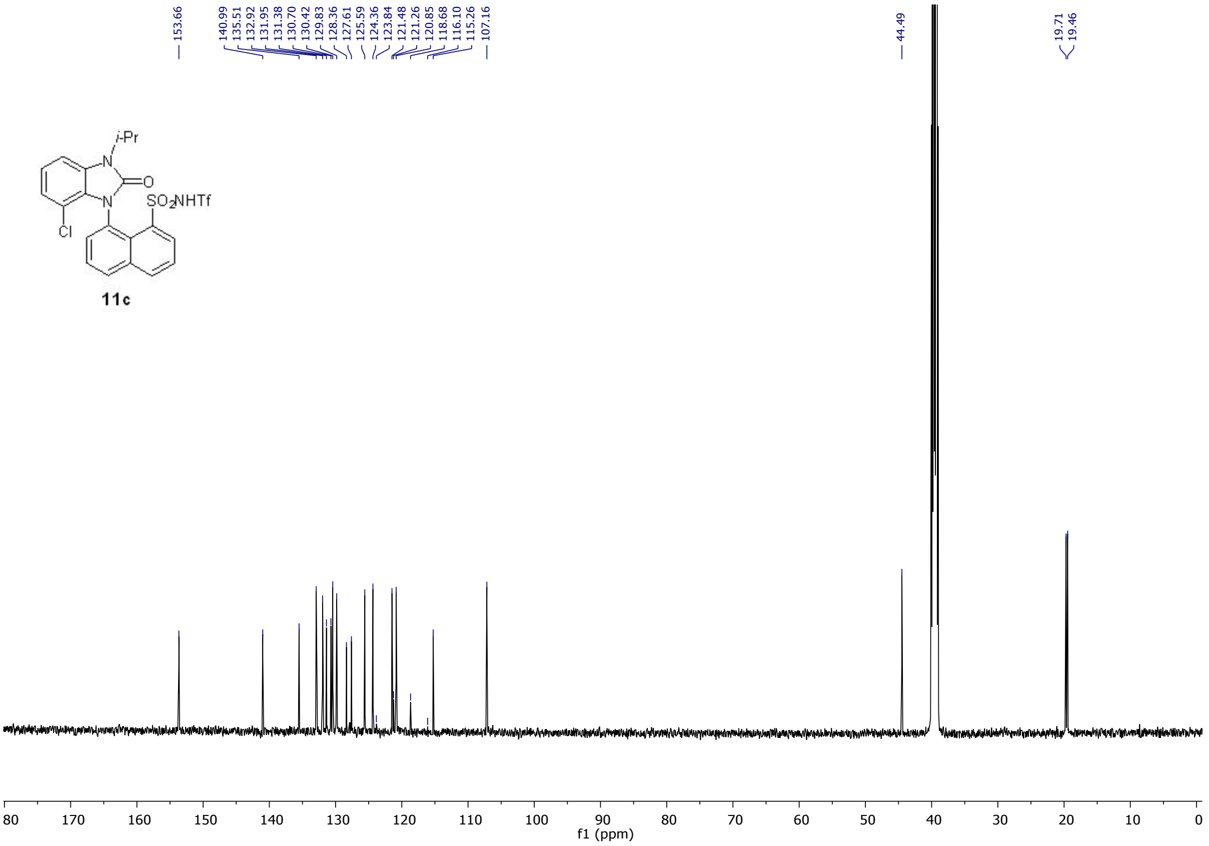

8-(7-chloro-3-cyclohexyl-2-oxo-2,3-dihydro-1*H*-benzo[*d*]imidazol-1-yl)-*N*-((trifluoromethyl)sulfonyl)naphthalene-1-sulfonamide 11d

^1H NMR (500 MHz, DMSO-d_6)

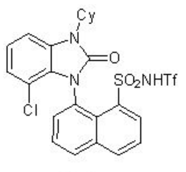

11d

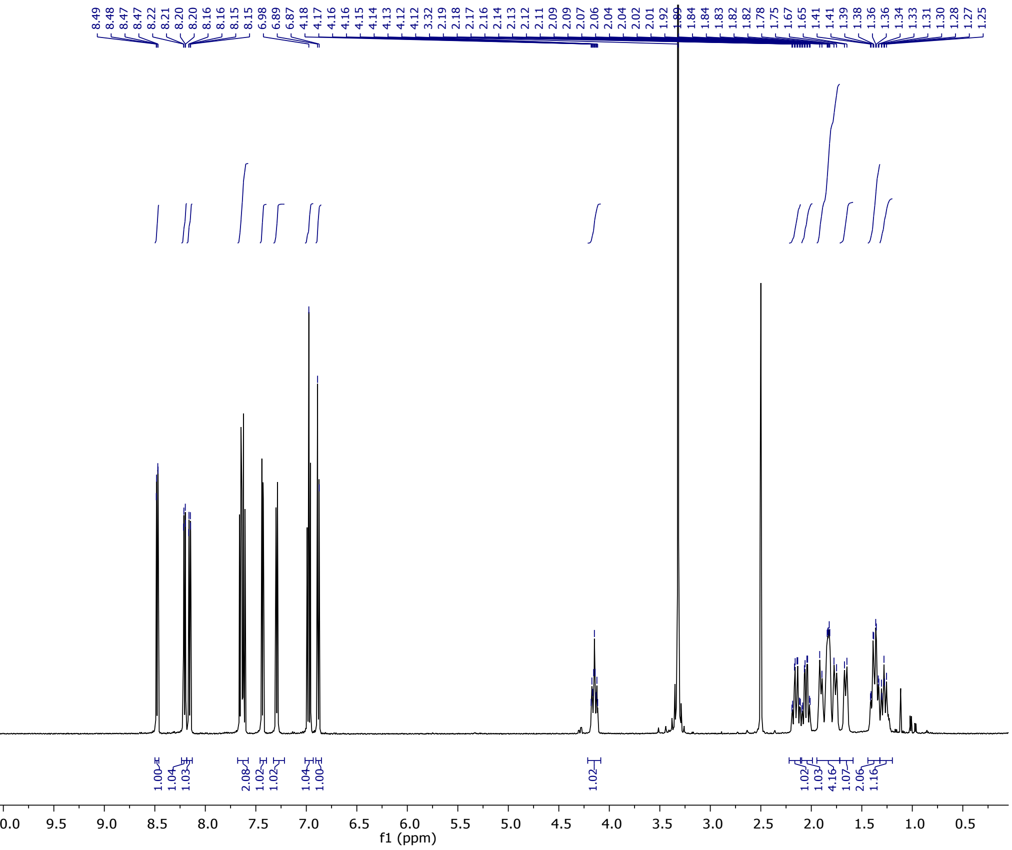

$^{13}C\{^1H\}$ NMR (126 MHz, DMSO-d_6)

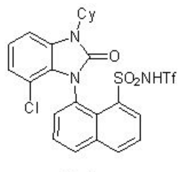

11d

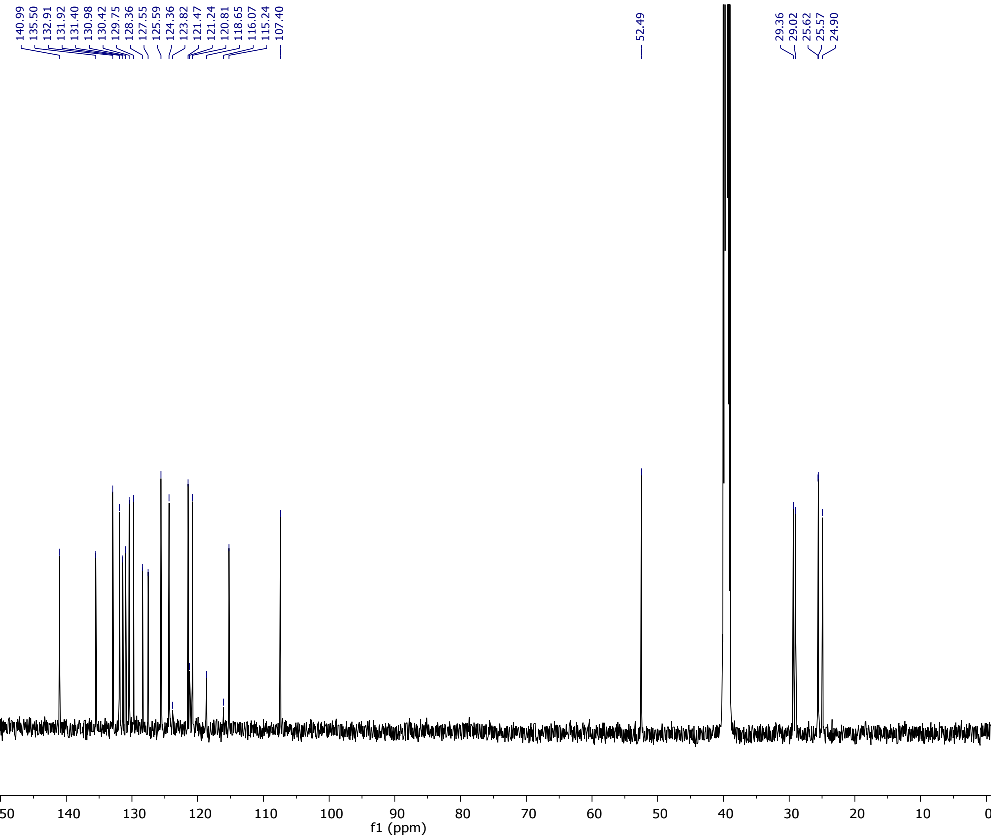

In-Silico Calculated pKa of Sulfonic Acid 6c

The pK_a of compound **6c** was calculated starting from the X-ray geometry of the anion **6c**, optimizing the geometry and calculating vibrational frequency analysis at the density functional theory level (M06-2X/6-31++G**), both for the protonated and anion states. Single point calculations at the M06-2X/6-311++G(2df,2p) followed on the optimized geometry. All calculations used SMD solvation model of Truhlar and co-workers with default parameters for ethanol as a solvent (Marenich, A. V.; Cramer, C. J.; Truhlar, D. G. *J. Phys. Chem. B* **2009**, *113*(18), 6378−6396) The calculations were performed for *p*-toluenesulfonic acid (reference) and **6c** compound, and the pK_a of compound **6c** was calculated using the proton-exchange method (see, e.g., Yang, C.; Xue, X.; Jin, J.; Li, X.; Cheng, J. *J. Org. Chem.* **2013**, *78*(14), 7076−7085) with *p*-toluenesulfonic acid as a reference acid (experimental pK_a = -0.9). All calculations were performed using the Gaussian 09 program [Gaussian 09, Revision A.02, M. J. Frisch, G. W. Trucks, H. B. Schlegel, G. E. Scuseria, M. A. Robb, J. R. Cheeseman, G. Scalmani, V. Barone, G. A. Petersson, H. Nakatsuji, X. Li, M. Caricato, A. Marenich, J. Bloino, B. G. Janesko, R. Gomperts, B. Mennucci, H. P. Hratchian, J. V. Ortiz, A. F. Izmaylov, J. L. Sonnenberg, D. Williams-Young, F. Ding, F. Lipparini, F. Egidi, J. Goings, B. Peng, A. Petrone, T. Henderson, D. Ranasinghe, V. G. Zakrzewski, J. Gao, N. Rega, G. Zheng, W. Liang, M. Hada, M. Ehara, K. Toyota, R. Fukuda, J. Hasegawa, M. Ishida, T. Nakajima, Y. Honda, O. Kitao, H. Nakai, T. Vreven, K. Throssell, J. A. Montgomery, Jr., J. E. Peralta, F. Ogliaro, M. Bearpark, J. J. Heyd, E. Brothers, K. N. Kudin, V. N. Staroverov, T. Keith, R. Kobayashi, J. Normand, K. Raghavachari, A. Rendell, J. C. Burant, S. S. Iyengar, J. Tomasi, M. Cossi, J. M. Millam, M. Klene, C. Adamo, R. Cammi, J. W. Ochterski, R. L. Martin, K. Morokuma, O. Farkas, J. B. Foresman, and D. J. Fox, Gaussian, Inc., Wallingford CT, 2016.].

Cartesian coordinates (Angstrom) of 6c anion, optimized at the M06-2X/6-31++G level.**

44

S	5.08672	6.42333	15.87485
Cl	6.99185	4.2264	19.4062
O	4.06799	5.55909	15.22811
O	6.23008	5.67206	16.44304
O	1.8972	4.55929	17.08263
O	5.52784	7.51009	14.96158
N	4.07509	4.27314	17.86135
N	3.26524	2.78431	16.42631
C	2.96351	3.93727	17.10229
C	5.05031	3.29557	17.70191
C	4.01033	6.66886	18.55469
C	3.68369	7.559	19.63118
C	4.32005	7.27489	17.28393
C	6.57108	1.11333	16.9071
H	7.18145	0.26872	16.6064
C	3.55882	8.95732	19.40368
H	3.29317	9.59184	20.24434
C	3.97678	5.27494	18.87274
C	2.36476	2.21006	15.41564
H	1.49594	2.87238	15.43184
C	3.75874	9.48184	18.15833
H	3.64763	10.54463	17.97249
C	7.07368	2.01401	17.84696
H	8.06173	1.87209	18.27118
C	5.30194	1.27458	16.35001
H	4.9224	0.5655	15.62478
C	3.74131	4.82492	20.15139
H	3.71906	3.75252	20.3242
C	4.16971	8.63084	17.10704
H	4.41203	9.06397	16.14443
C	4.55149	2.36905	16.76476
C	3.46373	7.06466	20.94402
H	3.24239	7.78225	21.72887
C	6.31554	3.11364	18.24688
C	3.51545	5.72188	21.21284
H	3.34749	5.34407	22.21553
C	1.91304	0.8049	15.8019
H	2.74905	0.10009	15.81787
H	1.18791	0.44954	15.0647
H	1.43279	0.80267	16.78385
C	2.99395	2.26761	14.02663
H	3.31711	3.28459	13.78973
H	2.25103	1.95825	13.28644
H	3.85347	1.59688	13.94254

Cartesian coordinates (Angstrom) of 6c neutral, optimized at the M06-2X/6-31++G level.**

45

S	4.10192	6.61738	15.62032
Cl	6.54599	4.67233	19.52518
O	2.53638	6.49822	15.59176
O	4.71554	5.3097	15.403
O	1.75883	4.37431	16.62007
O	4.44196	7.66391	14.66394
N	3.77916	4.26449	17.76344
N	3.30162	2.64622	16.33059
C	2.83966	3.79021	16.88742
C	4.8765	3.40343	17.74704
C	3.96862	6.65709	18.49553
C	3.97638	7.5016	19.65266
C	4.41654	7.26428	17.26849
C	6.68599	1.3663	17.2461
H	7.40967	0.5789	17.06403
C	4.53323	8.809	19.59474
H	4.53712	9.40367	20.50393
C	3.54875	5.30846	18.71832
C	2.56599	1.92899	15.27122
H	1.63601	2.49357	15.17218
C	5.05789	9.3001	18.43258
H	5.50264	10.28821	18.3885
C	7.00307	2.38066	18.15371
H	7.96001	2.37853	18.66462
C	5.46965	1.34615	16.56864
H	5.24098	0.5572	15.8632
C	3.03968	4.895	19.92779
H	2.7508	3.85353	20.03635
C	4.95601	8.53034	17.2535
H	5.27111	8.96422	16.31172
C	4.57466	2.37774	16.83347
C	3.45704	7.04189	20.89182
H	3.47171	7.72553	21.73597
C	6.10148	3.40984	18.40792
C	2.95647	5.7735	21.02456
H	2.55115	5.42567	21.96861
C	2.22964	0.50786	15.70864
H	3.12364	-0.11413	15.80524
H	1.58141	0.04978	14.95647
H	1.69859	0.51156	16.66462
C	3.32405	1.99731	13.94998
H	3.55217	3.03459	13.68833
H	2.70003	1.57	13.16009
H	4.25852	1.43017	13.98609
H	2.1479	5.62504	16.03704

Crystallography

The samples **(R_a)-5b**, **(R_a)-6a**, **(S_a)-6b**, and **(S_a)-6c** were isolated as a diastereomeric salt by filtration (see the Experimental Part). Each diastereomeric salt was stored in the same solvent system, which was used for crystallization, to stabilize them before the measurement. The X-ray diffraction data for colorless crystals of **(R_a)-5b**, **(R_a)-6a**, **(S_a)-6b**, and **(S_a)-6c** were collected using an XtaLAB Synergy-I diffractometer equipped with a HyPix3000 hybrid pixel array detector and a microfocused PhotonJet-I X-ray source (Cu Kα, 1.54184 Å). Absorption corrections were applied using the program CrysAlisPro 1.171.40.82a [1]. The crystal structures were solved using SHELXT [2] program and refined using the full matrix least-squares procedure with SHELXL [3] in OLEX2 (version 1.3) [4]. All non-hydrogen atoms were refined anisotropically, while hydrogen atoms were located from the Fourier difference map and refined using the "riding" model. The crystal data and refinement details for **(R_a)-5b (CCDC 2252694)**, **(R_a)-6a (CCDC 2252695)**, **(S_a)-6b (CCDC 2252696)**, and **(S_a)-6c (CCDC 2252697)** are presented in **Table S8** and **S9**. All the crystal structures contain cationic and anionic moieties (**Fig.SX1**). In the case of **6a-c**, an additional water molecule co-crystallized. The structural visualizations were created using the Mercury 2020.2.0 software package (Cambridge Crystallography Data Centre, Cambridge, UK) [5].

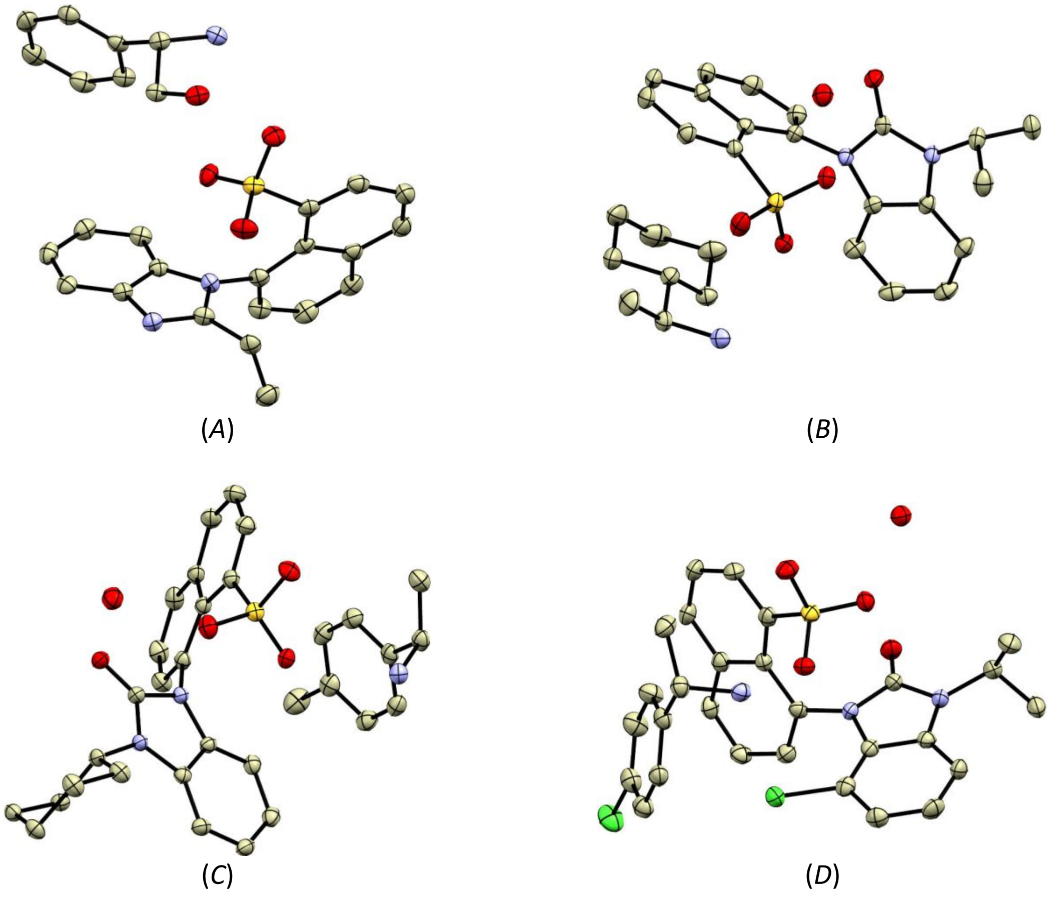

Fig.SX1 The crystal structures of **(R_a)-5b** (*A*), **(R_a)-6a** (*B*), **(S_a)-6b** (*C*), and **(S_a)-6c** (*D*). The hydrogen atoms were omitted for clarity. The Color code: carbon (light brown), chlorine (green), nitrogen (light blue), oxygen (red), and sulfur (yellow). The thermal ellipsoids were drawn at a 50% probability level.

Table S8: Crystal data and structure refinement for **(R_a)-5b** and **(R_a)-6a**.

	**(R_a)-5b**	**(R_a)-6a**
Formula	$C_{27}H_{27}N_3O_4S$	$C_{28}H_{37}N_3O_5S$
M_r	489.57	527.66
Crystal system	monoclinic	orthorhombic
Space group	$P2_1$	$P2_12_12_1$
T/K	90.0(2)	100.0(2)
a (Å)	9.3567(2)	8.5291(2)
b (Å)	8.6393(2)	11.4419(2)
c (Å)	15.1148(4)	27.8432(5)
α (°)	90	90
β (°)	105.847(3)	90
γ (°)	90	90
V (Å^3)	1175.37(5)	2717.19(9)
Z	2	4
λ (Å), Cu Kα	1.54184	1.54184
D_{calc} (g·cm^{-3})	1.383	1.290
μ (mm^{-1})	1.557	1.406
F (000)	516.0	1128.0
Independent reflections	4260 [R_{int} = 0.0391, R_{sigma} = 0.0460]	4930 [R_{int} = 0.0382, R_{sigma} = 0.0438]
Data/restraints/parameters	4260/1/319	4930/0/341
Goodness-of-fit on F^2	1.082	1.052
Final R indices [$I > 2\sigma(I)$]	R_1 = 0.0358, wR_2 = 0.0925	R_1 = 0.0350, wR_2 = 0.0884
Final R indices (all data)	R_1 = 0.0384, wR_2 = 0.0938	R_1 = 0.0395, wR_2 = 0.0907
Flack parameter	-0.028(13)	-0.010(11)
CCDC no.		

Table S9: Crystal data and structure refinement for **(S_a)-6b** and **(S_a)-6c**.

	**(S_a)-6b**	**(S_a)-6c**
Formula	$C_{33}H_{37}N_3O_5S$	$C_{28}H_{29}Cl_2N_3O_5S$
M_r	575.70	590.50
Crystal system	orthorhombic	orthorhombic
Space group	$P2_12_12_1$	$P2_12_12_1$
T/K	100.0(2)	100.0(2)
a (Å)	8.99860(10)	8.8910(3)
b (Å)	11.8965(2)	11.0012(4)
c (Å)	26.8080(4)	28.3232(10)
α (°)	90	90
β (°)	90	90
γ (°)	90	90
V (Å^3)	2869.85(7)	2770.34(17)
Z	4	4
λ (Å), Cu Kα	1.54184	1.54184
D_{calc} (g·cm^{-3})	1.332	1.416
μ (mm^{-1})	1.381	3.180
F (000)	1224.0	1232.0
Independent reflections	5216 [R_{int} = 0.0381, R_{sigma} = 0.0381]	5022 [R_{int} = 0.0740, R_{sigma} = 0.0646]
Data/restraints/parameters	5216/0/376	5022/3/364
Goodness-of-fit on F^2	1.035	1.072
Final R indices [$I > 2\sigma(I)$]	R_1 = 0.0308, wR_2 = 0.0748	R_1 = 0.0533, wR_2 = 0.1380
Final R indices (all data)	R_1 = 0.0347, wR_2 = 0.0764	R_1 = 0.0619, wR_2 = 0.1463
Flack parameter	-0.003(9)	0.019(15)
CCDC no.		

[1] Rigaku Oxford Diffraction (2020) CrysAlisPro 1.171.40.82a.
[2] G. M. Sheldrick, Acta Crystallogr. Sect. A 2015, 71, 3–8.
[3] L. J. Bourhis, O. V. Dolomanov, R. J. Gildea, J. A. K. Howard, H. Puschmann, Acta Crystallogr. Sect. A 2015, 71, 59–75.
[4] O. V. Dolomanov, L. J. Bourhis, R. J. Gildea, J. A. K. Howard, H. Puschmann, J. Appl. Crystallogr. 2009, 42, 339–341.
[5] (a) C. F. Macrae, I. Sovago, S. J. Cottrell, P. T. A. Galek, P. McCabe, E. Pidcock, M. Platings, G. P. Shields, J. S. Stevens, M. Towler and P. A. Wood, J. Appl. Cryst., 2020, 53, 226-235., (b) C. R. Groom, I. J. Bruno, M. P. Lightfoot and S. C. Ward, Acta Cryst. B 2016, 72, 171-179.
